# Supplementary material for: A framework for reconstructing SARS-CoV-2 transmission dynamics using excess mortality data
Source: Nat Commun. 2022 May 31;13:3015. doi: 10.1038/s41467-022-30711-y (PMC9156676; doi:10.1038/s41467-022-30711-y)
Supplement: Supplementary file 1 — Supplementary Information [file 41467_2022_30711_MOESM1_ESM.pdf]

Supplementary Information

A framework for reconstructing SARS-CoV-2 transmission dynamics using excess mortality data

Author list

Mahan Ghafari[1*†], Oliver J Watson[2†], Ariel Karlinsky[3], Luca Ferretti[4], Aris Katzourakis[1*]

Affiliations

[1] Department of Zoology, University of Oxford, Oxford, UK

[2] Department of Infectious Disease Epidemiology, Imperial College London, London, UK

[3] Department of Economics, Hebrew University of Jerusalem, Jerusalem, Israel

[4] Big Data Institute, Li Ka Shing Centre for Health Information and Discovery, Nuffield Department of Medicine, University of Oxford, Oxford, UK

*Corresponding author.

Email: mahan.ghafari@zoo.ox.ac.uk (MG); aris.katzourakis@zoo.ox.ac.uk (AK)

†These authors contributed equally.

Supplementary Tables

Supplementary Table 1: The overall attack rate (in percentage) by the end of May 2020 (wave 1), August 2020 (wave 2), January 2021 (wave 3), June 2021 (wave 4), and the week ending on 22 October 2021 (wave 5).

Province	Wave 1	Wave 2	Wave 3	Wave 4	Wave 5
Alborz	5.32 (5.44 - 5.16)	10.50 (10.27 - 10.48)	27.44 (25.99 - 29.10)	45.49 (38.88 - 51.17)	64.09 (51.07 - 81.97)
Ardabil	11.18 (9.90 - 11.52)	23.57 (20.61 - 26.34)	51.23 (45.54 - 54.60)	69.27 (60.02 - 77.94)	92.55 (76.19 - 112.14)
Bushehr	3.20 (2.53 - 3.24)	11.52 (8.40 - 11.39)	25.13 (20.63 - 25.25)	40.97 (32.96 - 45.54)	61.29 (44.63 - 82.10)
Chahar Mahaal and Bakhtiari	2.79 (2.41 - 2.63)	6.46 (4.68 - 5.67)	26.46 (21.57 - 27.75)	37.69 (32.88 - 44.16)	53.05 (43.49 - 73.44)
East Azerbaijan	5.44 (5.50 - 5.14)	11.68 (11.86 - 11.45)	39.85 (37.39 - 42.29)	60.36 (52.28 - 64.14)	84.92 (69.06 - 103.05)
Fars	1.64 (1.61 - 1.59)	6.51 (6.04 - 7.08)	25.69 (23.57 - 29.32)	38.77 (33.97 - 43.35)	56.63 (42.64 - 71.82)
Gilan	15.51 (15.13 - 17.90)	17.50 (17.26 - 20.12)	28.36 (27.51 - 31.21)	36.79 (34.53 - 40.22)	52.62 (45.21 - 67.60)
Golestan	20.27 (18.89 - 21.60)	33.20 (30.69 - 36.89)	55.32 (48.44 - 56.16)	71.67 (61.89 - 75.25)	105.43 (87.31 - 125.82)
Hamedan	3.74 (3.79 - 3.78)	10.69 (10.87 - 11.16)	28.74 (26.68 - 29.71)	45.23 (40.47 - 48.63)	63.97 (53.45 - 76.72)
Hormozgan	3.51 (3.55 - 4.10)	12.58 (11.96 - 13.92)	21.11 (20.27 - 22.03)	32.48 (27.99 - 35.42)	48.52 (38.76 - 64.10)
Ilam	4.49 (4.13 - 4.23)	10.78 (10.17 - 10.55)	34.75 (31.70 - 36.22)	44.19 (40.23 - 47.35)	61.35 (51.01 - 73.74)
Isfahan	5.58 (4.92 - 5.28)	9.95 (8.66 - 9.62)	30.09 (26.10 - 30.96)	42.93 (37.36 - 45.99)	65.90 (51.80 - 81.46)
Kerman	2.10 (2.17 - 2.15)	8.11 (7.99 - 9.06)	23.45 (22.19 - 24.80)	32.84 (29.26 - 34.56)	50.58 (40.64 - 63.22)
Kermanshah	4.08 (4.39 - 4.31)	9.97 (9.86 - 9.13)	30.16 (28.50 - 32.97)	41.38 (37.50 - 46.08)	66.67 (54.07 - 86.87)
Khuzestan	11.17 (10.51 - 13.65)	26.08 (23.70 - 29.49)	40.38 (36.19 - 42.13)	68.54 (58.79 - 76.98)	98.48 (79.82 - 120.59)
Kohgiluyeh and Boyer-Ahmad	4.24 (3.88 - 3.79)	9.37 (8.61 - 8.72)	27.38 (24.45 - 27.12)	42.16 (36.62 - 46.16)	59.41 (48.25 - 74.93)
Kurdistan	7.66 (6.86 - 7.83)	17.13 (16.40 - 18.52)	45.93 (43.35 - 55.61)	59.55 (54.04 - 69.37)	80.05 (66.82 - 103.86)
Lorestan	5.37 (5.64 - 5.41)	11.81 (12.20 - 12.09)	28.87 (27.28 - 30.13)	40.94 (36.45 - 43.29)	56.51 (47.10 - 68.36)
Markazi	5.86 (5.45 - 5.22)	11.31 (10.48 - 10.60)	33.26 (31.29 - 33.89)	47.89 (42.32 - 51.57)	64.71 (53.01 - 75.59)
Mazandaran	11.19 (10.15 - 11.53)	18.16 (16.62 - 18.97)	31.36 (29.63 - 32.70)	40.39 (37.42 - 42.43)	58.36 (48.01 - 69.78)
North Khorasan	6.46 (6.06 - 5.73)	14.52 (12.58 - 13.86)	36.11 (32.34 - 40.32)	48.61 (41.48 - 53.53)	77.73 (61.94 - 100.78)
Qazvin	12.92 (12.21 - 13.66)	19.79 (18.21 - 20.70)	46.85 (41.92 - 50.43)	64.95 (55.77 - 72.23)	85.97 (69.52 - 106.44)
Qom	14.87 (14.23 - 18.22)	20.10 (18.55 - 24.31)	40.20 (36.16 - 47.18)	51.89 (45.12 - 61.98)	84.74 (66.12 - 116.36)
Razavi Khorasan	6.77 (6.27 - 6.66)	19.33 (17.53 - 21.33)	43.41 (38.66 - 45.49)	57.06 (49.79 - 64.07)	90.19 (71.76 - 113.32)
Semnan	4.12 (4.59 - 4.19)	8.27 (9.25 - 8.31)	30.87 (29.84 - 31.08)	42.82 (39.75 - 45.06)	57.26 (49.85 - 65.92)
Sistan and Baluchistan	1.24 (1.23 - 1.11)	5.02 (5.51 - 5.35)	14.02 (14.85 - 14.14)	26.46 (22.98 - 32.57)	46.60 (35.38 - 67.60)
South Khorasan	3.11 (2.94 - 3.49)	5.72 (5.26 - 6.53)	21.59 (19.20 - 23.92)	30.87 (27.58 - 33.24)	41.27 (34.65 - 48.50)
Tehran	7.69 (8.31 - 8.07)	12.67 (14.37 - 13.17)	32.42 (33.46 - 34.66)	51.17 (46.13 - 54.27)	77.06 (65.08 - 93.99)
West Azerbaijan	4.06 (3.85 - 4.34)	10.23 (8.87 - 10.50)	37.85 (34.37 - 42.99)	52.94 (45.84 - 60.46)	75.66 (60.25 - 98.53)
Yazd	11.26 (12.05 - 11.49)	17.10 (18.44 - 17.86)	53.88 (51.44 - 59.97)	64.11 (59.99 - 72.87)	80.50 (71.43 - 93.68)
Zanjan	6.85 (7.59 - 7.15)	14.10 (14.18 - 14.59)	38.23 (35.99 - 41.37)	56.79 (51.31 - 63.59)	73.11 (60.85 - 87.84)

Supplementary Figures

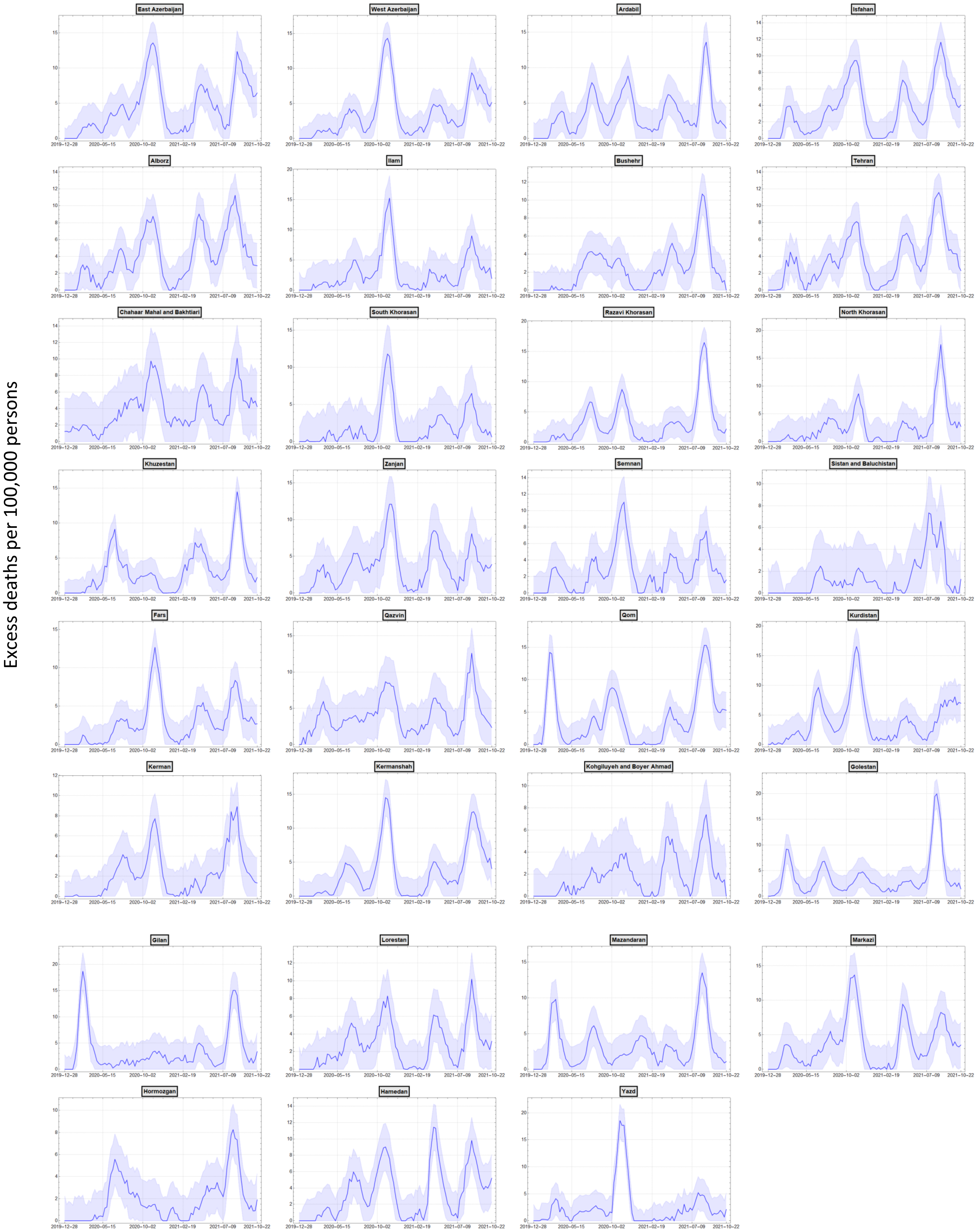

Supplementary Figure 1: One-month average of weekly excess mortality per 100,000 persons per province over time. The lines show the excess mortality estimates based on the linear regression model with central line and shaded area representing the median and 95% forecast intervals. Note that the number of confirmed COVID-19 deaths are not reported at the province level in Iran.

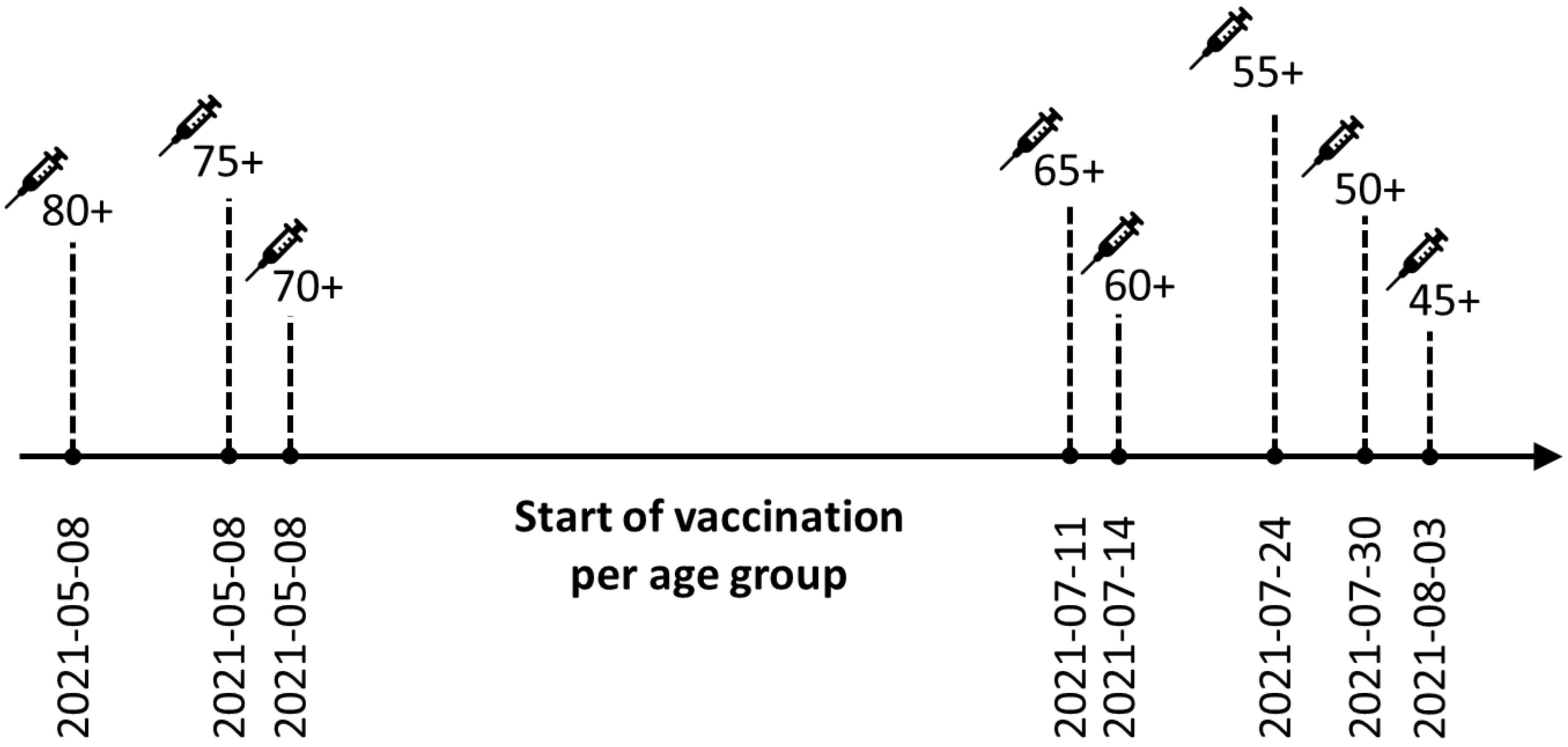

Supplementary Figure 2: Iran COVID-19 vaccination rollout dates per age group based on announcements made by MoHME (*behdasht.gov.ir*). Starting dates of vaccination in each age-group is highlighted with a vertical dashed line.

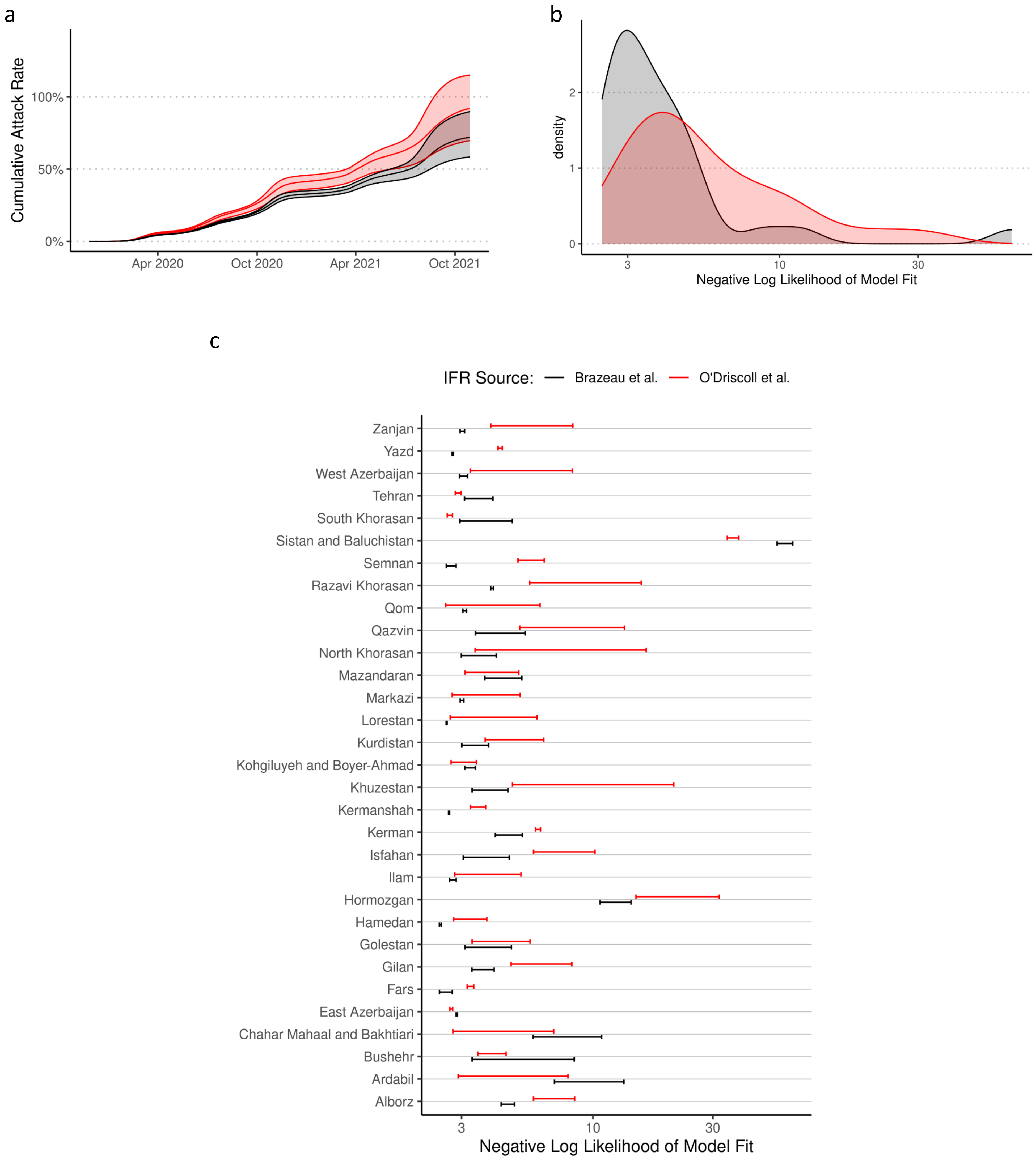

Supplementary Figure 3: Likelihood distribution of the modeled attack rates per province against seroprevalence data. (a) Cumulative nationwide attack rates of SARS-CoV-2. Shaded area shows the variation in estimates from the three sets of model assumptions used for estimating attack rates. (b, c) Likelihood distribution of the modelled attack rates per province against seroprevalence data [1] using the O'Drsicoll et al. [2] (red) and Brazeau et al. [3] (black) age-stratified infection fatality rate estimates. Lines shown in (c) represent the range of likelihood values across the optimistic, central, and worst-case scenarios.

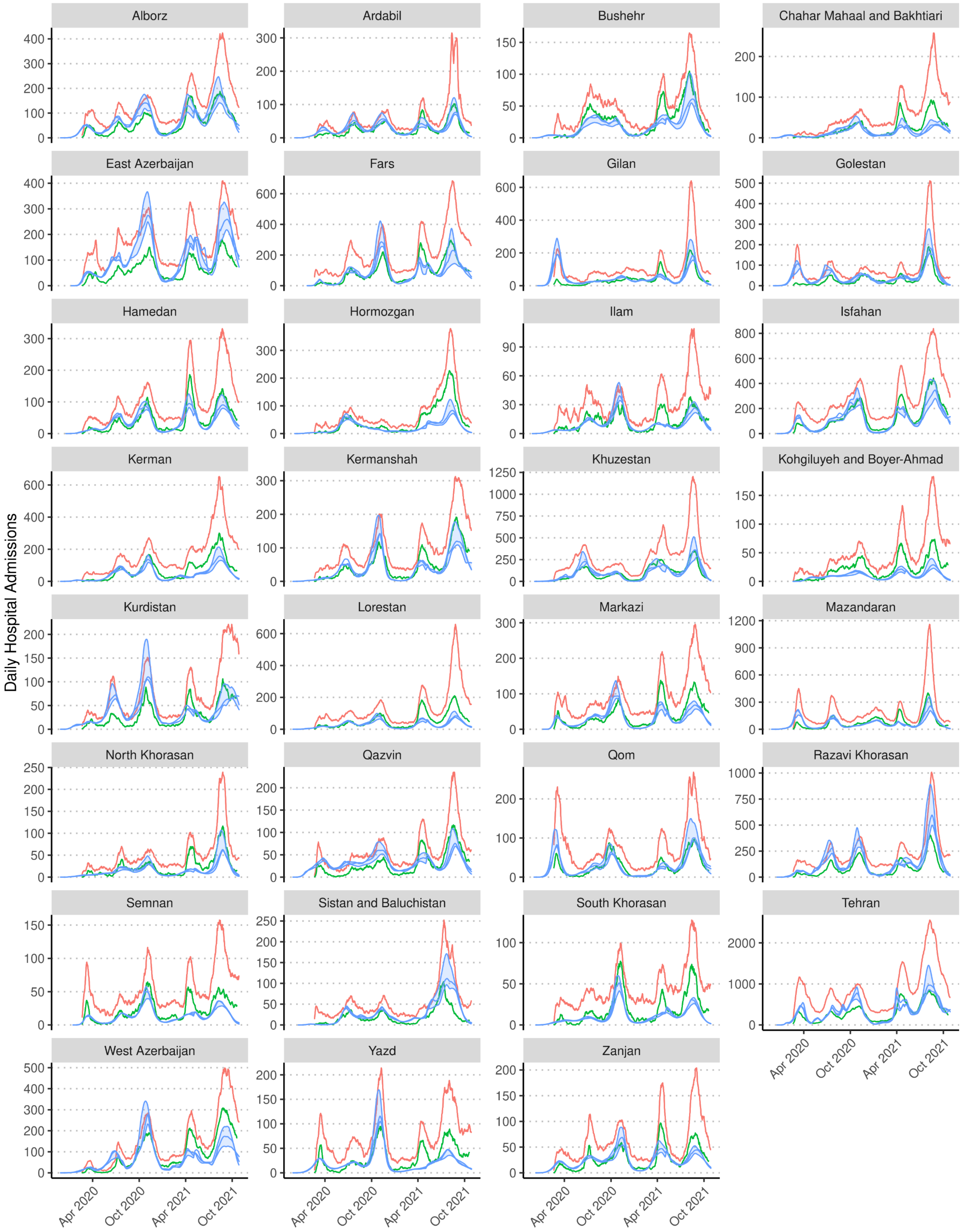

Supplementary Figure 4: **Confirmed (green), suspected (red), and modelled (blue) daily hospital admissions per province over time.** Shaded area in blue shows the variation in modelled daily hospital admissions from the three sets of model assumptions used for estimating attack rates. The central line in blue shows the central scenario and the light blue bands show the range reflected by the optimistic and worst-case scenarios.

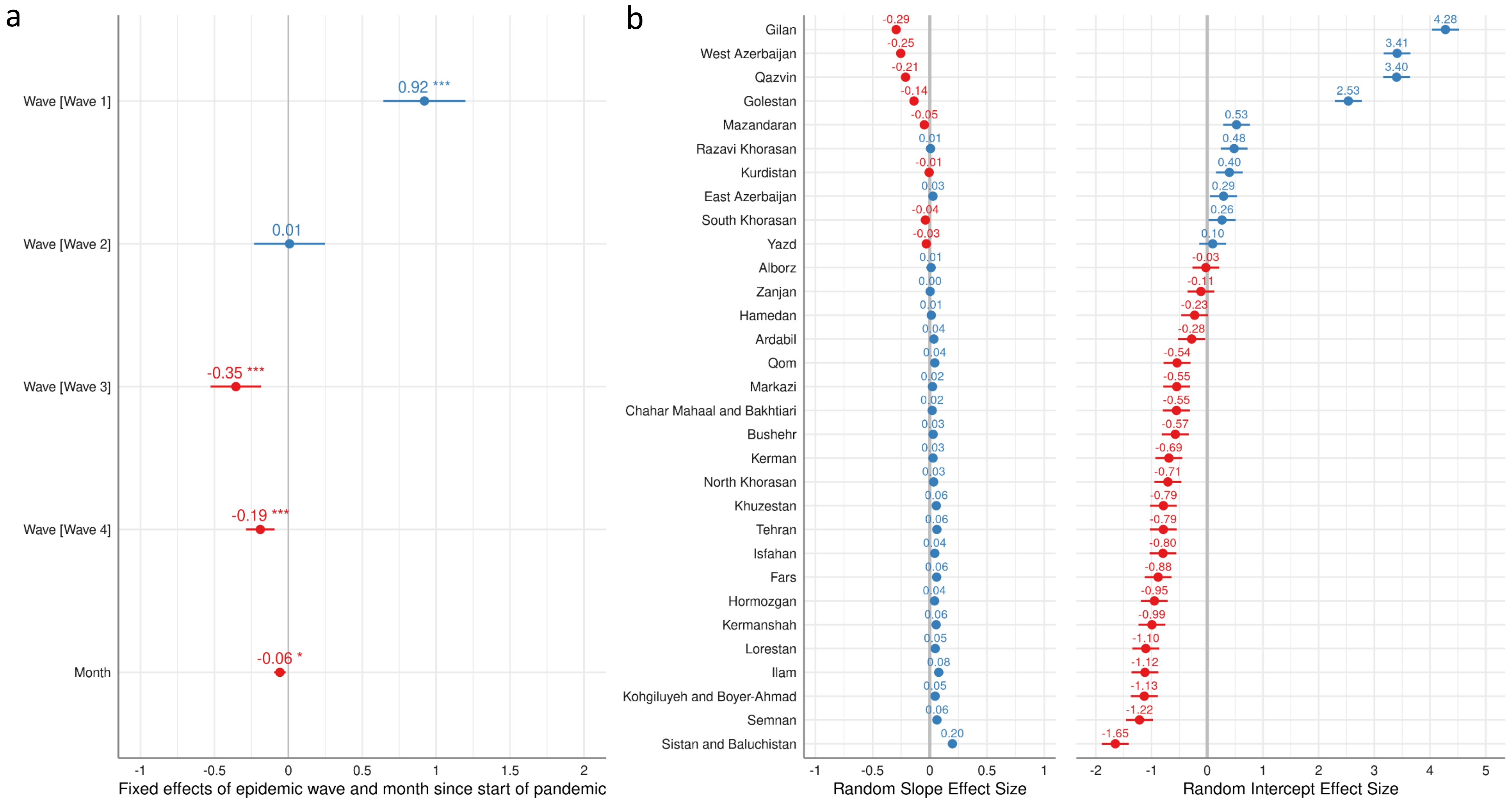

Supplementary Figure 5: **Mixed effect model results investigating the relationship between model inferred hospitalisations and observed daily hospital admissions.** In (a) the fixed effect sizes are shown, with positive point estimates in blue and negative point estimates in red, with significant effects indicated with asterisks (*p<0.05, **p<0.01 and ***p<0.001%). In (b) the random effect sizes are shown for each province ranked by the size of the random intercept for each province. In both (a) and (b), the lines represent the 95% confidence intervals from the mixed effects model. Up to three asterisks are shown to confer different levels of significance (95%, 99%, 99.9%) for the fixed effects. The confidence intervals reflect the results of the mixed effects model, for which 18649 hospitalisation estimates were available across 31 provinces. This is also the information for what statistical test conducted (mixed effects linear regression). The specific p-values for the fixed effects are: Wave 1 = 4.77×10^{-10}, Wave 2 = 1.0, Wave 3 = 2.89×10^{-4}, Wave 4 = 7.36×10^{-4}, and Month = 2.31×10^{-2}, which applied Bonferroni corrections for multiple comparisons.

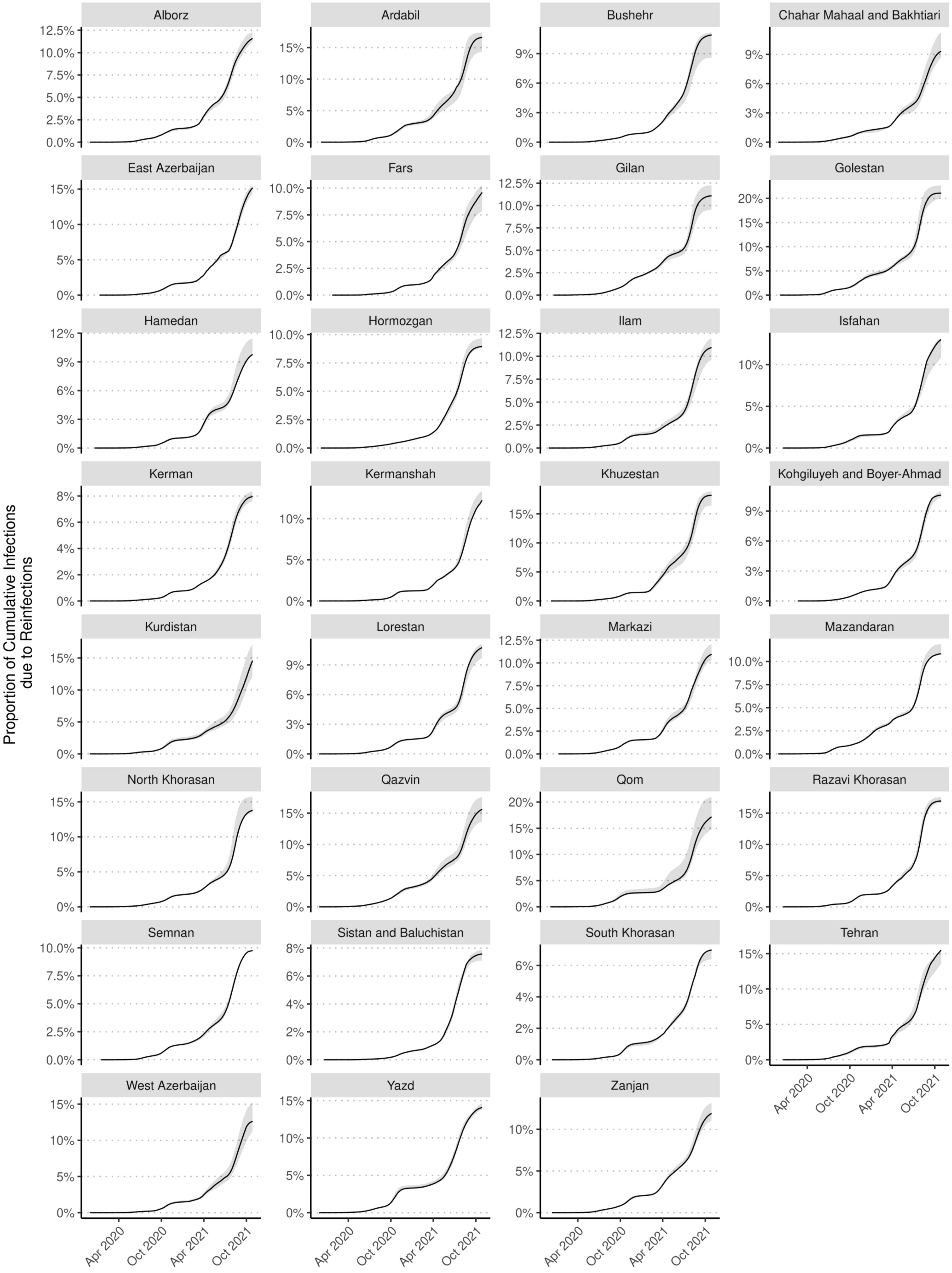

Supplementary Figure 6: Estimated proportion of infections due to reinfections per province over time. Shaded area shows the variation in estimates from the three sets of model assumptions used for estimating attack rates. The central line shows the central scenario and the bands show the range reflected by the optimistic and worst-case scenarios.

a

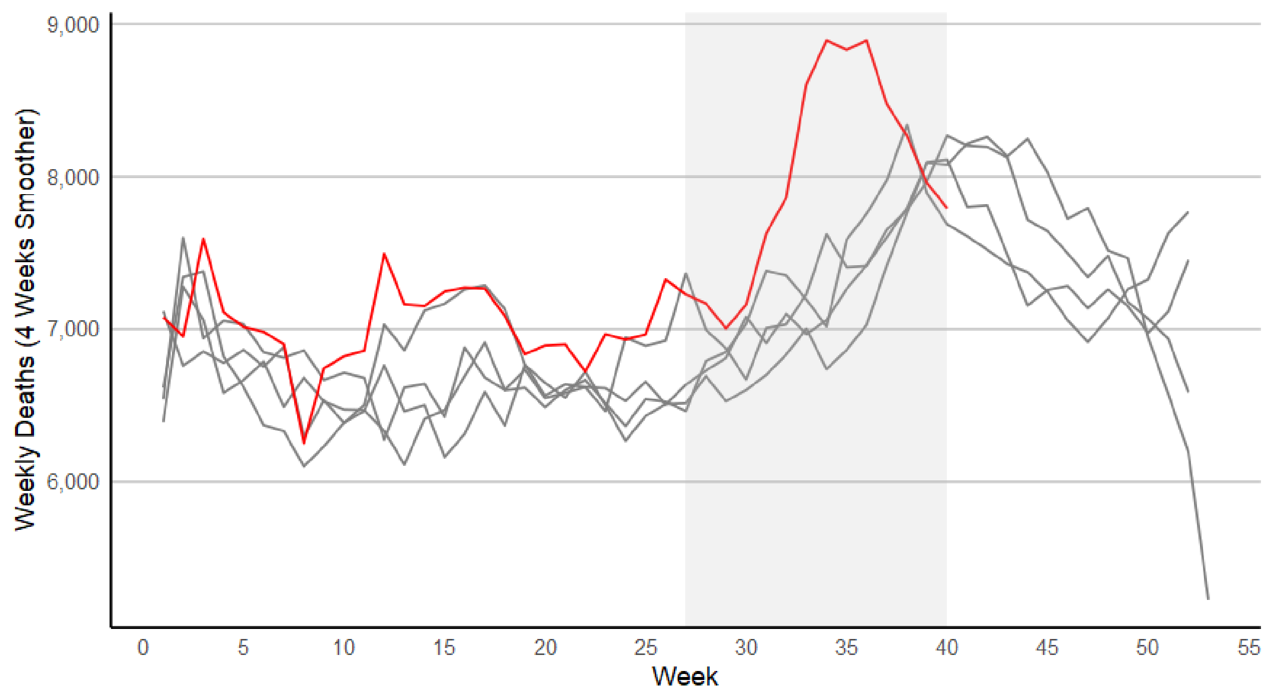

b

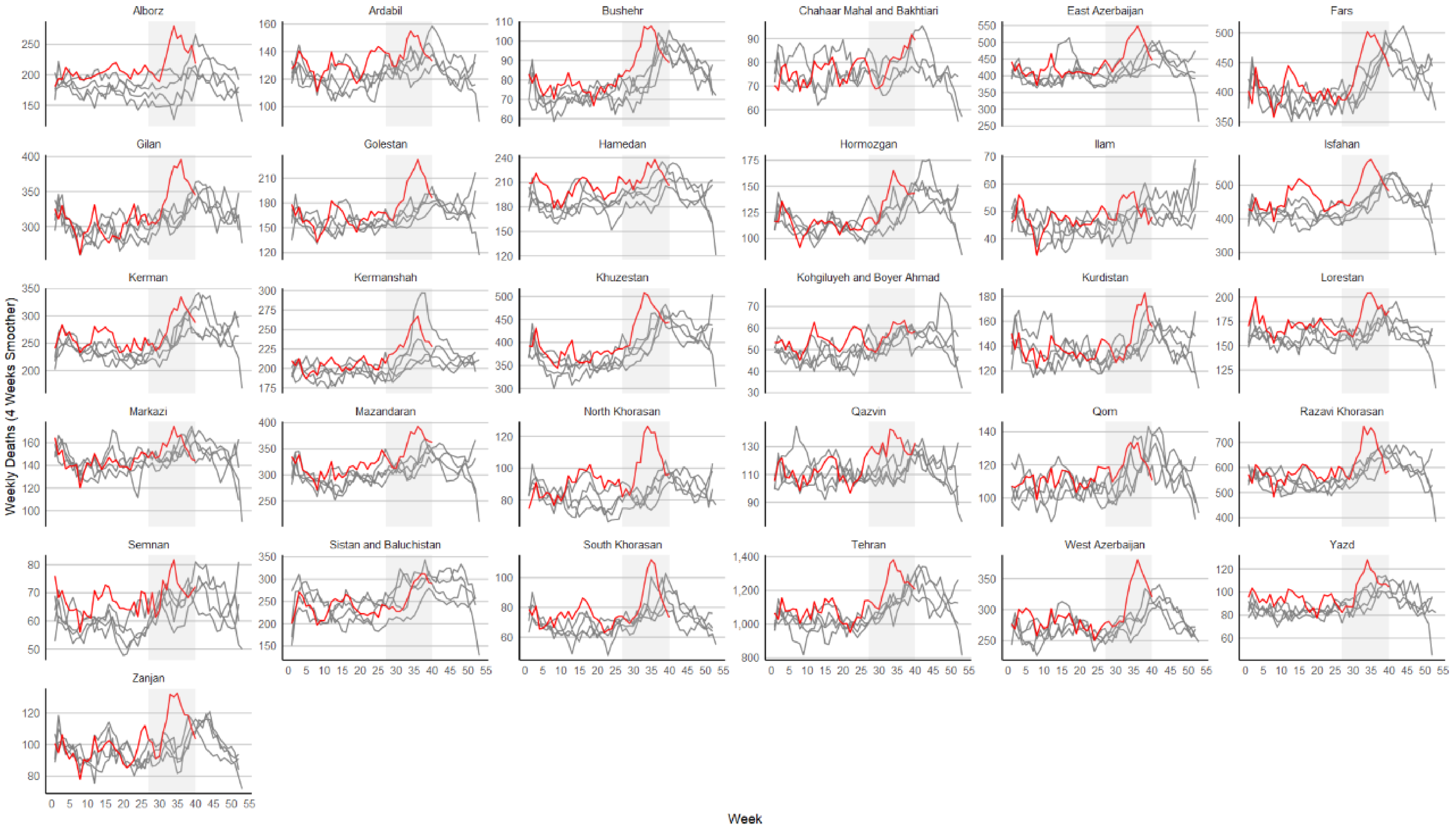

Supplementary Figure 7: One-month average weekly all-cause mortality data from 2015-03-21 to 2019-12-27. (a) At the national level and (b) at the province level. All-cause mortality from 2015 to 2018 are shown gray and all-cause mortality during 2019 is shown in Red. Shaded area in gray highlights the weeks in autumn 2019 (2019-09-27 to 2019-12-27).

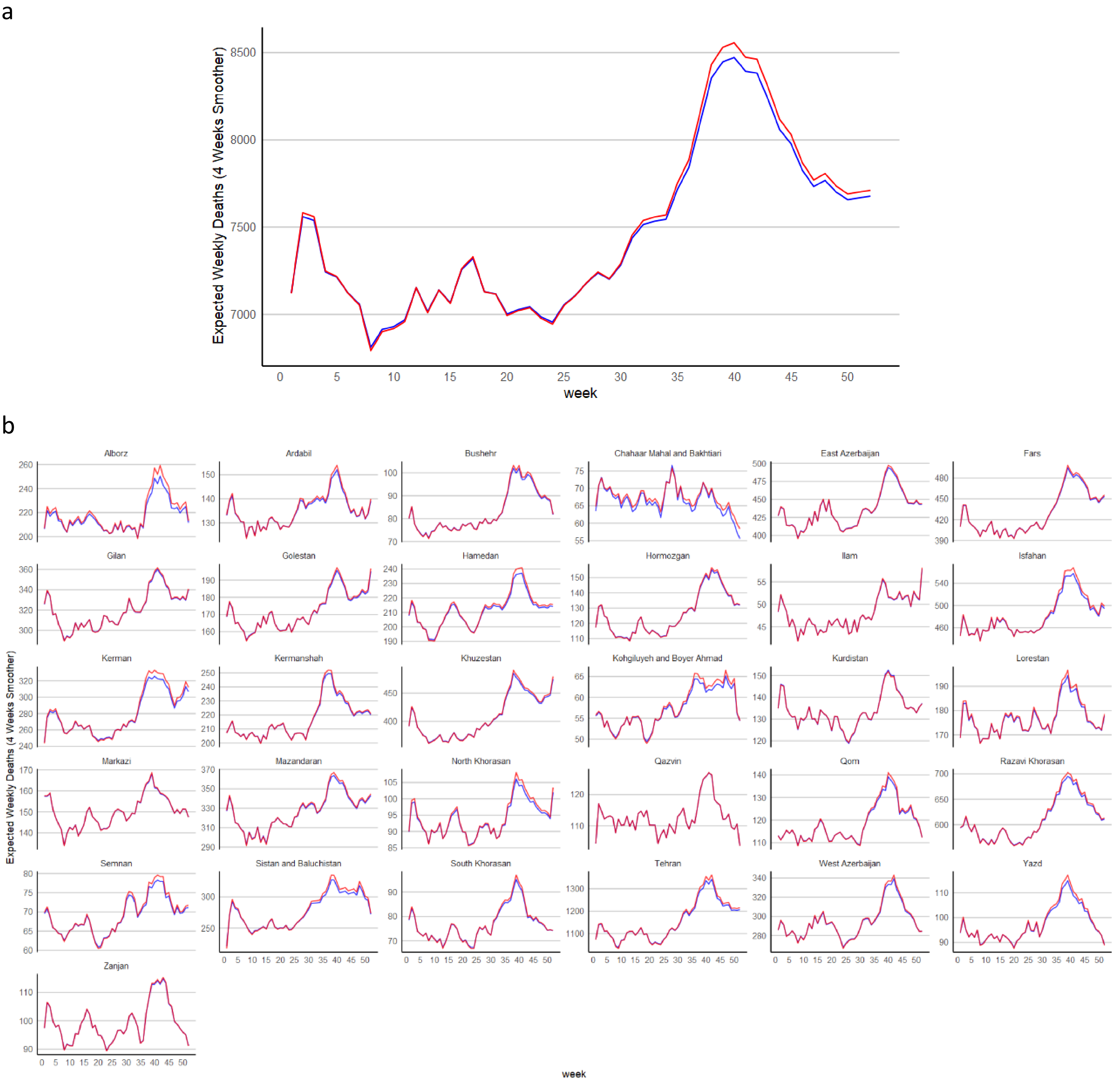

Supplementary Figure 8: **Comparison between the expected one-month average of weekly excess mortality per 100,000 persons.** (a) At the national level and (b) at the per-province over time using a linear regression (blue) and quasi-Poisson (red) model.

Supplementary References

1. Khalagi, K., et al., *Prevalence of COVID-19 in Iran: results of the first survey of the Iranian COVID-19 Serological Surveillance programme.* Clinical Microbiology and Infection, 2021. **27**(11): p. 1666-1671.
2. O'Driscoll, M., et al., *Age-specific mortality and immunity patterns of SARS-CoV-2.* Nature, 2020.
3. Brazeau, N., et al., *Report 34: COVID-19 infection fatality ratio: estimates from seroprevalence.* Imperial College London COVID-19 Response Team, 2020.
4. Poustchi, H., et al., *SARS-CoV-2 antibody seroprevalence in the general population and high-risk occupational groups across 18 cities in Iran: a population-based cross-sectional study.* Lancet Infect Dis, 2021. **21**(4): p. 473-481.
