# Supplementary material for: A framework for reconstructing SARS-CoV-2 transmission dynamics using excess mortality data
Source: Nat Commun. 2022 May 31;13:3015. doi: 10.1038/s41467-022-30711-y (PMC9156676; doi:10.1038/s41467-022-30711-y)
Supplement: Supplementary file 3 — Description of Additional Supplementary Files [file 41467_2022_30711_MOESM3_ESM.pdf]

Description of Additional Supplementary Files

Supplementary Data 1: Transmission model fits per province using weekly excess mortality data (as at week ending on 2021-10-21) assuming the central scenario using the Brazeau et al. IFR [3] estimate. For each province, there are 5 panels showing the model fits for: (i) The time-varying effective reproduction number, R_{eff}, in green. The curve in blue shows the predicted decrease in R_{eff} due to increasing immunity in the population as a result of people being infected by COVID-19. Dark green and blue show the 50% confidence intervals and light green and blue show the 95% confidence intervals. A horizontal dashed line is shown at $R_{eff} < 1$ indicates a slowing epidemic in which new infections are not increasing. $R_{eff} > 1$ indicates a growing epidemic in which new infections are increasing over time. (ii) Observed (black circles) and modelled (red line) weekly deaths, with the thick red line depicting the median, the faint depicting individual draws from the posterior and the 95% credible interval shown with dashed black lines. (iii) Modelled cumulative deaths over time. (iv) Modelled seroprevalence (black line) and point estimates by Khalagi et al. [1] (red) and Poustchi et al. [4] (green). Shaded area shows the 95% confidence in modelled seroprevalence. Vertical and horizontal lines around each point estimate represents uncertainty in the reported measurement. (v) Modelled proportion of people exposed to SARS-CoV-2 over time. In both (iv) and (v), the central line depicts the median and the lighter shaded band shows the 95% credible interval.

Supplementary Data 2: Transmission model fits per province using weekly excess mortality data (as at week ending on 2021-10-21) assuming the optimistic scenario using the Brazeau et al. IFR [3] estimate. For each province, there are 5 panels showing the model fits for: (i) The time-varying effective reproduction number, R_{eff}, in green. The curve in blue shows the predicted decrease in R_{eff} due to increasing immunity in the population as a result of people being infected by COVID-19. Dark green and blue show the 50% confidence intervals and light green and blue show the 95% confidence intervals. A horizontal dashed line is shown at $R_{eff} < 1$ indicates a slowing epidemic in which new infections are not increasing. $R_{eff} > 1$ indicates a growing epidemic in which new infections are increasing over time. (ii) Observed (black circles) and modelled (red line) weekly deaths, with the thick red line depicting the median, the faint depicting individual draws from the posterior and the 95% credible interval shown with dashed black lines. (iii) Modelled cumulative deaths over time. (iv) Modelled seroprevalence (black line) and point estimates by Khalagi et al. [1] (red) and Poustchi et al. [4] (green). Shaded area shows the 95% confidence in modelled seroprevalence. Vertical and horizontal lines around each point estimate represents uncertainty in the reported measurement. (v) Modelled proportion of people exposed to SARS-CoV-2 over time. In both (iv) and (v), the central line depicts the median and the lighter shaded band shows the 95% credible interval.

Supplementary Data 3: Transmission model fits per province using weekly excess mortality data (as at week ending on 2021-10-21) assuming the worst-case scenario using the Brazeau et al. IFR [3] estimate. For each province, there are 5 panels showing the model fits for: (i) The time-varying effective reproduction number, R_{eff}, in green. The curve in blue shows the predicted decrease in R_{eff} due to increasing immunity in the population as a result of people being infected by COVID-19. Dark green and blue show the 50% confidence intervals and light green and blue show the 95% confidence intervals. A horizontal dashed line is shown at $R_{eff} < 1$ indicates a slowing epidemic in which new infections are not increasing. $R_{eff} > 1$ indicates a growing epidemic in which new infections are increasing over time. (ii) Observed (black circles) and modelled (red line) weekly deaths, with the thick red line depicting the median, the faint depicting individual draws from the posterior and the 95% credible interval shown with dashed black lines. (iii) Modelled cumulative deaths over time. (iv) Modelled seroprevalence (black line) and point estimates by Khalagi et al. [1] (red) and Poustchi et al. [4] (green). Shaded area shows the 95% confidence in modelled seroprevalence. Vertical and horizontal lines around each point estimate represents uncertainty in the reported measurement. (v) Modelled proportion of people exposed to SARS-CoV-2 over time. In both (iv) and (v), the central line depicts the median and the lighter shaded band shows the 95% credible interval.

Supplementary Data 4: Transmission model fits per province using weekly excess mortality data (as at week ending on 2021-10-21) assuming the central scenario using the O'Driscoll et al. IFR [2] estimate. For each province, there are 5 panels showing the model fits for: (i) The time-varying effective reproduction number, R_{eff}, in green. The curve in blue shows the predicted decrease in R_{eff} due to increasing immunity in the population as a result of people being infected by COVID-19. Dark green and blue show the 50% confidence intervals and light green and blue show the 95% confidence intervals. A horizontal dashed line is shown at $R_{eff} < 1$ indicates a slowing epidemic in which new infections are not increasing. $R_{eff} > 1$ indicates a growing epidemic in which new infections are increasing over time. (ii) Observed (black circles) and modelled (red line) weekly deaths, with the thick red line depicting the median, the faint depicting individual draws from the posterior and the 95% credible interval shown with dashed black lines. (iii) Modelled cumulative deaths over time. (iv) Modelled seroprevalence (black line) and point estimates by Khalagi et al. [1] (red) and Poustchi et al. [4] (green). Shaded area shows the 95% confidence in modelled seroprevalence. Vertical and horizontal lines around each point estimate represents uncertainty in the reported measurement. (v) Modelled proportion of people exposed to SARS-CoV-2 over time. In both (iv) and (v), the central line depicts the median and the lighter shaded band shows the 95% credible interval.

Supplementary Data 5: Transmission model fits per province using weekly excess mortality data (as at week ending on 2021-10-21) assuming the optimistic scenario using the O'Driscoll et al. IFR [2] estimate. For each province, there are 5 panels showing the model fits for: (i) The time-varying effective reproduction number, R_{eff}, in green. The curve in blue shows the predicted decrease in R_{eff} due to increasing immunity in the population as a result of people being infected by COVID-19. Dark green and blue show the 50% confidence intervals and light green and blue show the 95% confidence intervals. A horizontal dashed line is shown at $R_{eff} <$

1 indicates a slowing epidemic in which new infections are not increasing. R_{eff} > 1 indicates a growing epidemic in which new infections are increasing over time. (ii) Observed (black circles) and modelled (red line) weekly deaths, with the thick red line depicting the median, the faint depicting individual draws from the posterior and the 95% credible interval shown with dashed black lines. (iii) Modelled cumulative deaths over time. (iv) Modelled seroprevalence (black line) and point estimates by Khalagi et al. [1] (red) and Poustchi et al. [4] (green). Shaded area shows the 95% confidence in modelled seroprevalence. Vertical and horizontal lines around each point estimate represents uncertainty in the reported measurement. (v) Modelled proportion of people exposed to SARS-CoV-2 over time. In both (iv) and (v), the central line depicts the median and the lighter shaded band shows the 95% credible interval.

Supplementary Data 6: Transmission model fits per province using weekly excess mortality data (as at week ending on 2021-10-21) assuming the worst-case scenario using the O'Driscoll et al. IFR [2] estimate. For each province, there are 5 panels showing the model fits for: (i) The time-varying effective reproduction number, R_{eff}, in green. The curve in blue shows the predicted decrease in R_{eff} due to increasing immunity in the population as a result of people being infected by COVID-19. Dark green and blue show the 50% confidence intervals and light green and blue show the 95% confidence intervals. A horizontal dashed line is shown at R_{eff} < 1 indicates a slowing epidemic in which new infections are not increasing. R_{eff} > 1 indicates a growing epidemic in which new infections are increasing over time. (ii) Observed (black circles) and modelled (red line) weekly deaths, with the thick red line depicting the median, the faint depicting individual draws from the posterior and the 95% credible interval shown with dashed black lines. (iii) Modelled cumulative deaths over time. (iv) Modelled seroprevalence (black line) and point estimates by Khalagi et al. [1] (red) and Poustchi et al. [4] (green). Shaded area shows the 95% confidence in modelled seroprevalence. Vertical and horizontal lines around each point estimate represents uncertainty in the reported measurement. (v) Modelled proportion of people exposed to SARS-CoV-2 over time. In both (iv) and (v), the central line depicts the median and the lighter shaded band shows the 95% credible interval.
