# Supplementary figures and images for: A framework for reconstructing SARS-CoV-2 transmission dynamics using excess mortality data
Source: Nat Commun. 2022 May 31;13:3015. doi: 10.1038/s41467-022-30711-y (PMC9156676; doi:10.1038/s41467-022-30711-y)

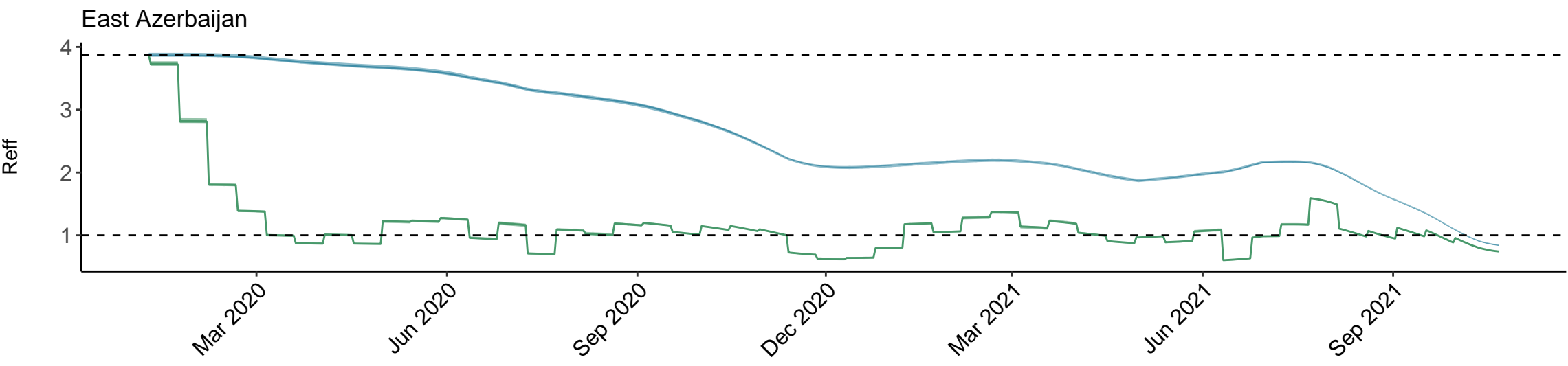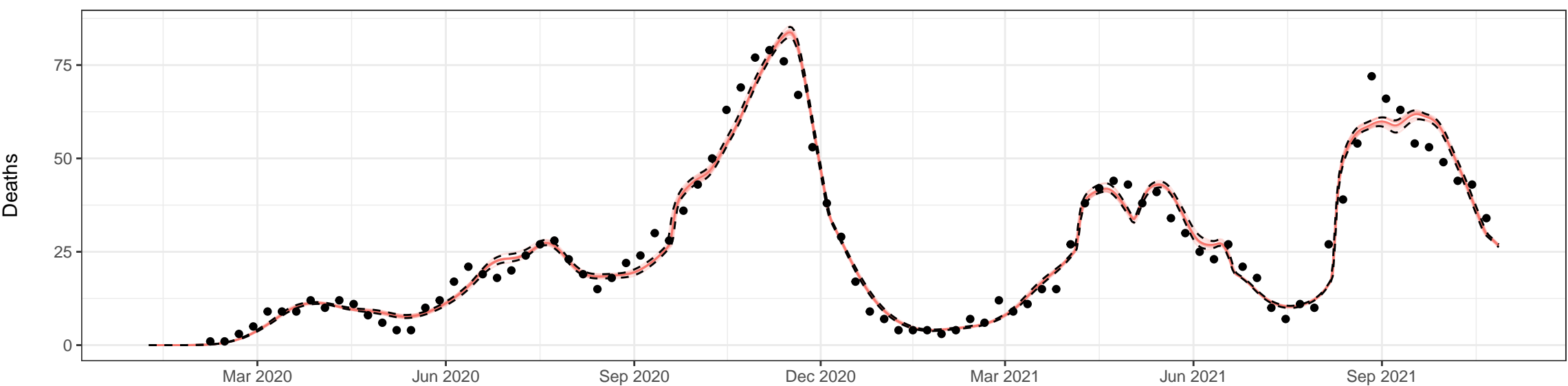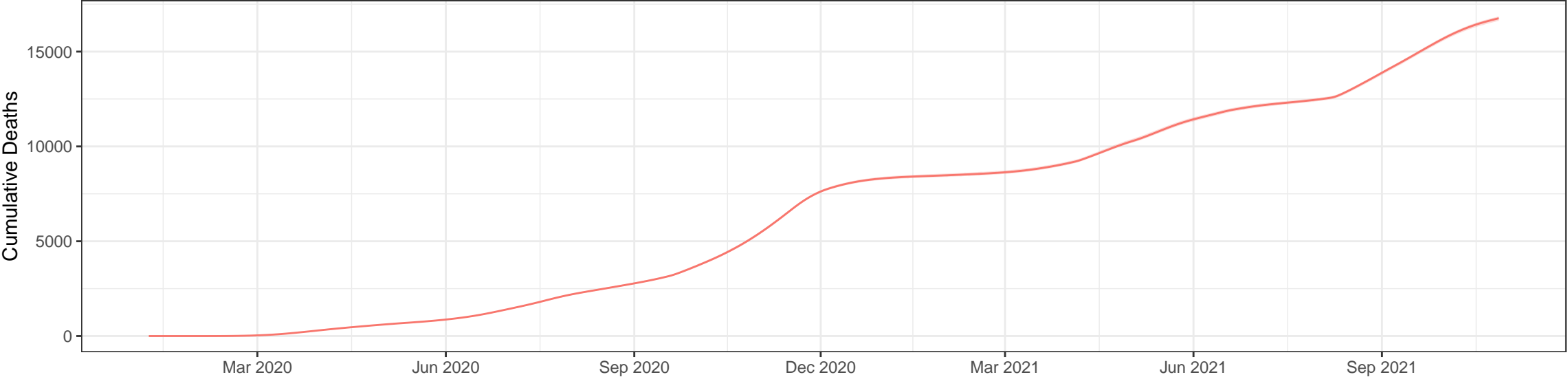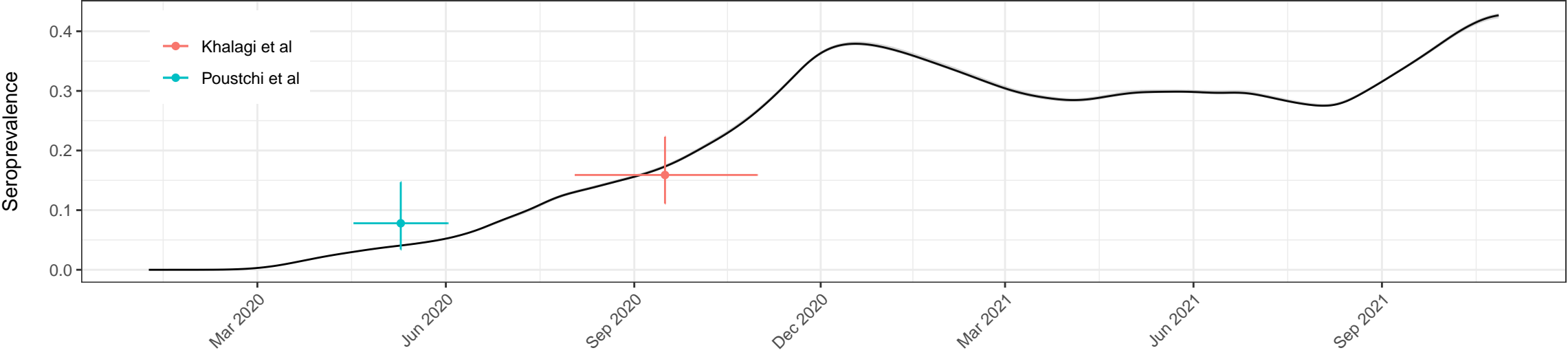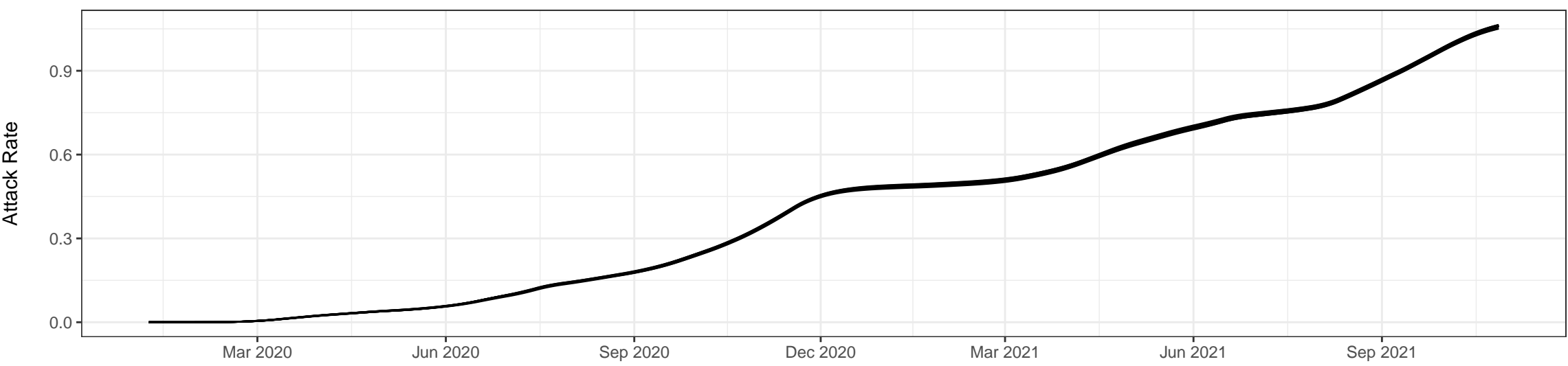

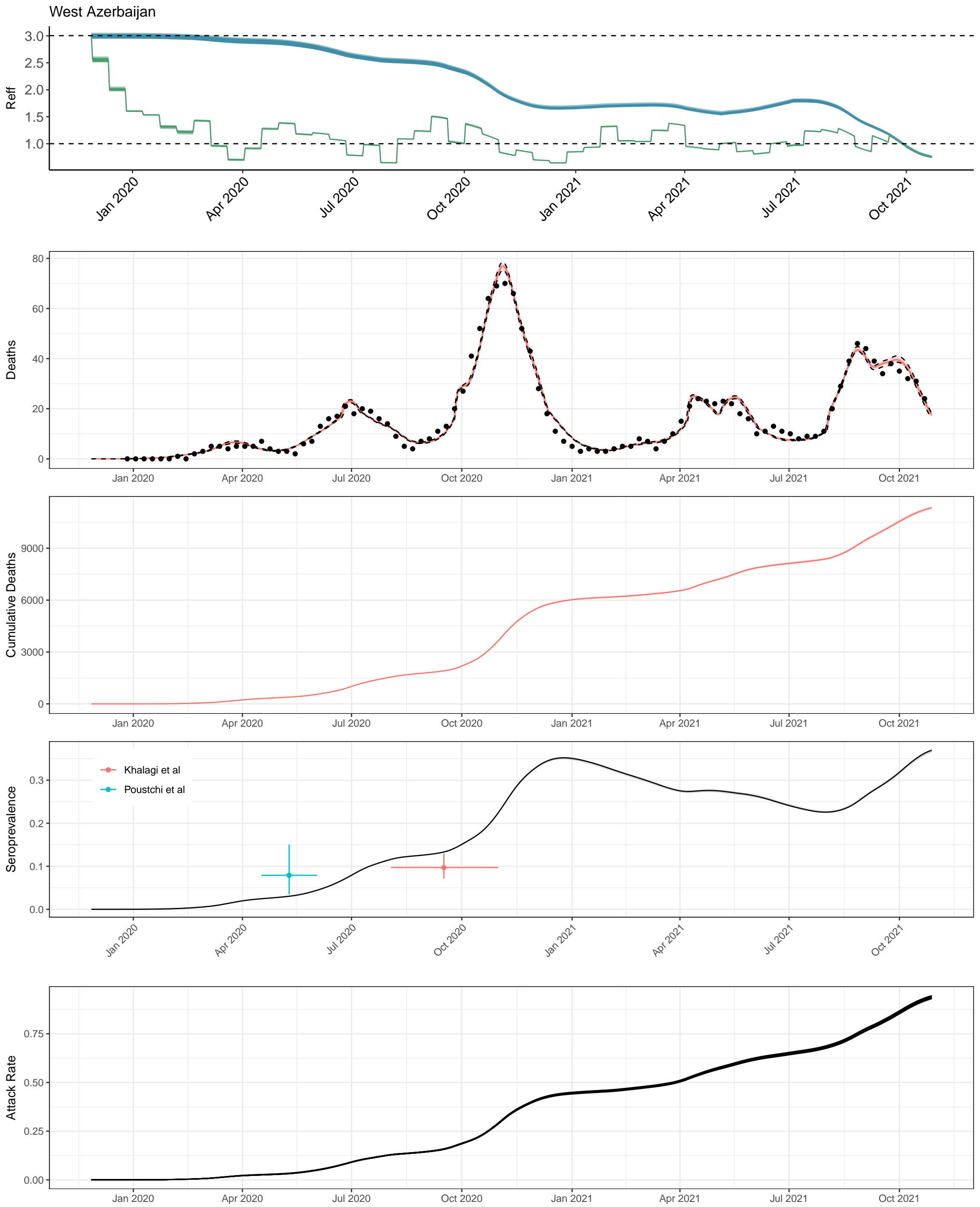

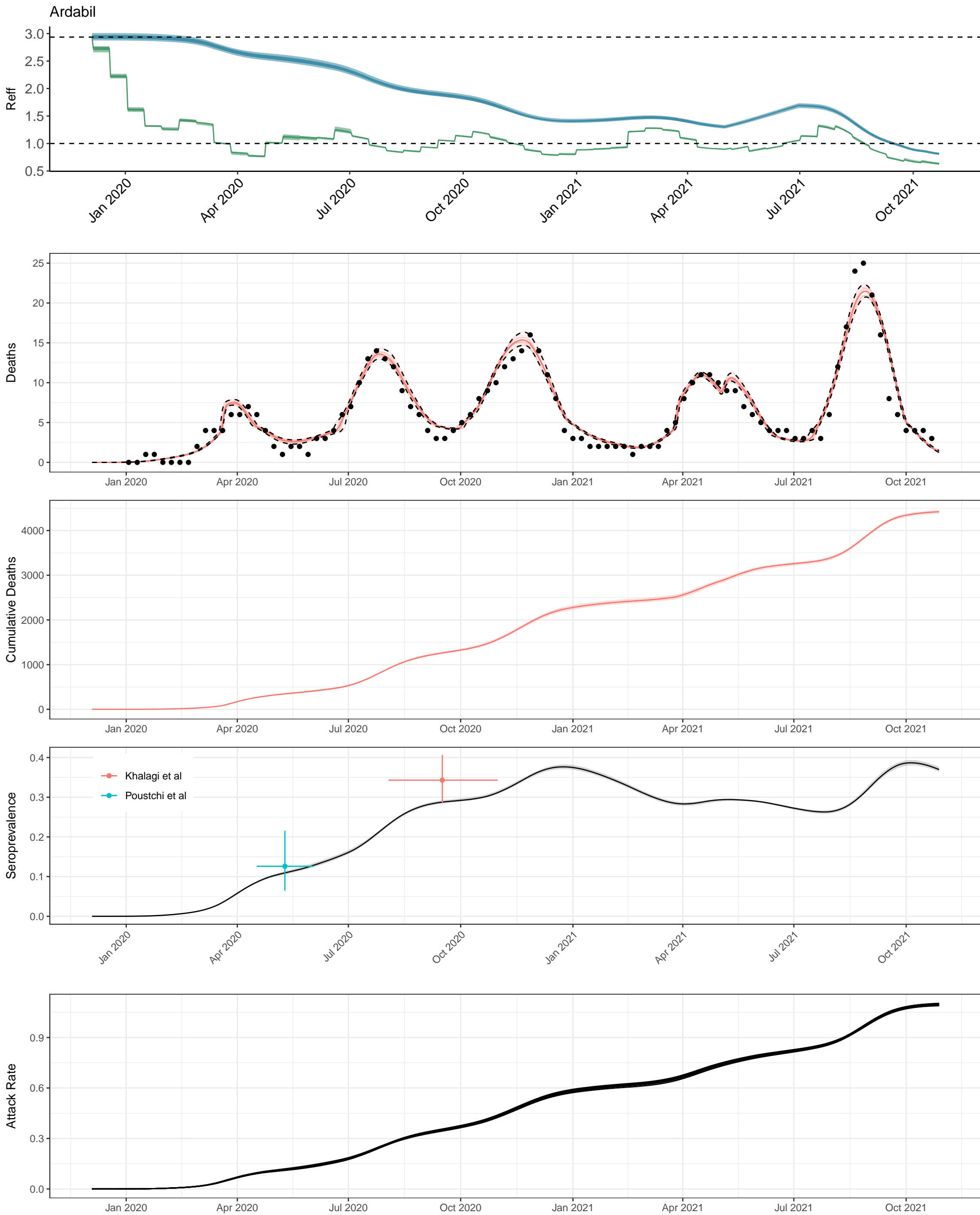

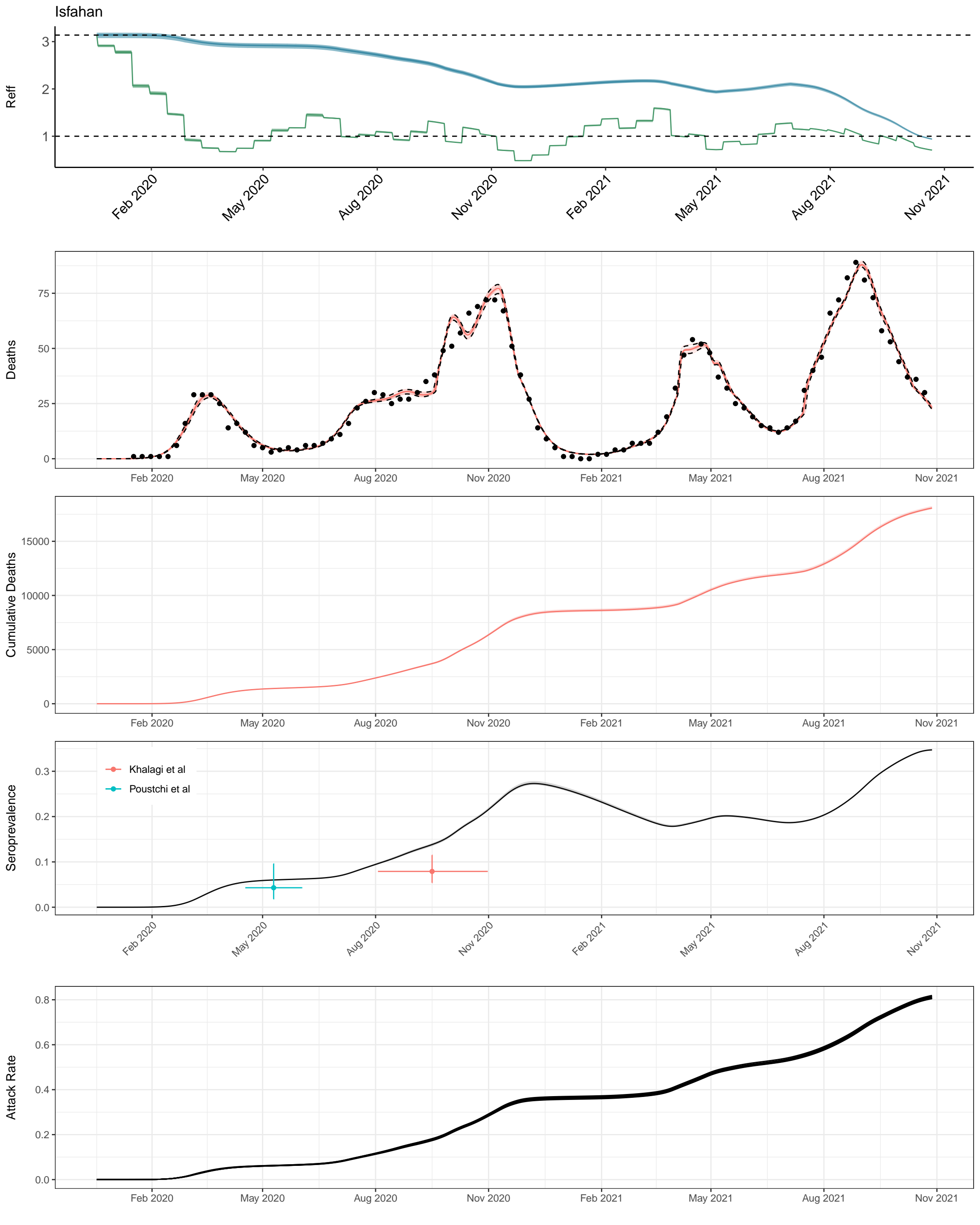

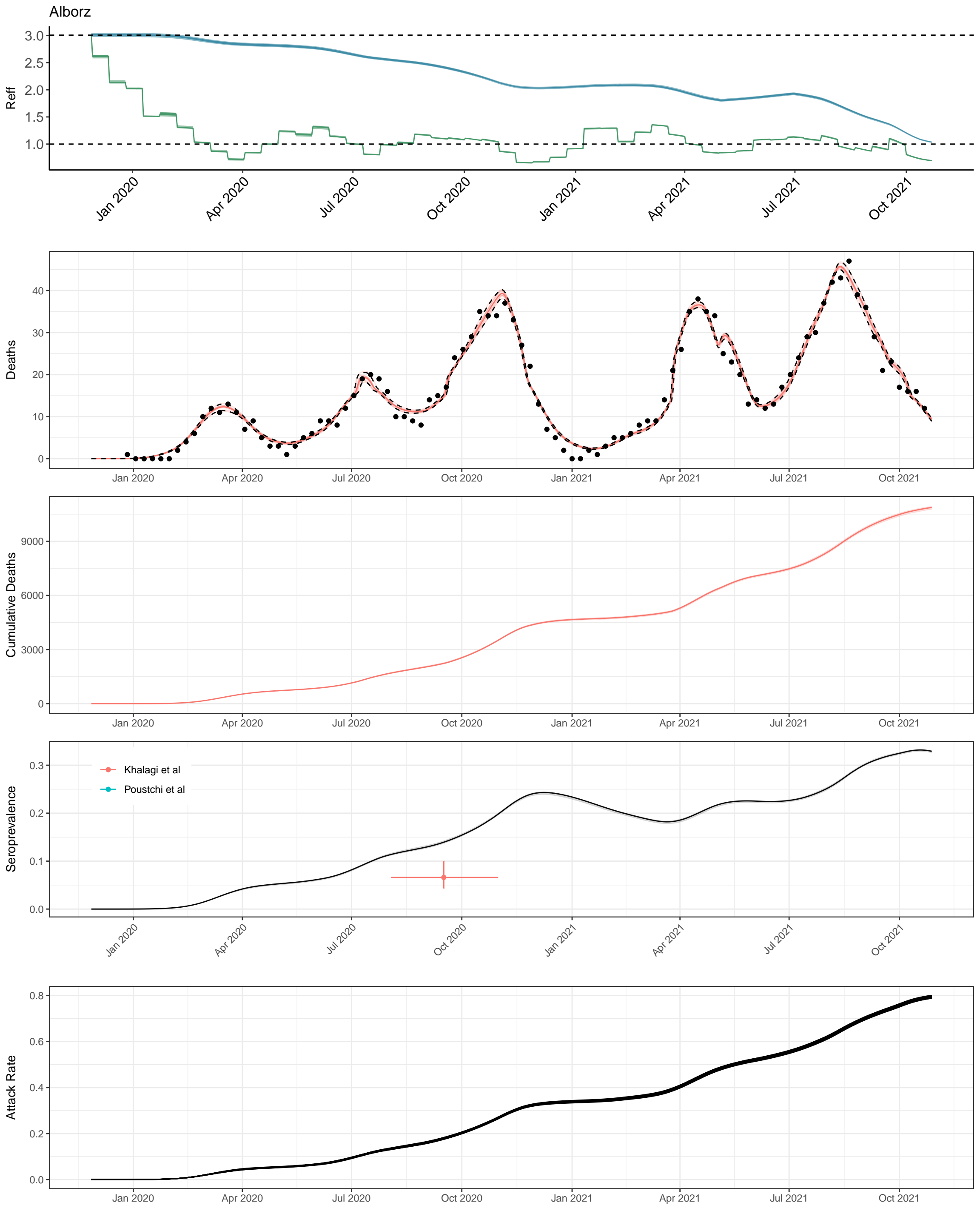

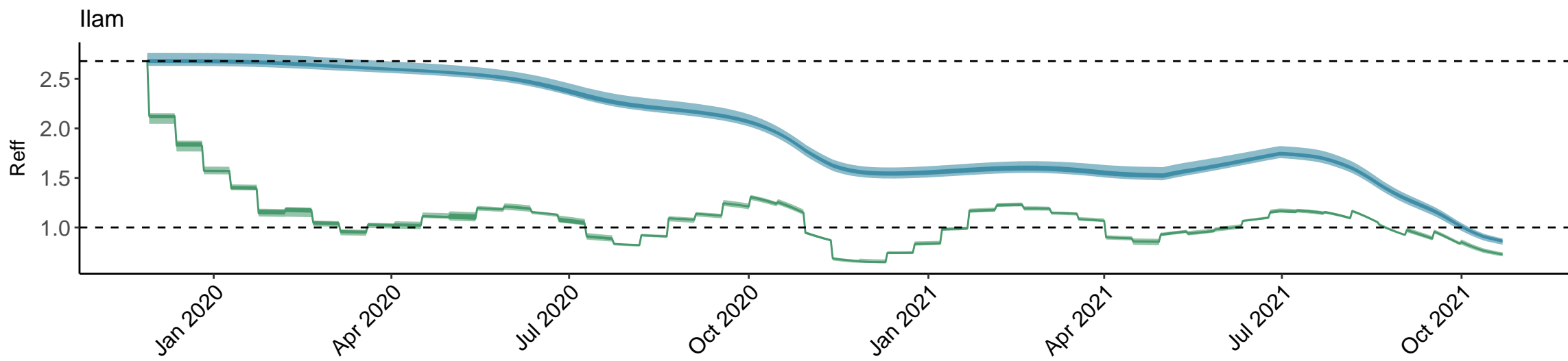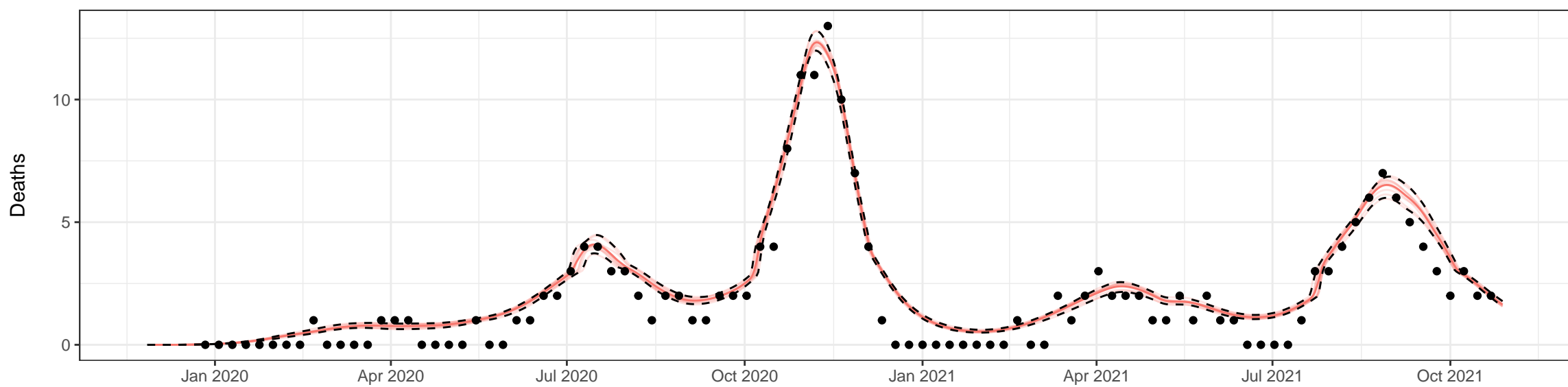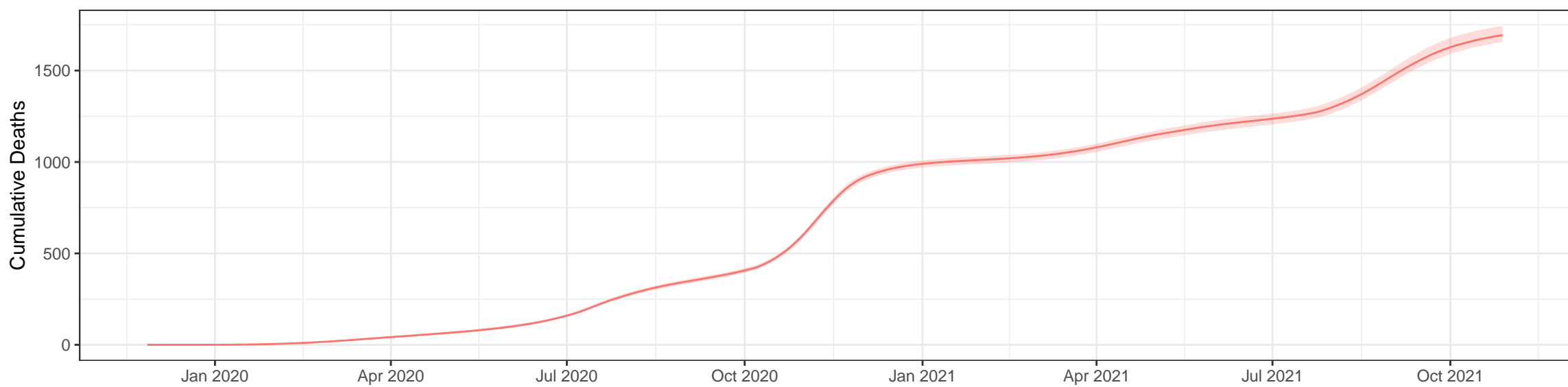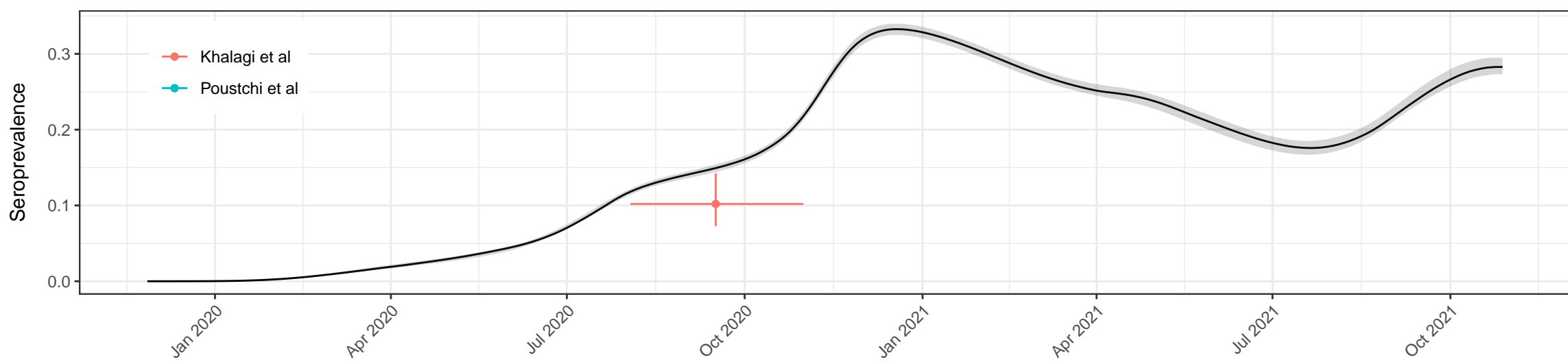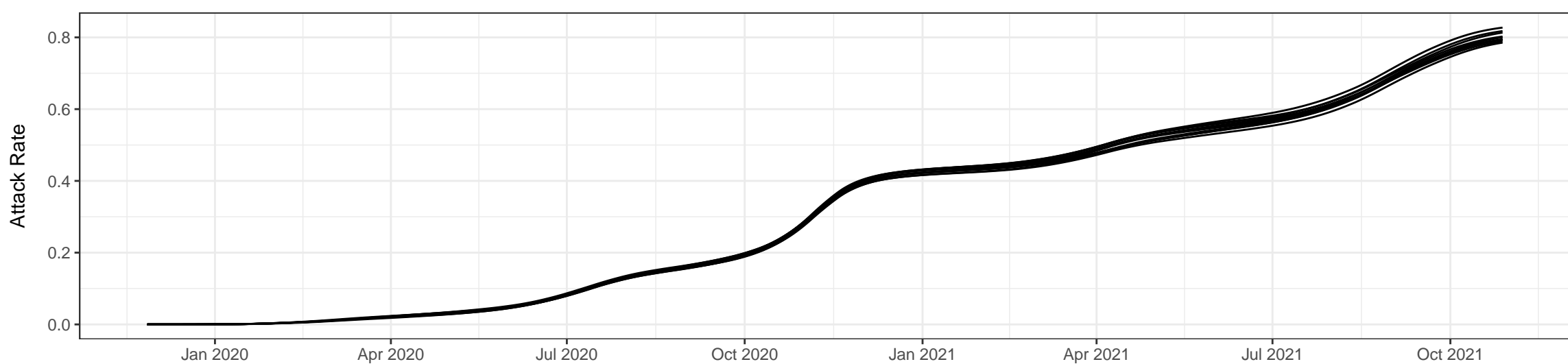

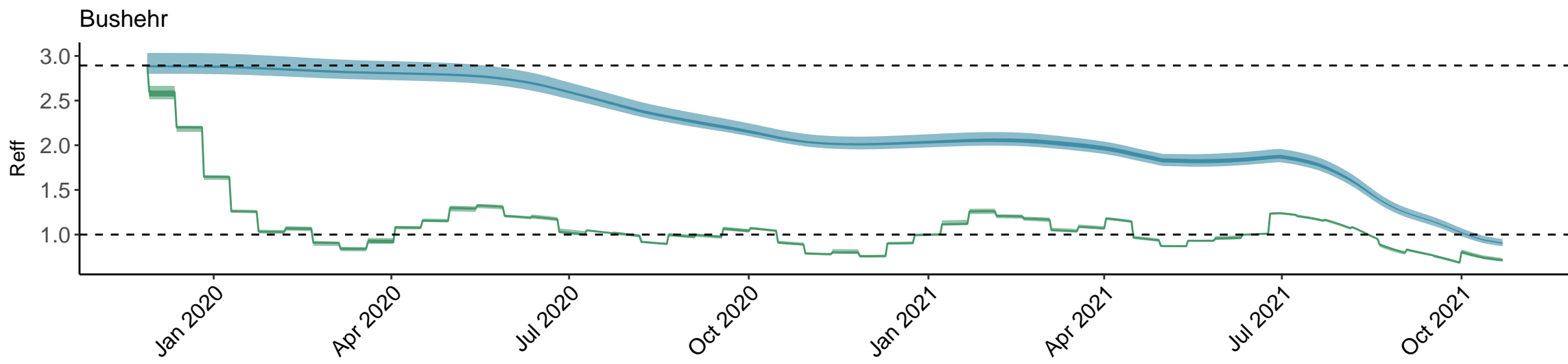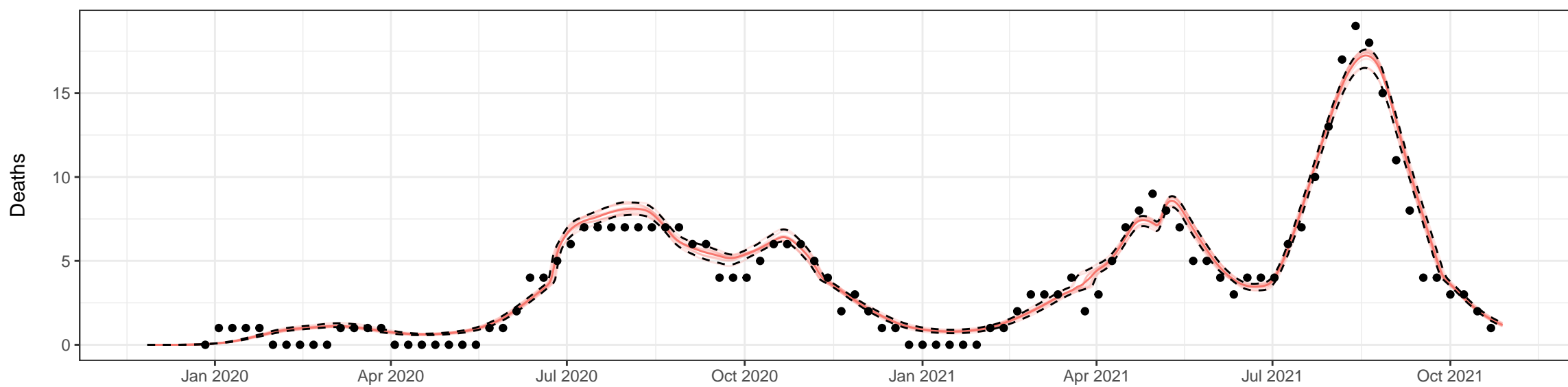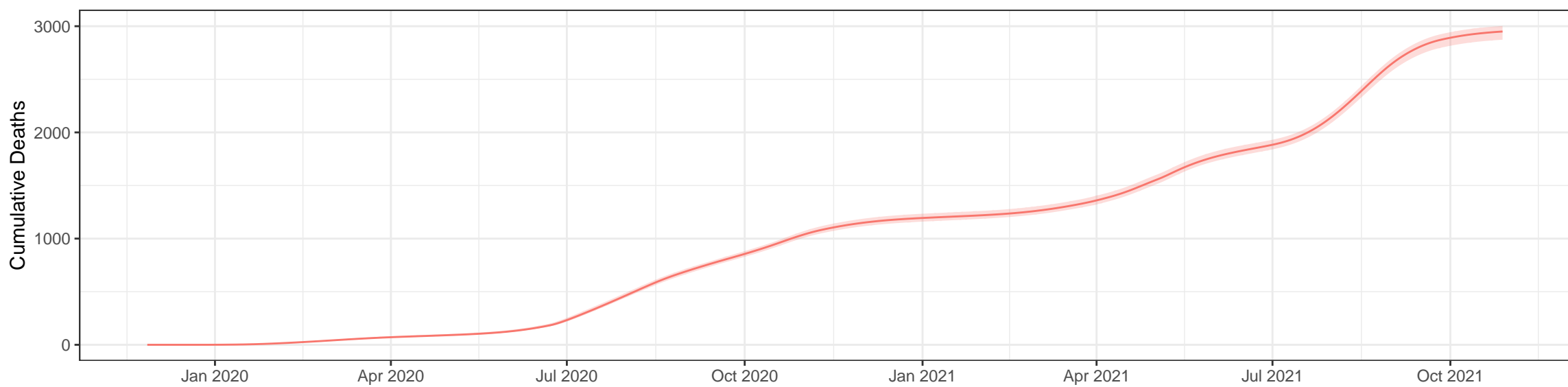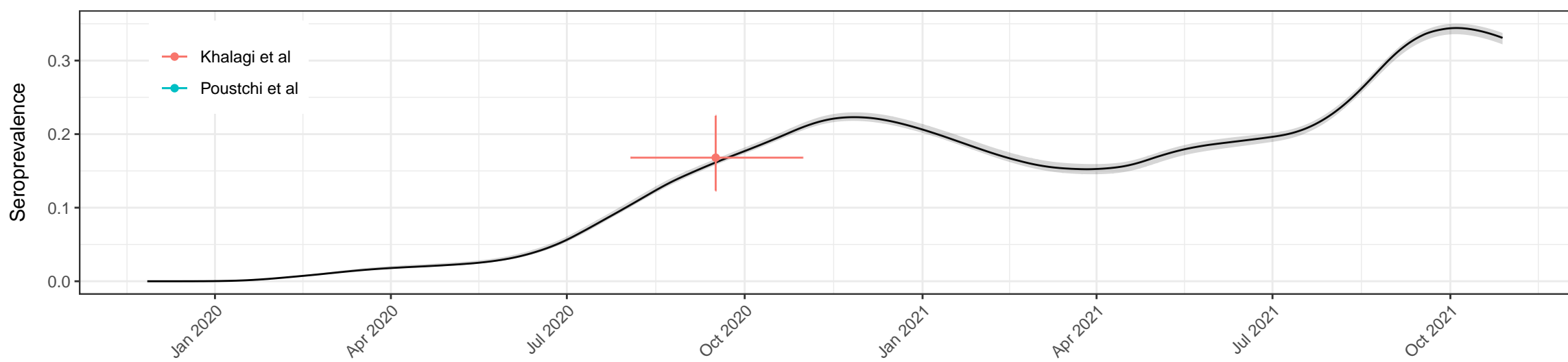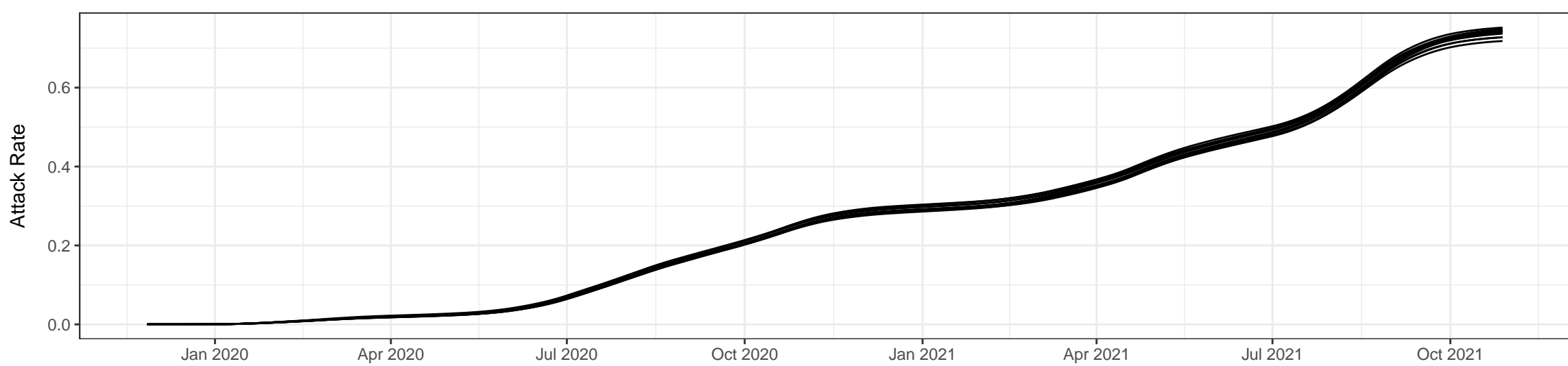

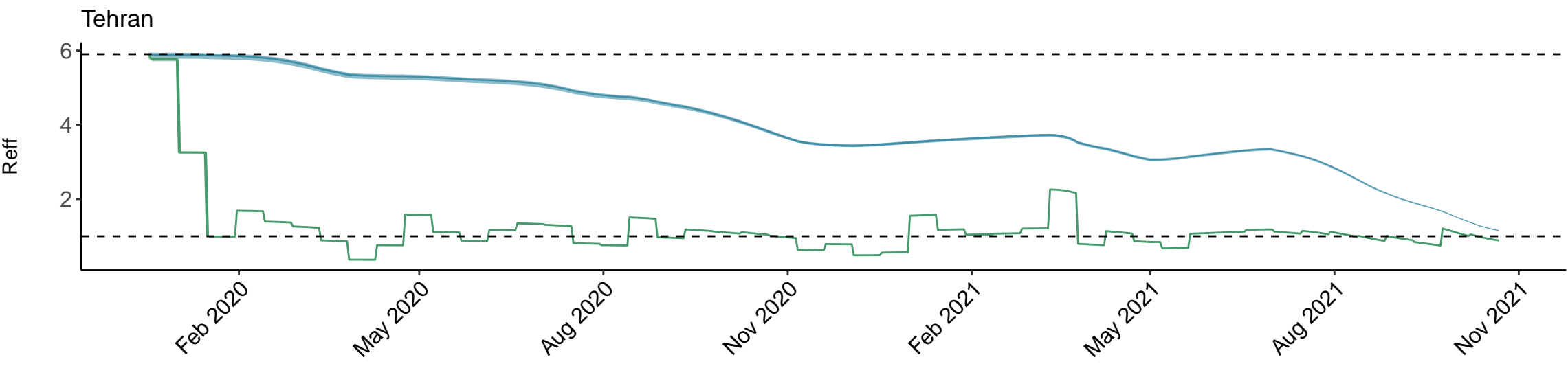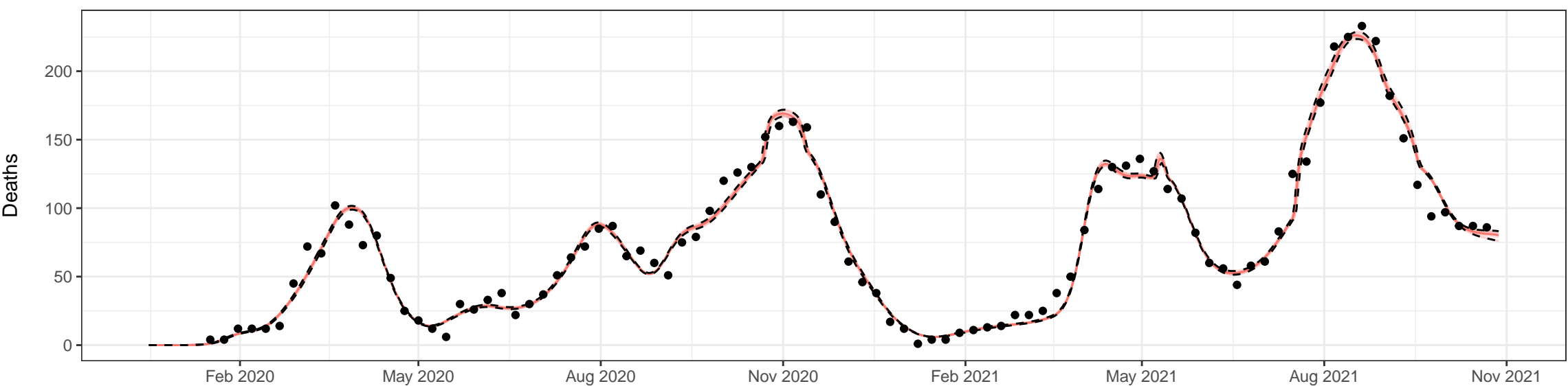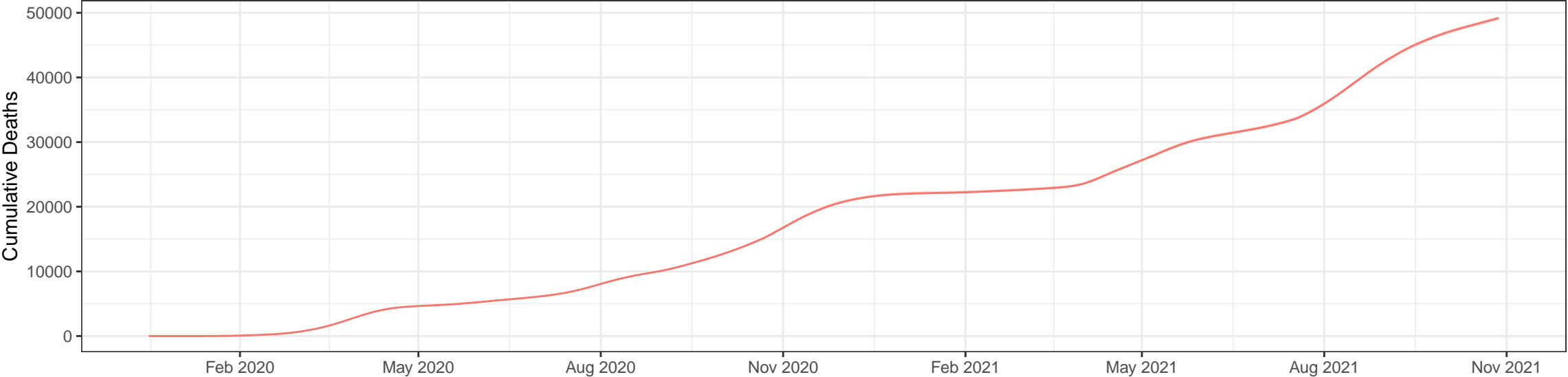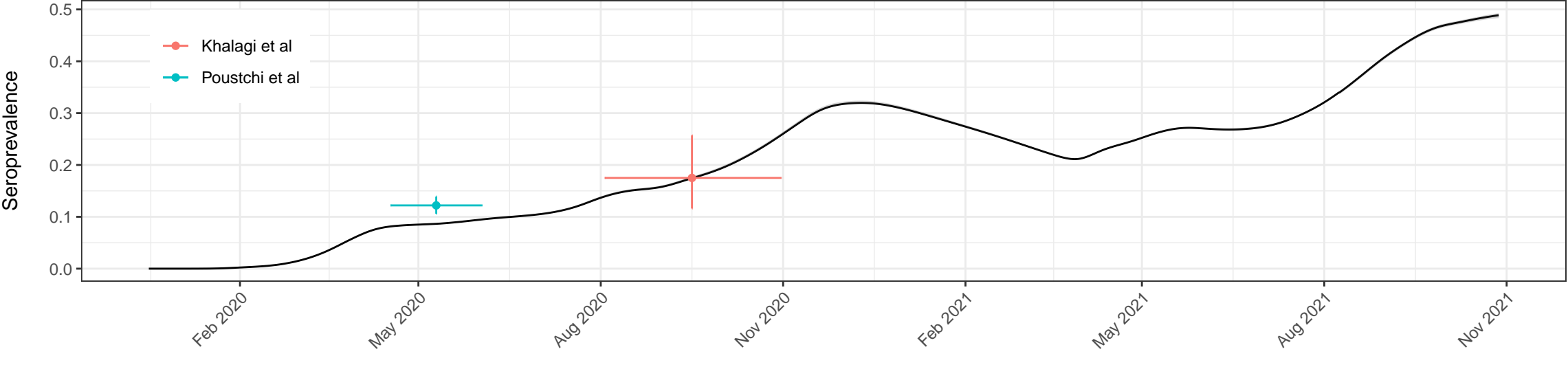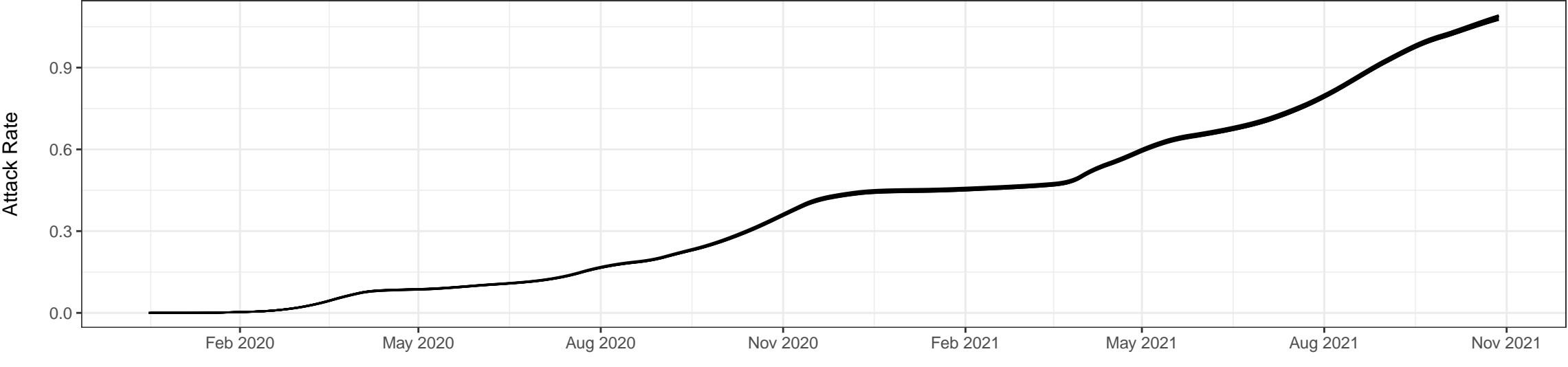

Chahar Mahaal and Bakhtiari

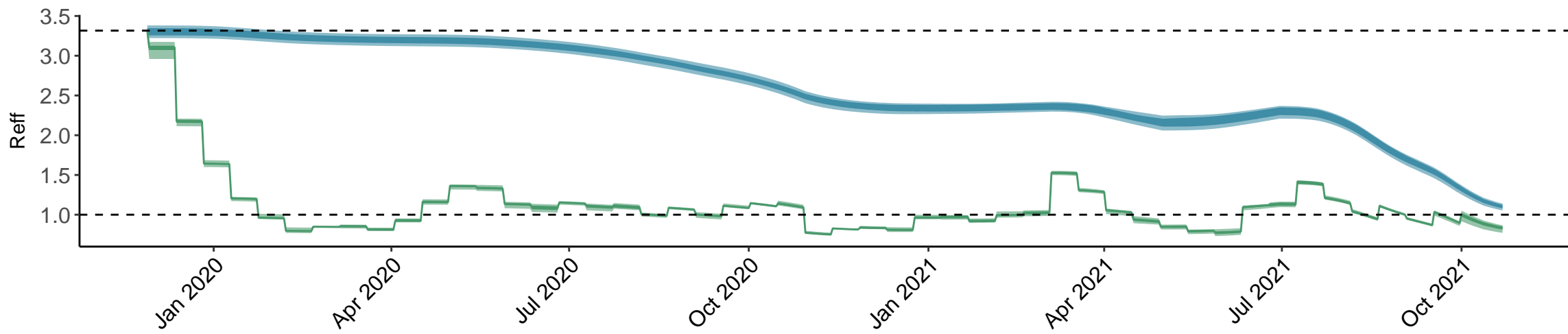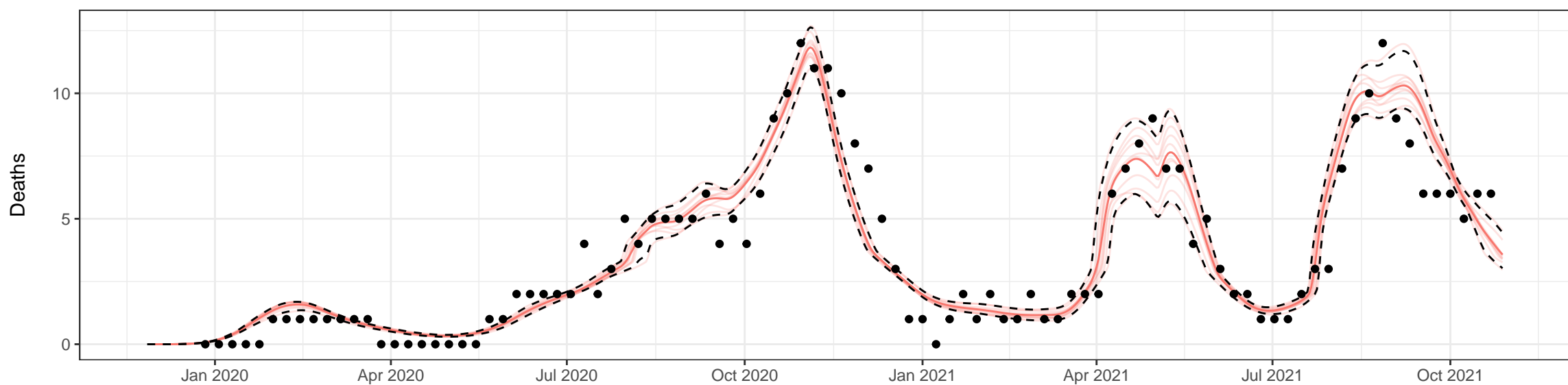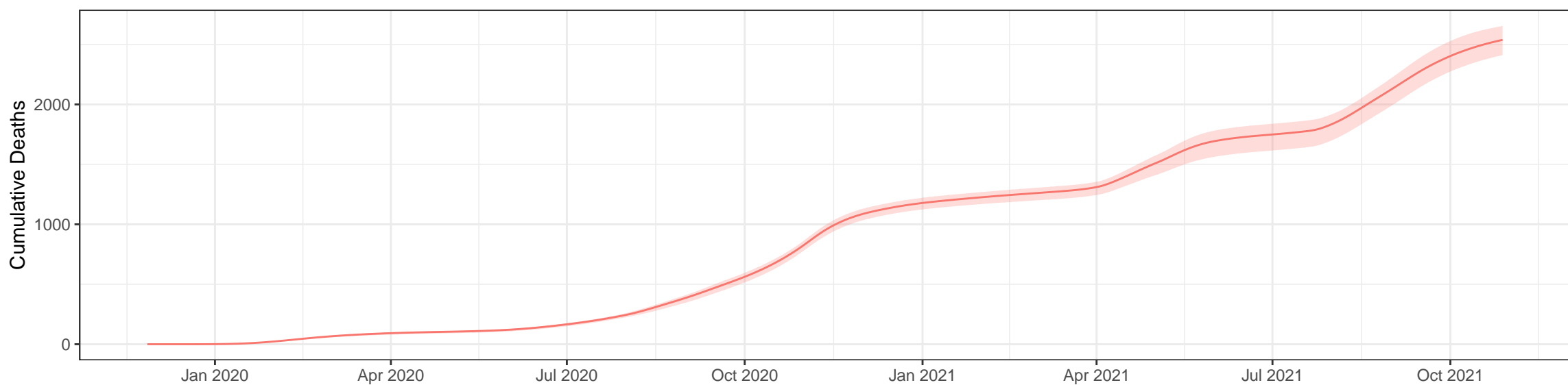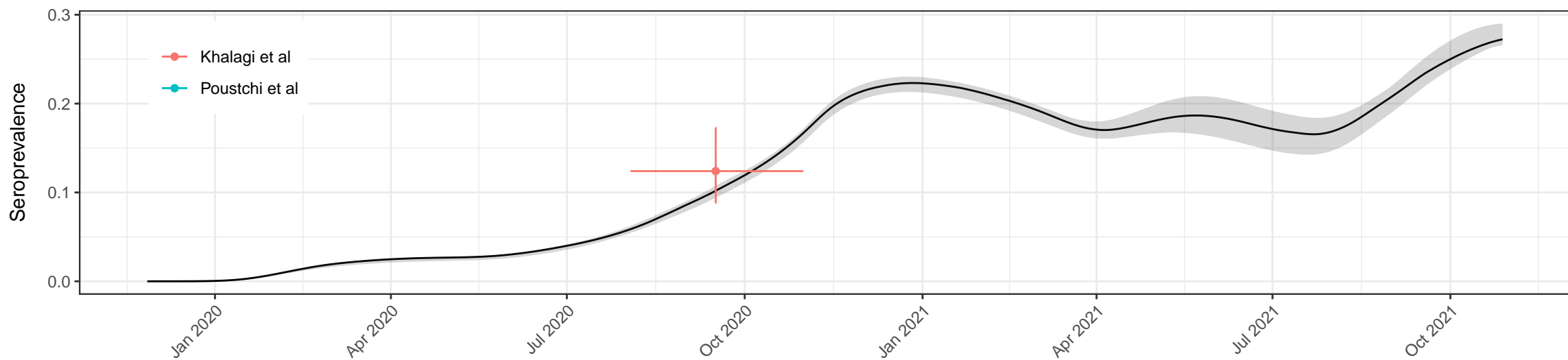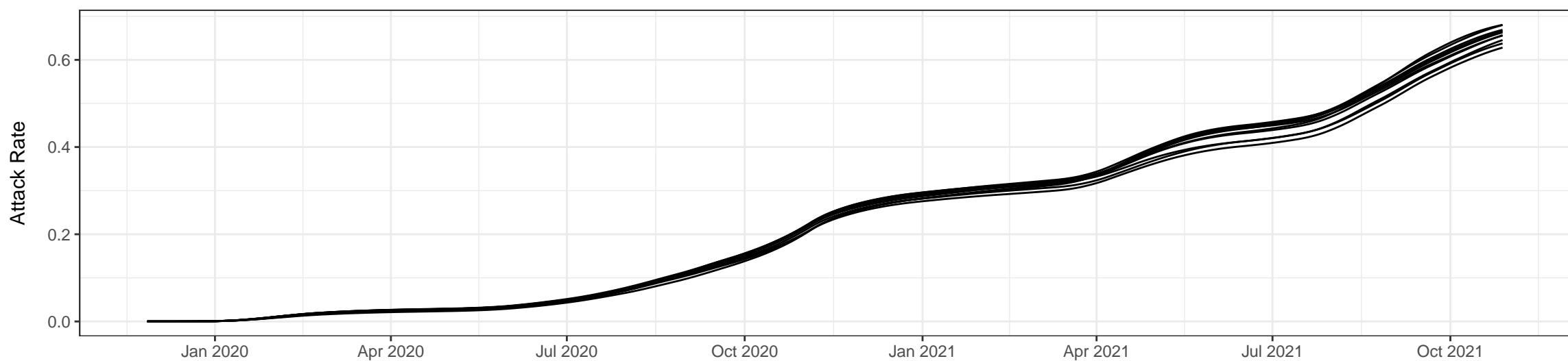

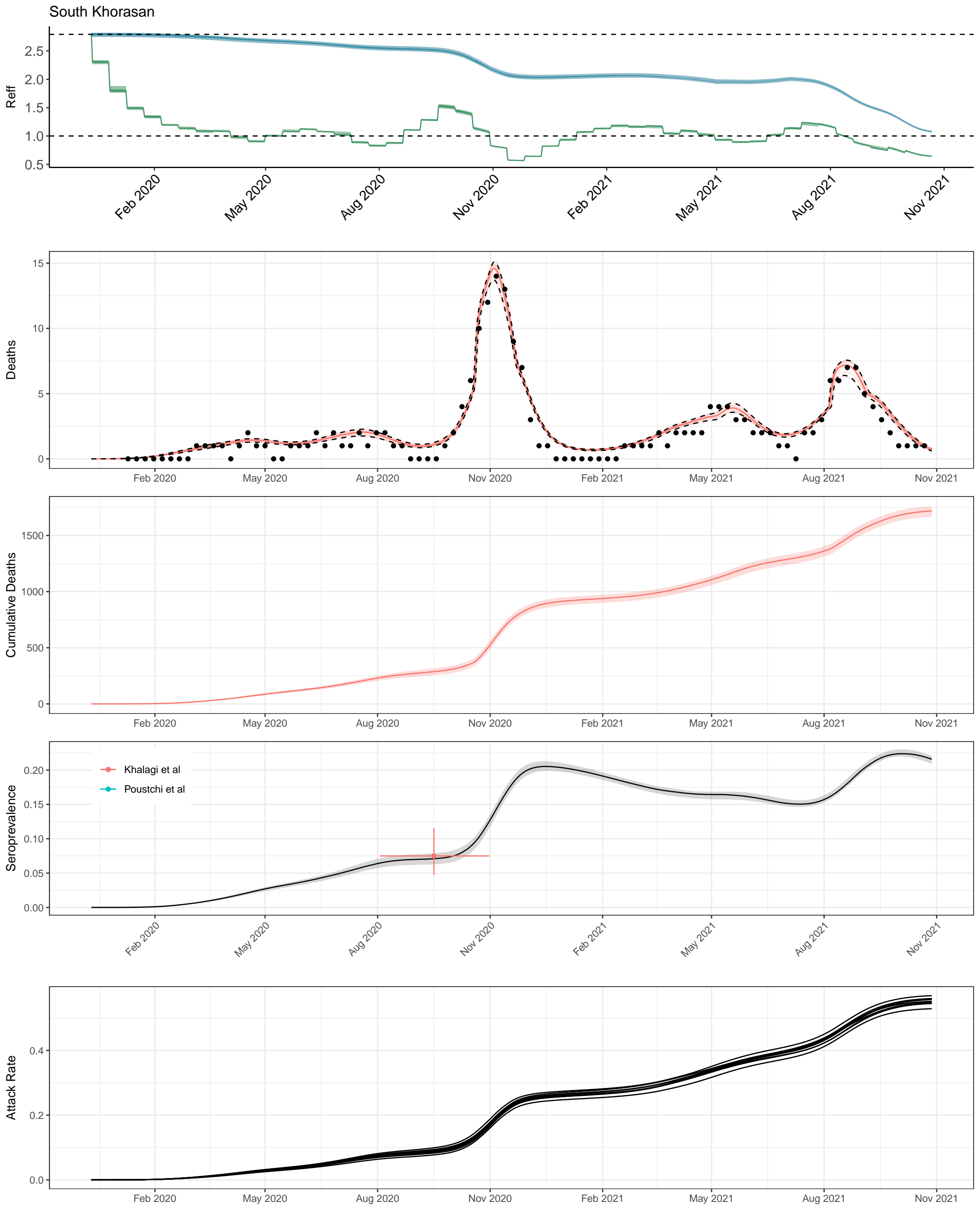

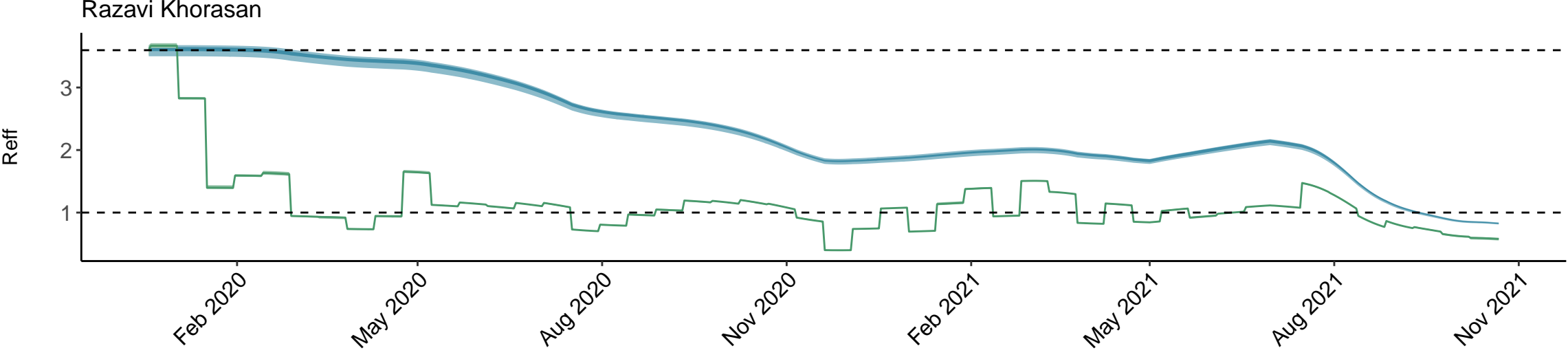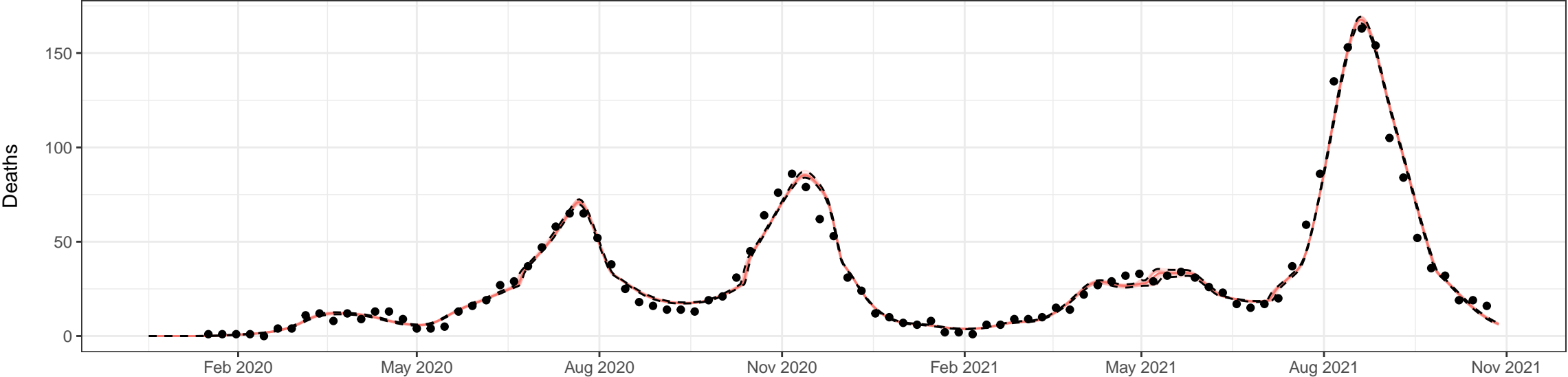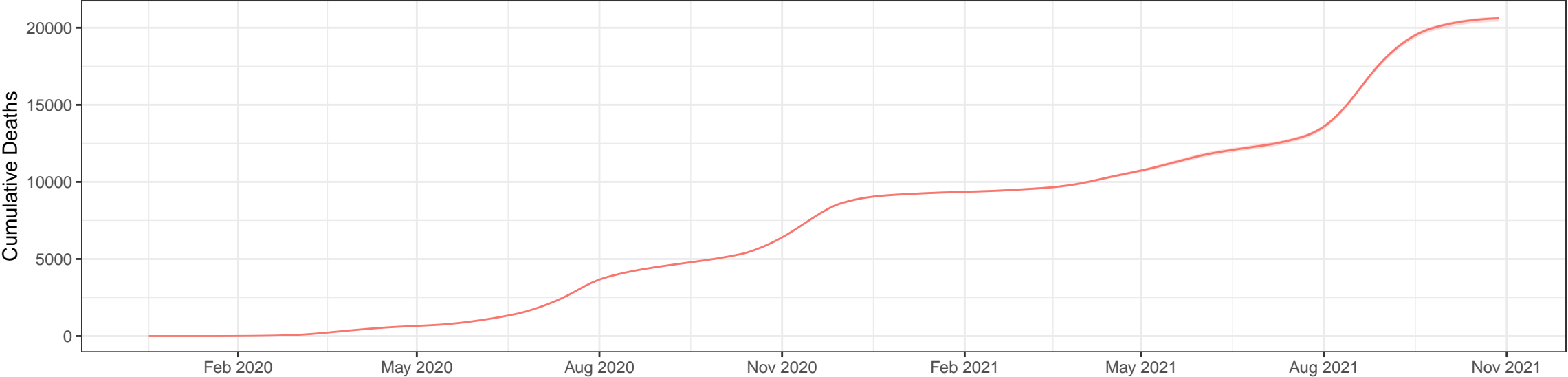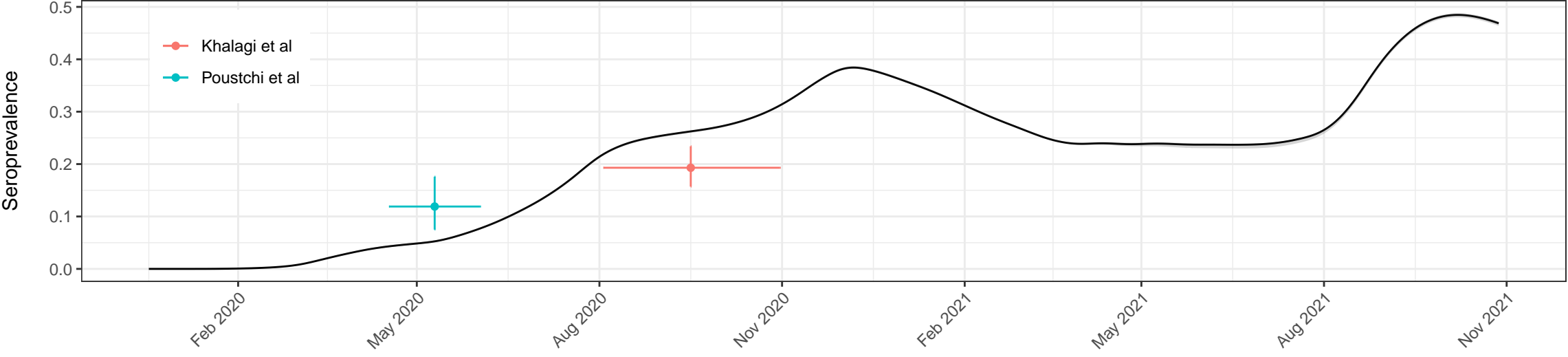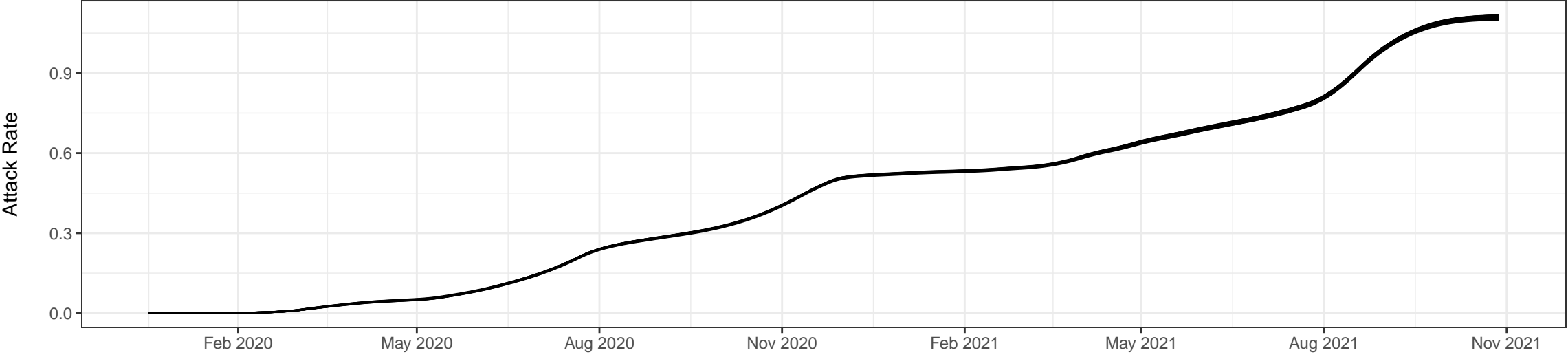

North Khorasan

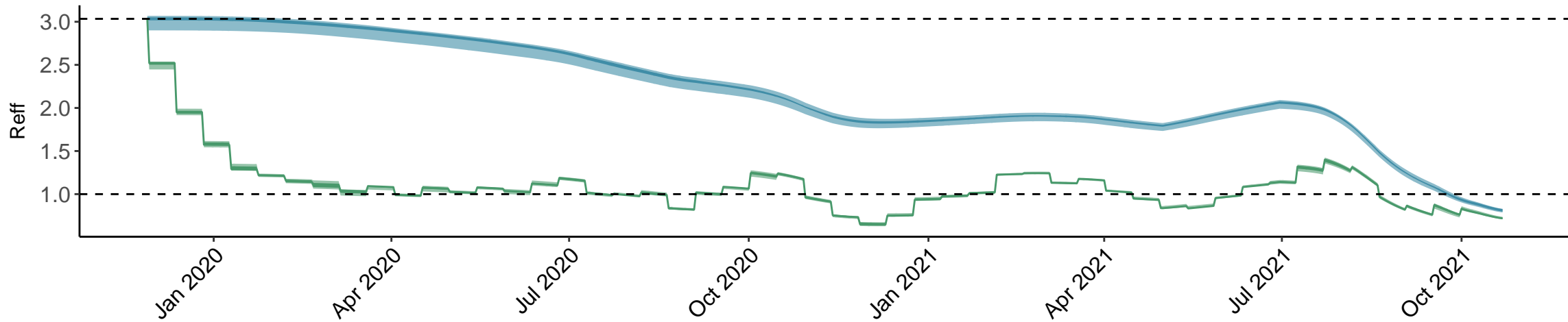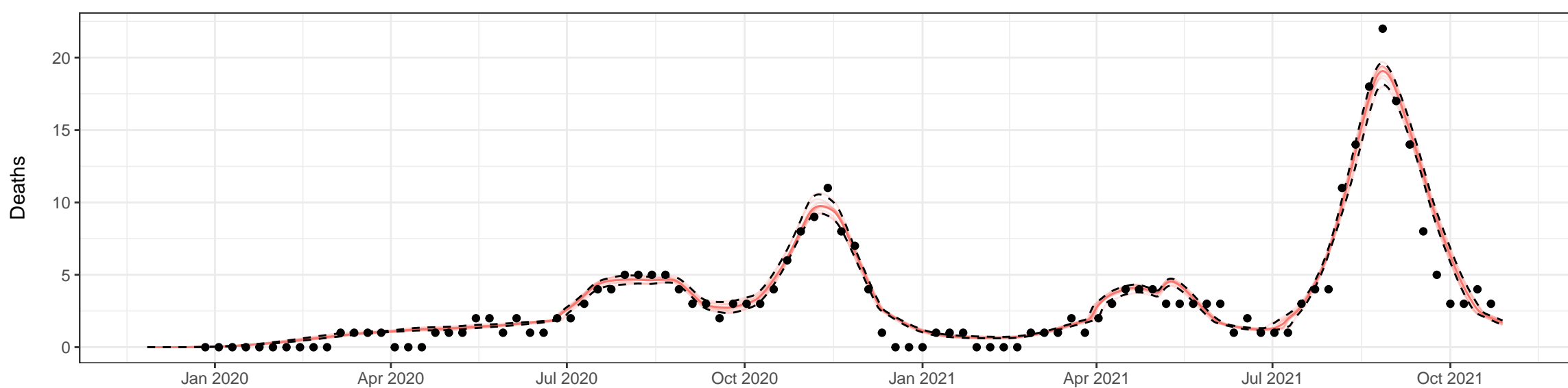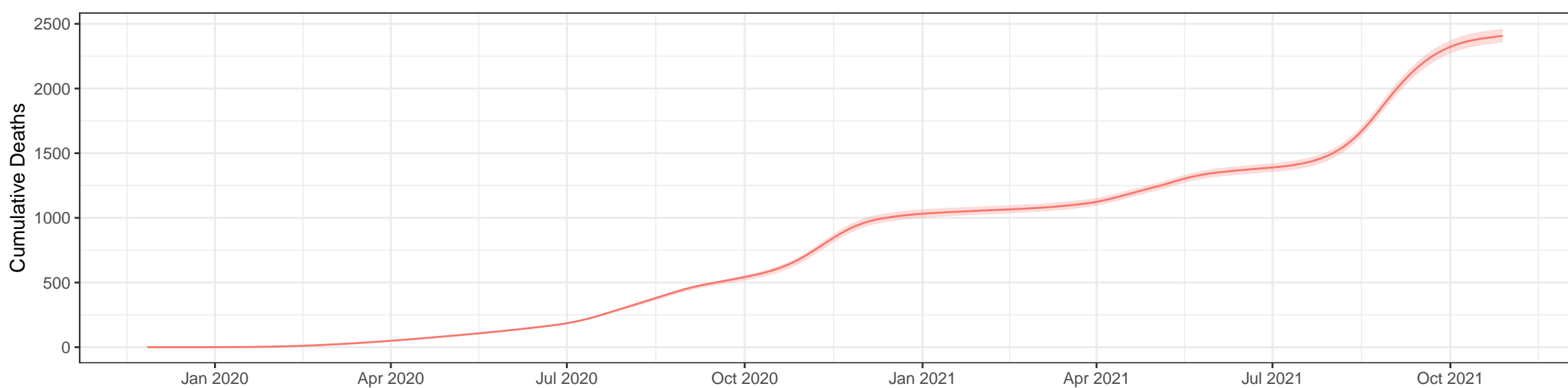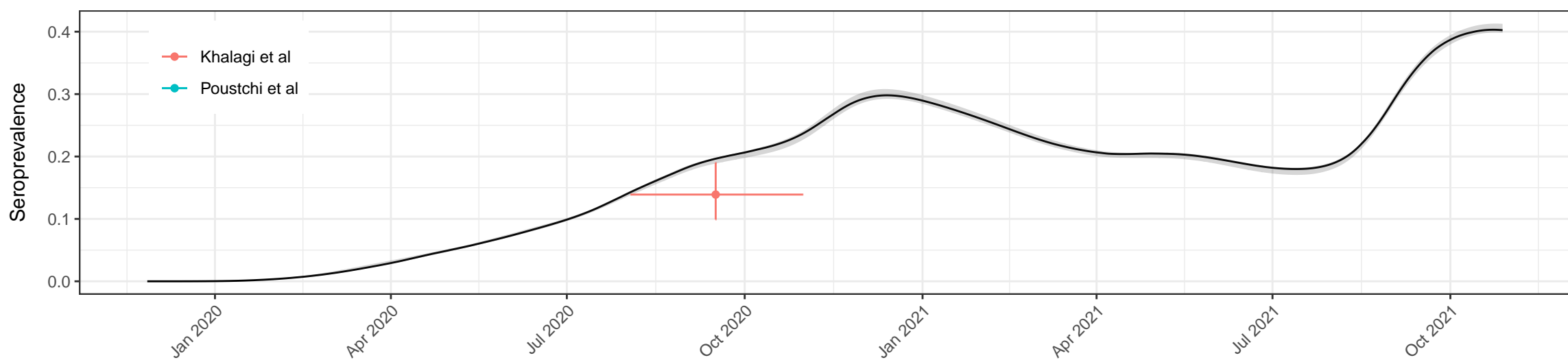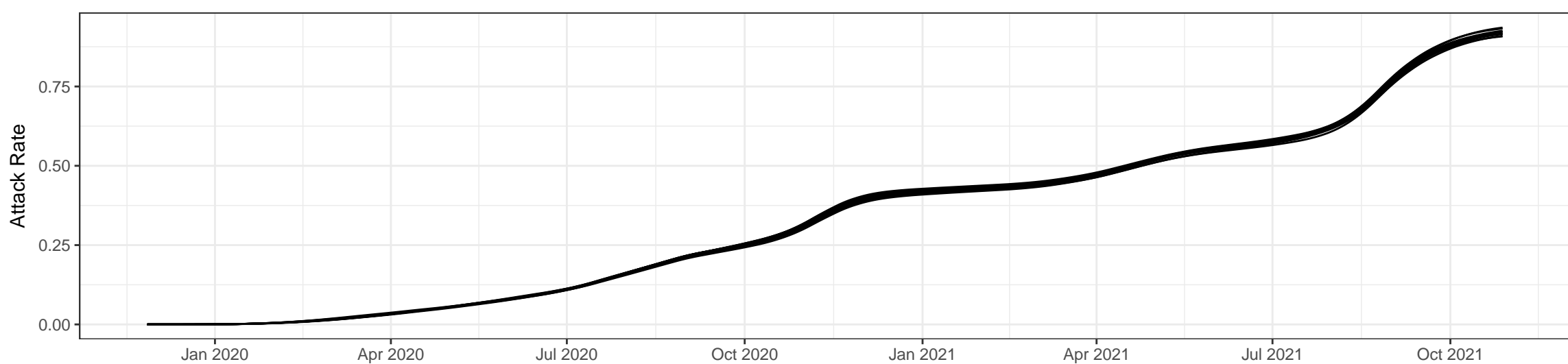

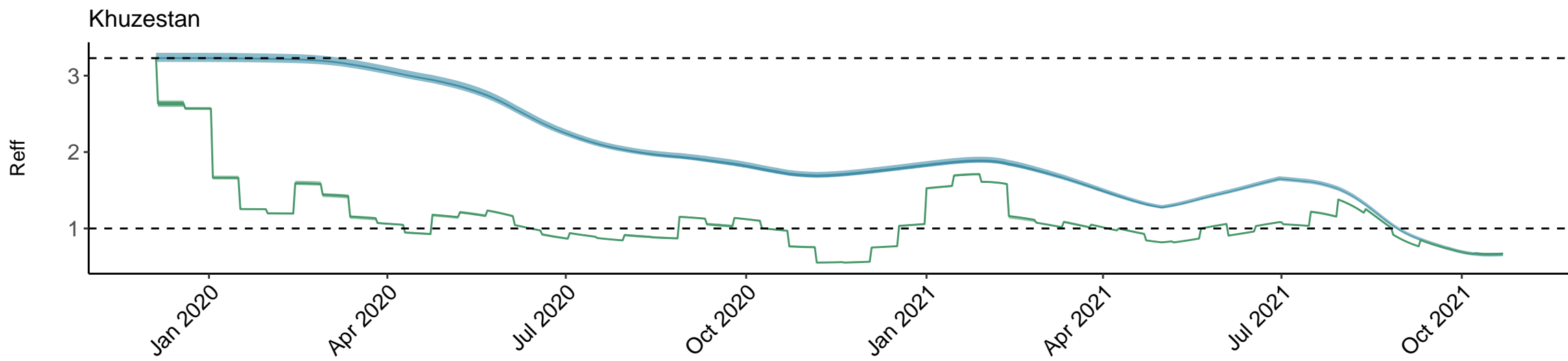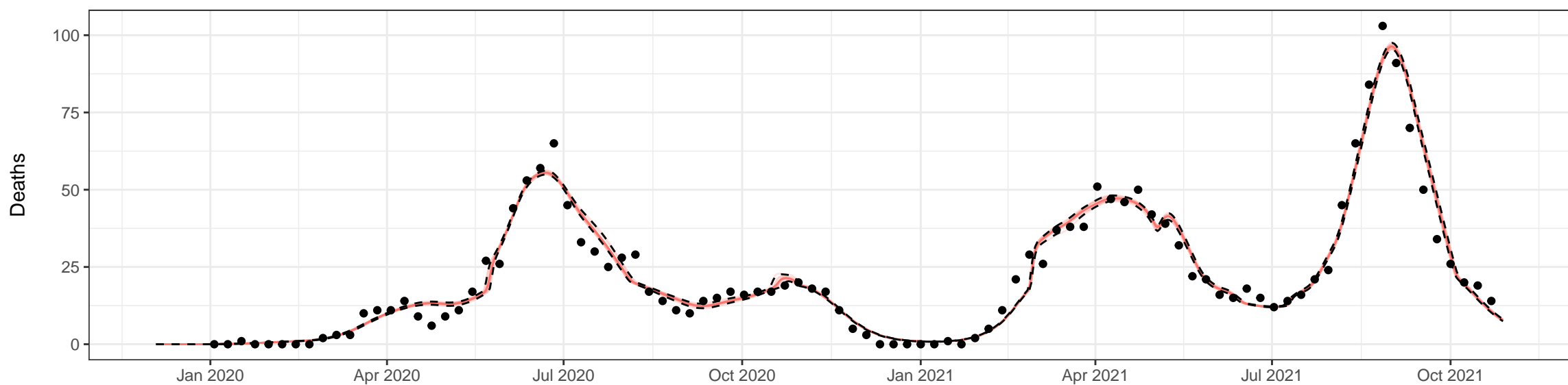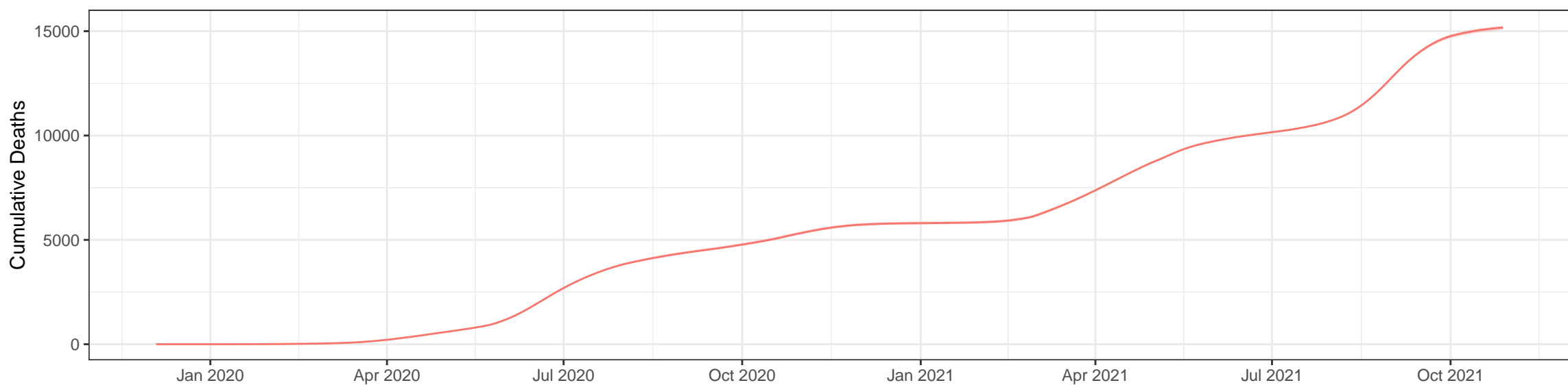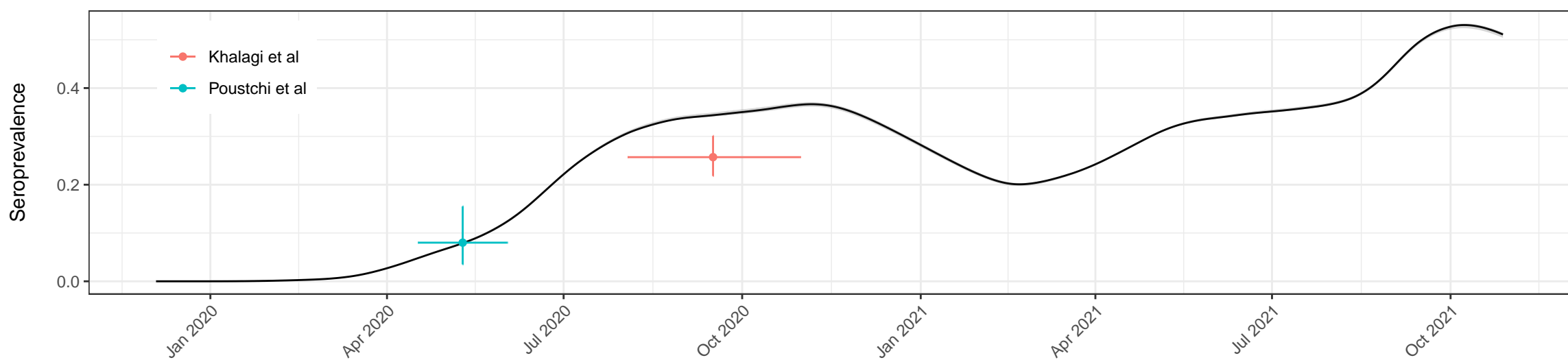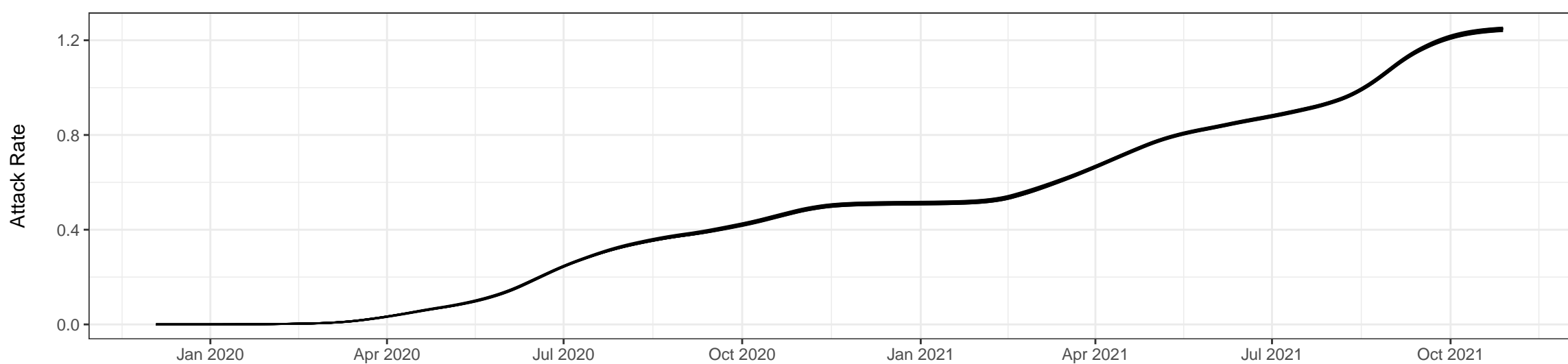

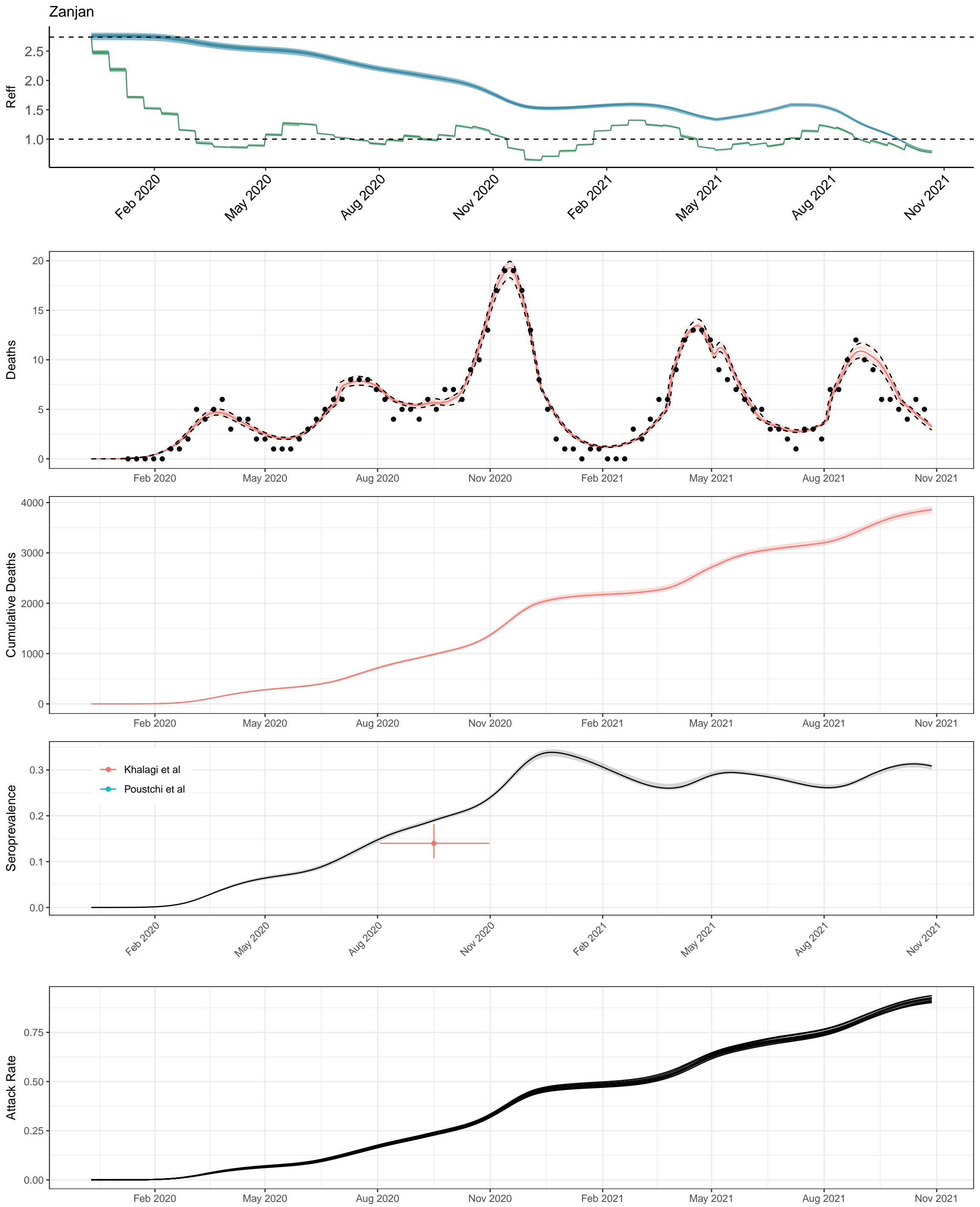

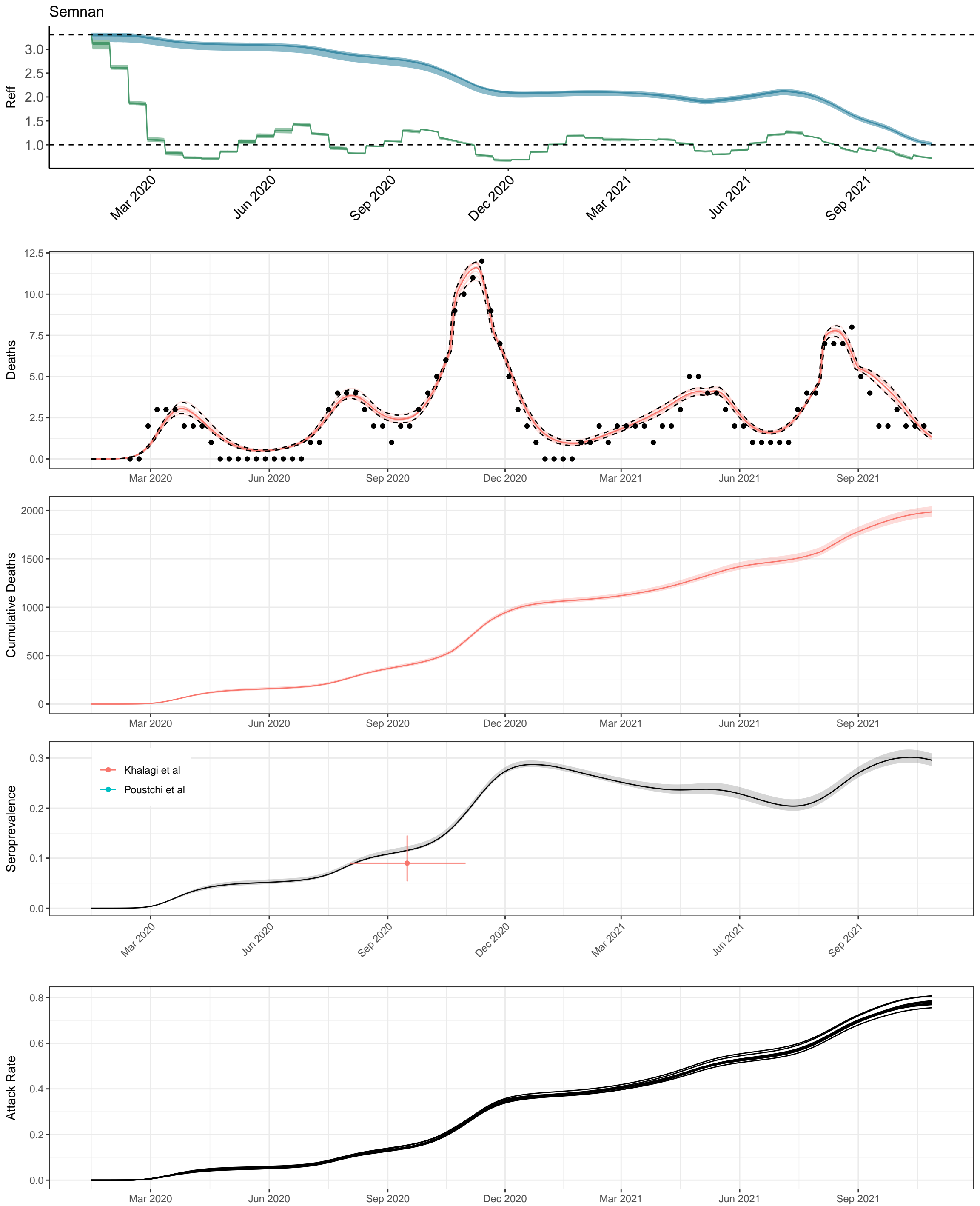

# Sistan and Baluchistan

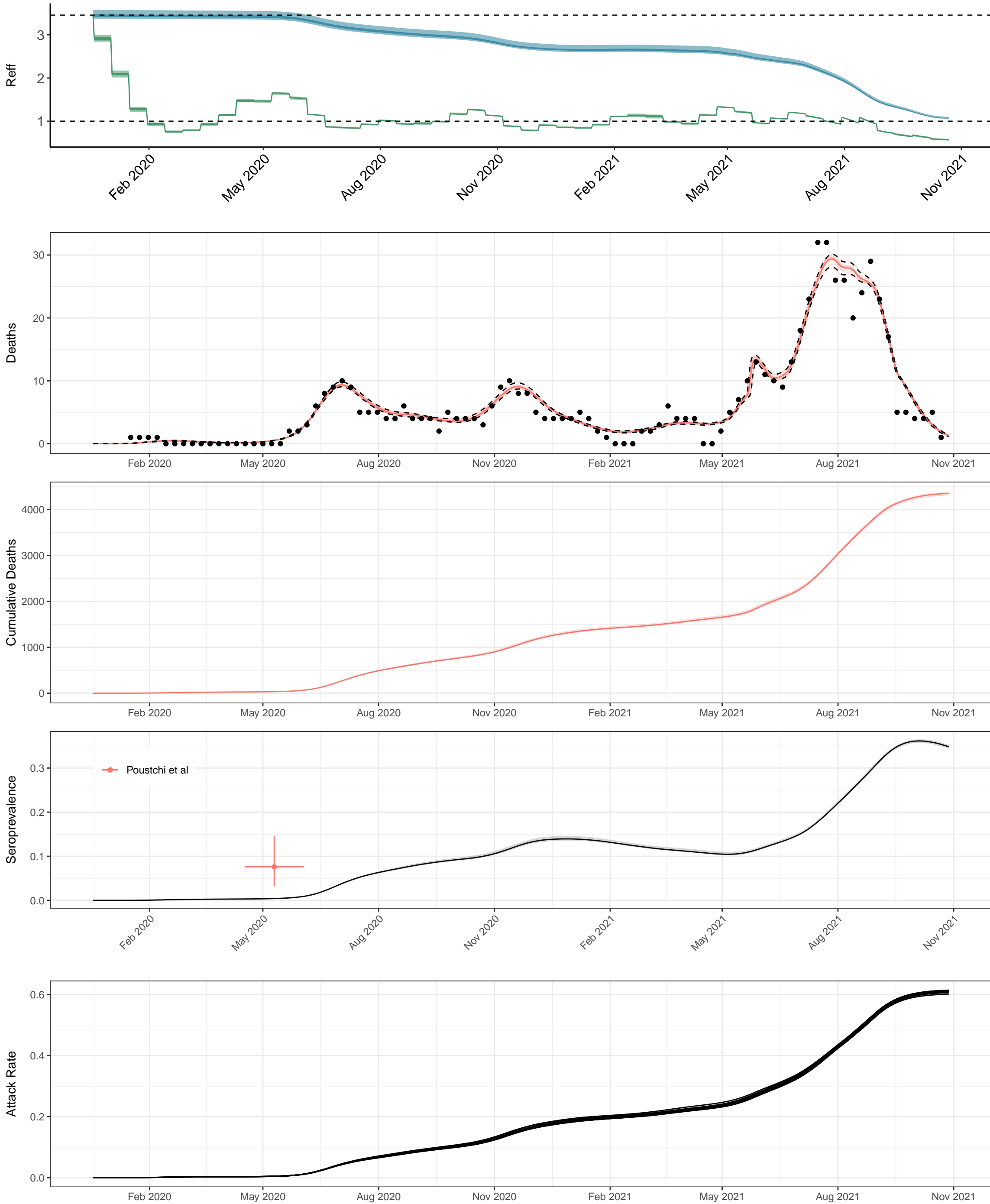

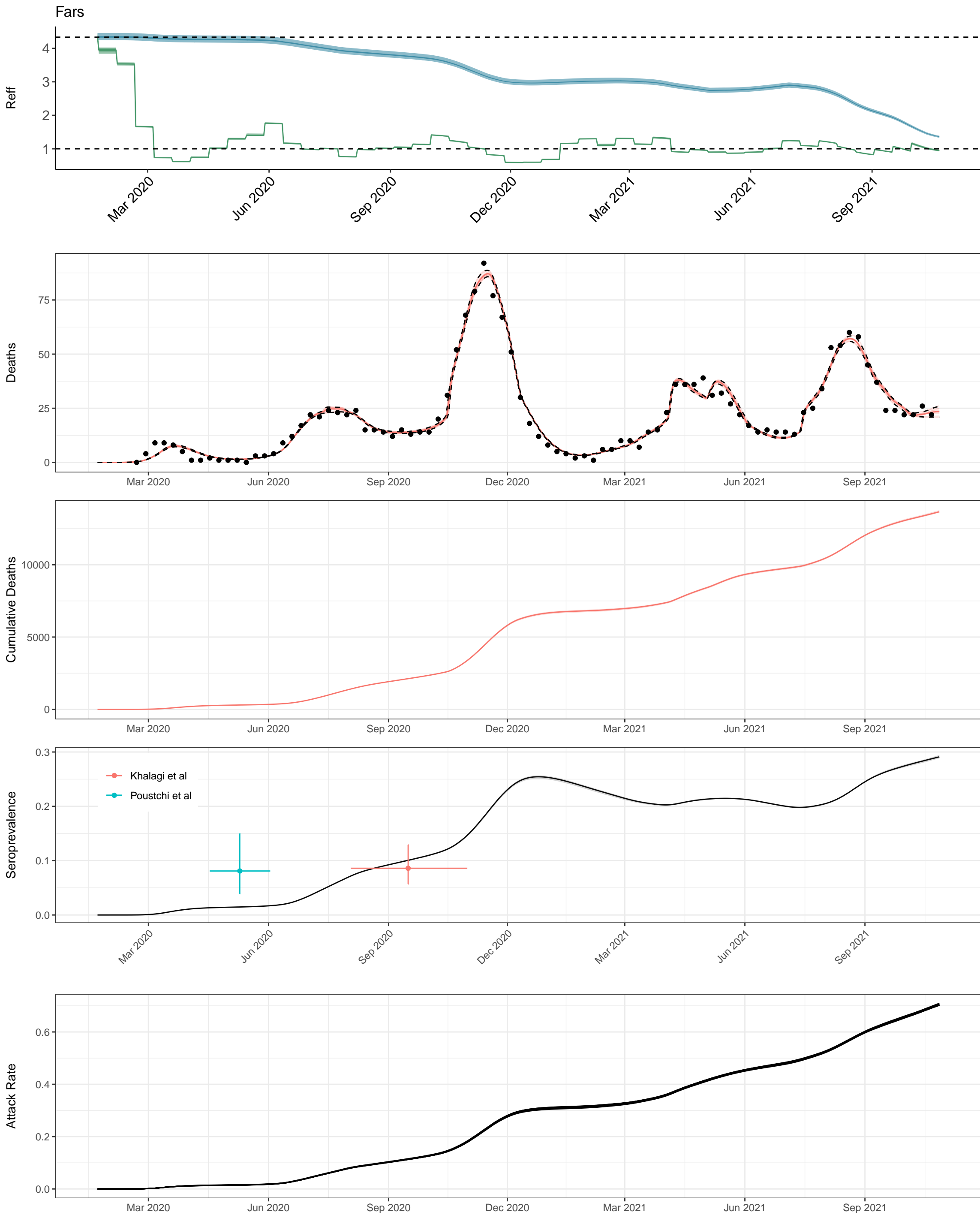

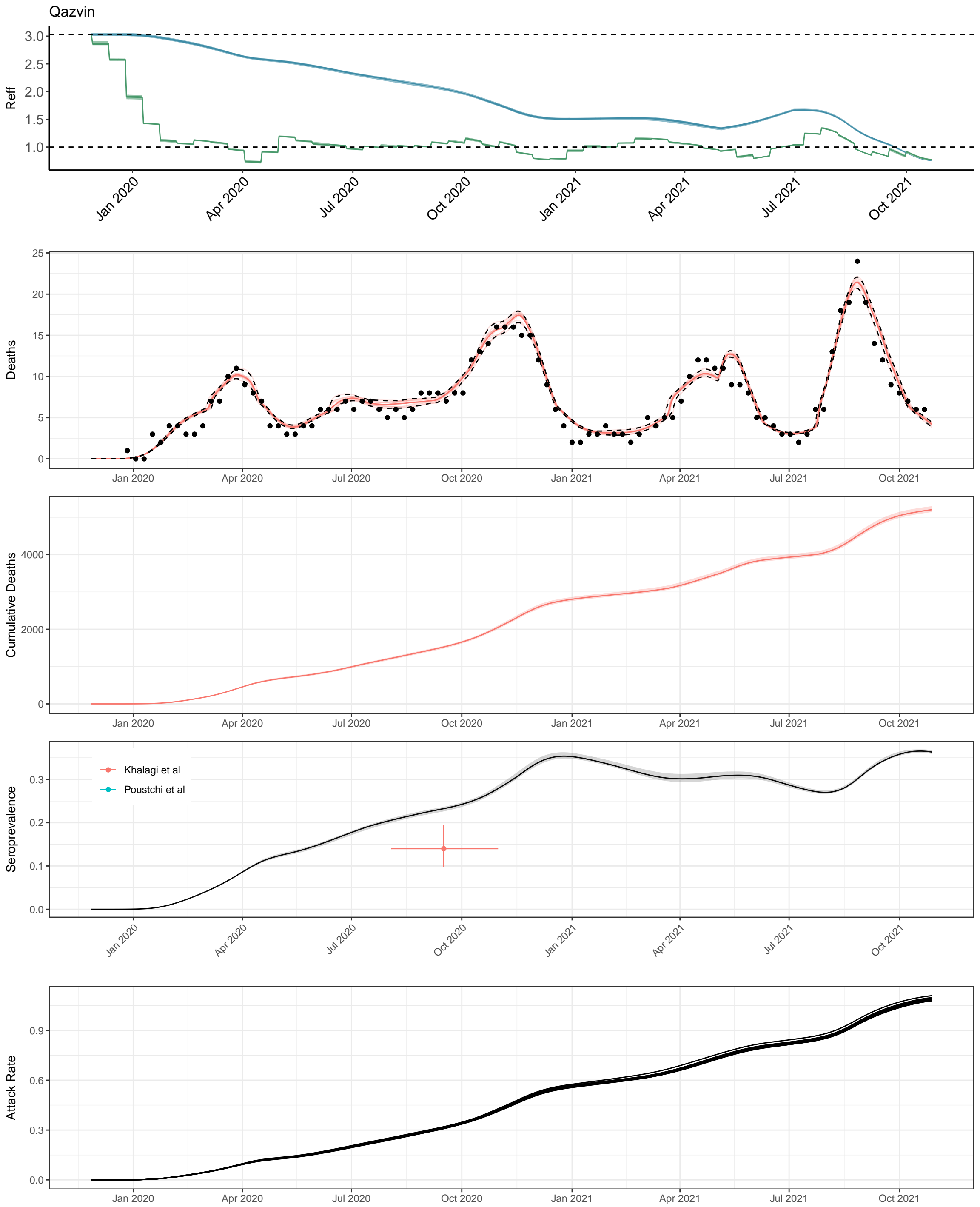

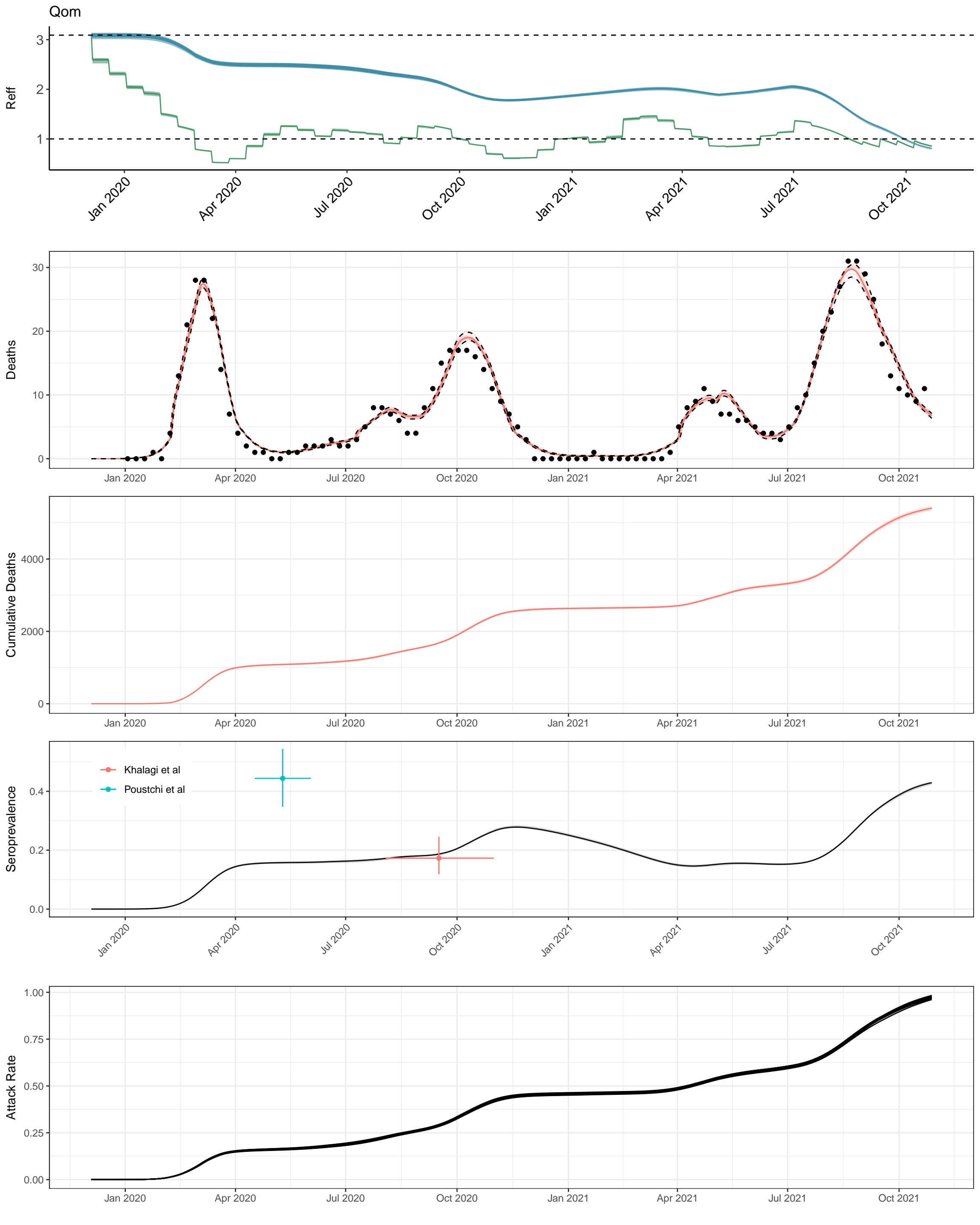

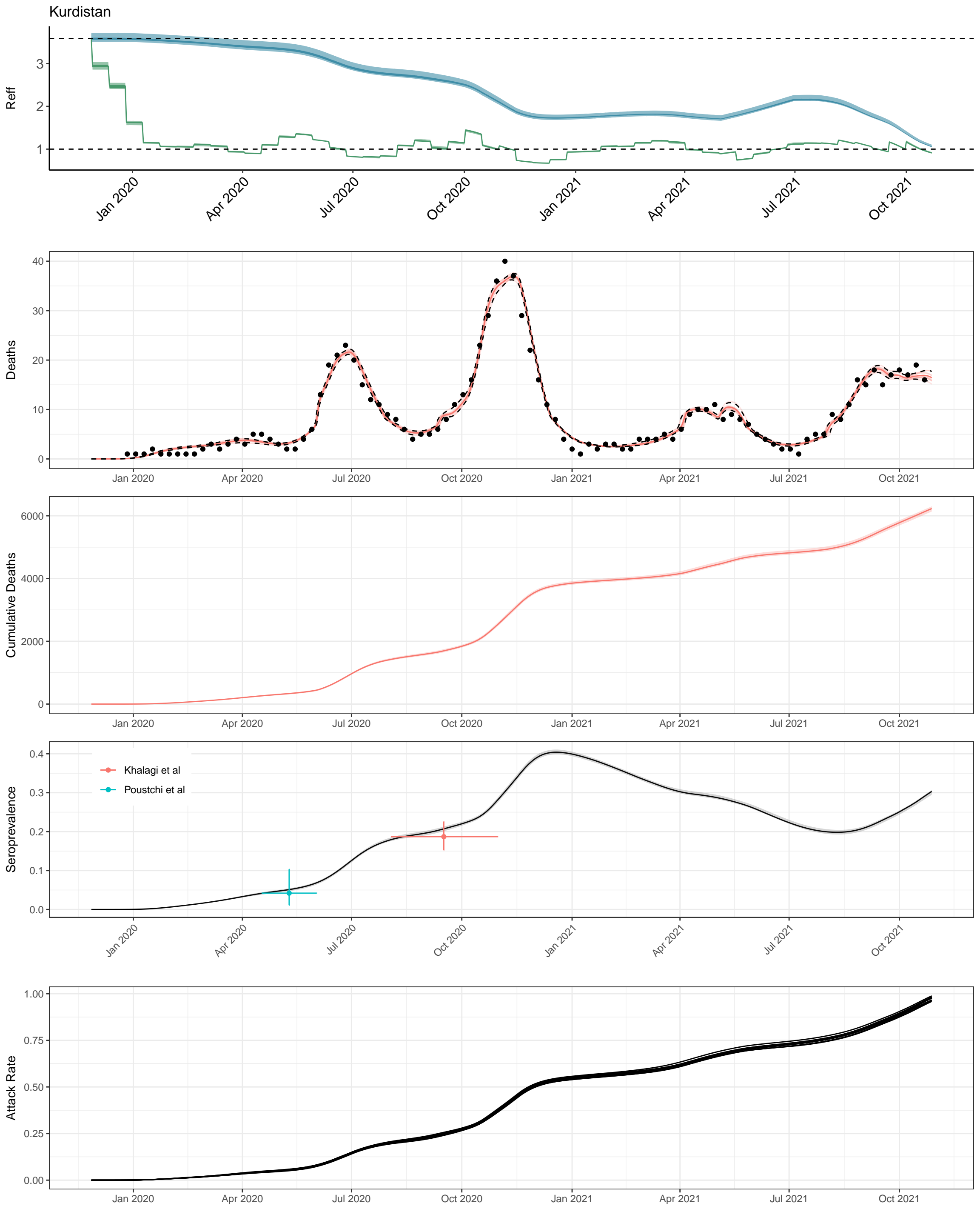

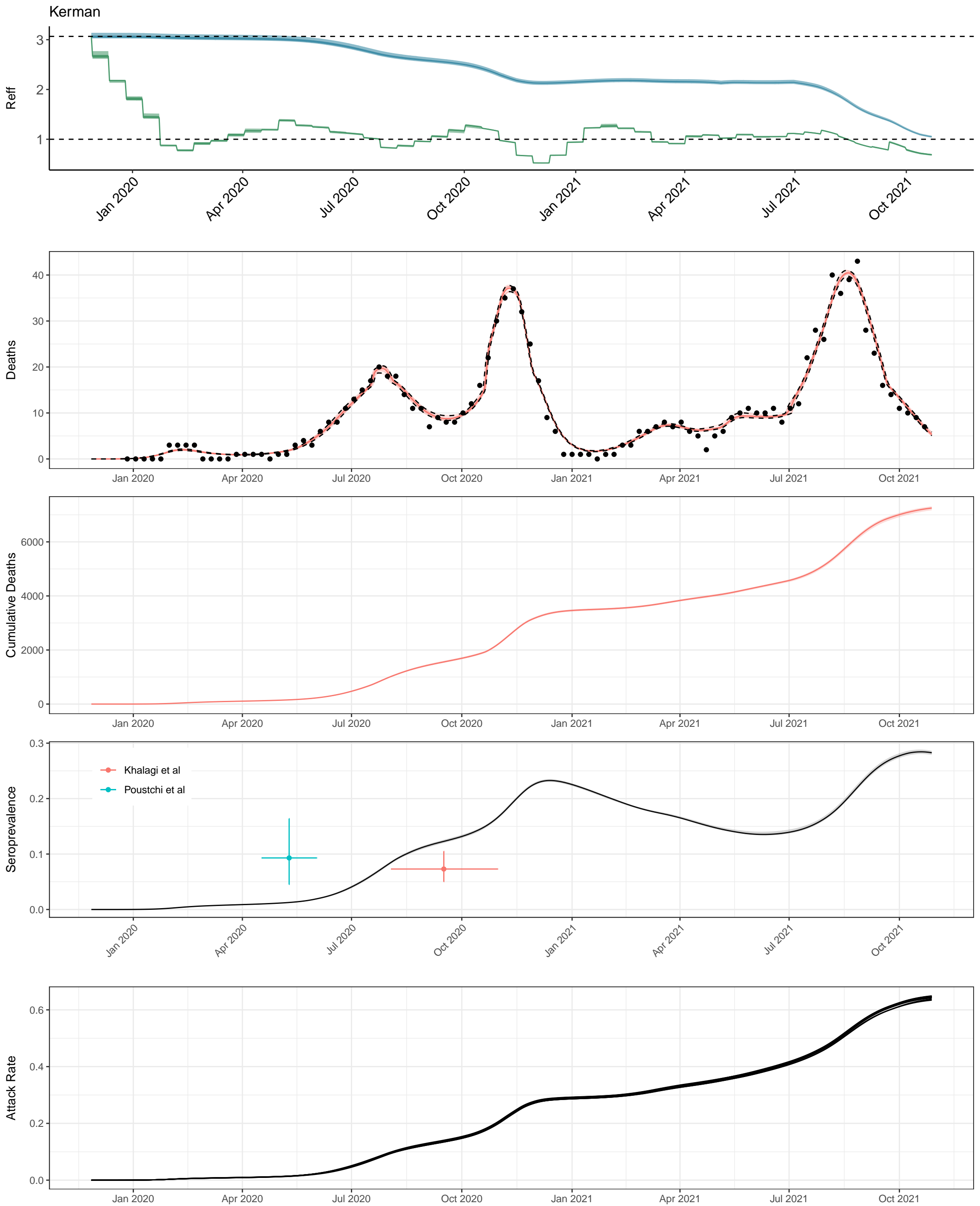

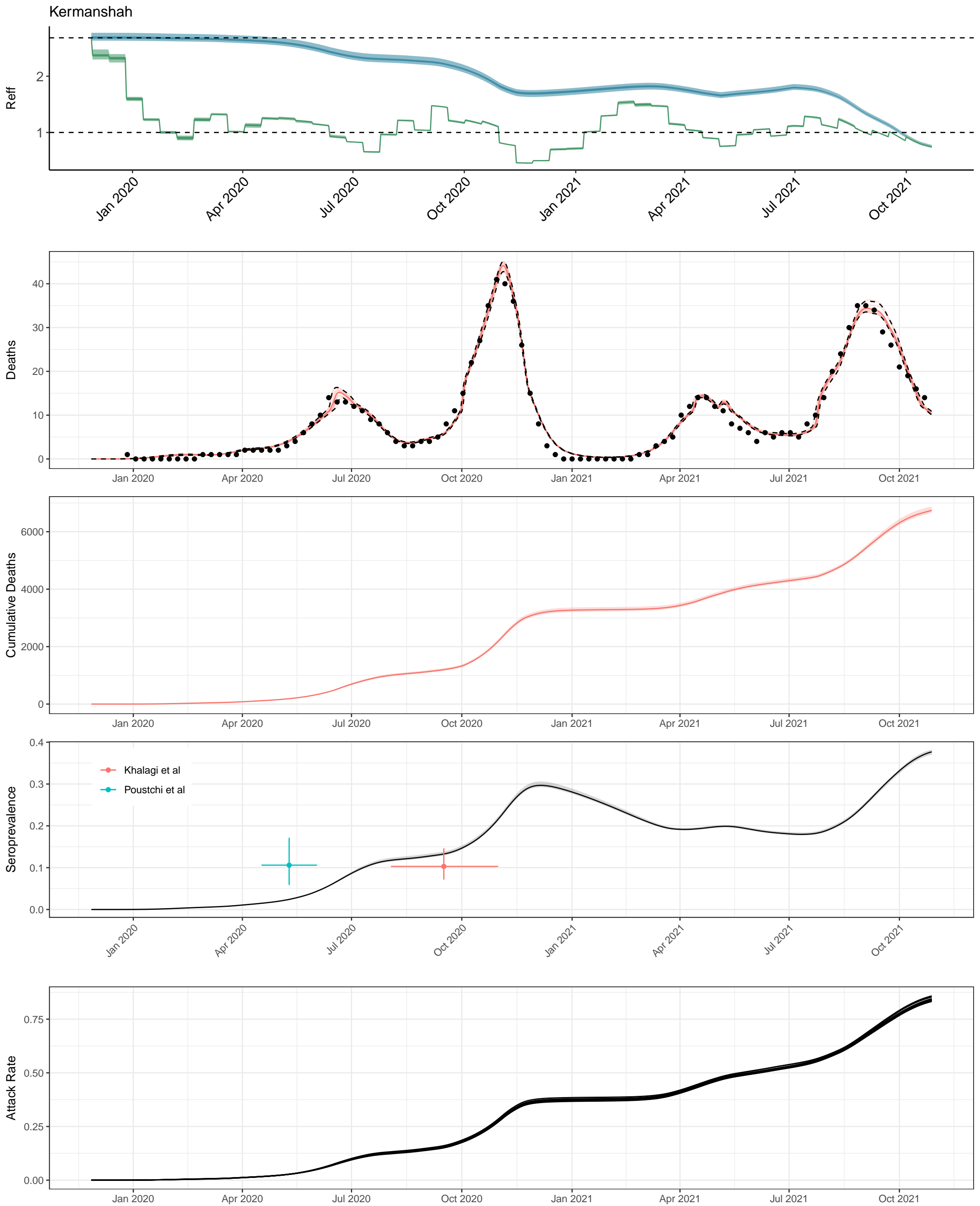

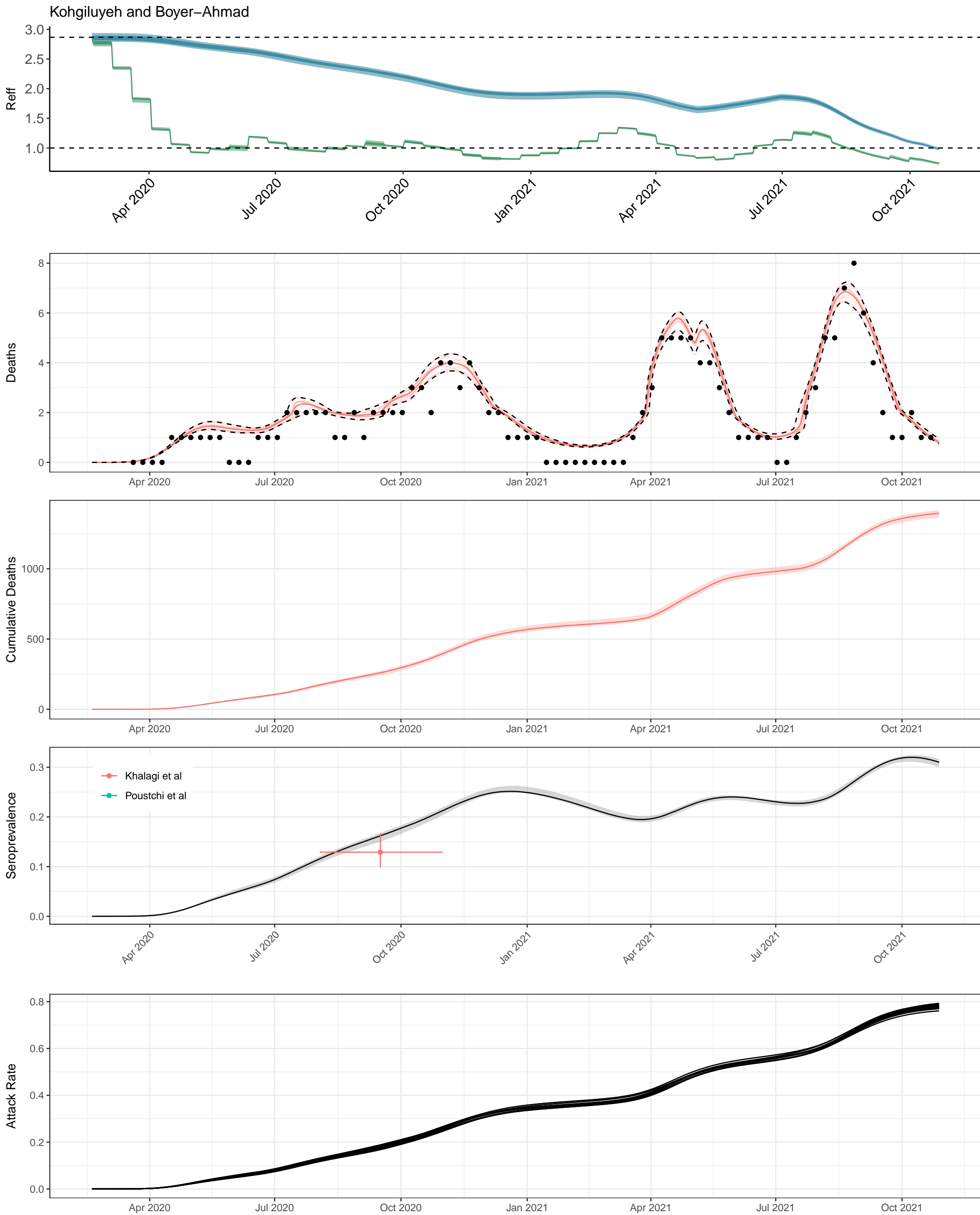

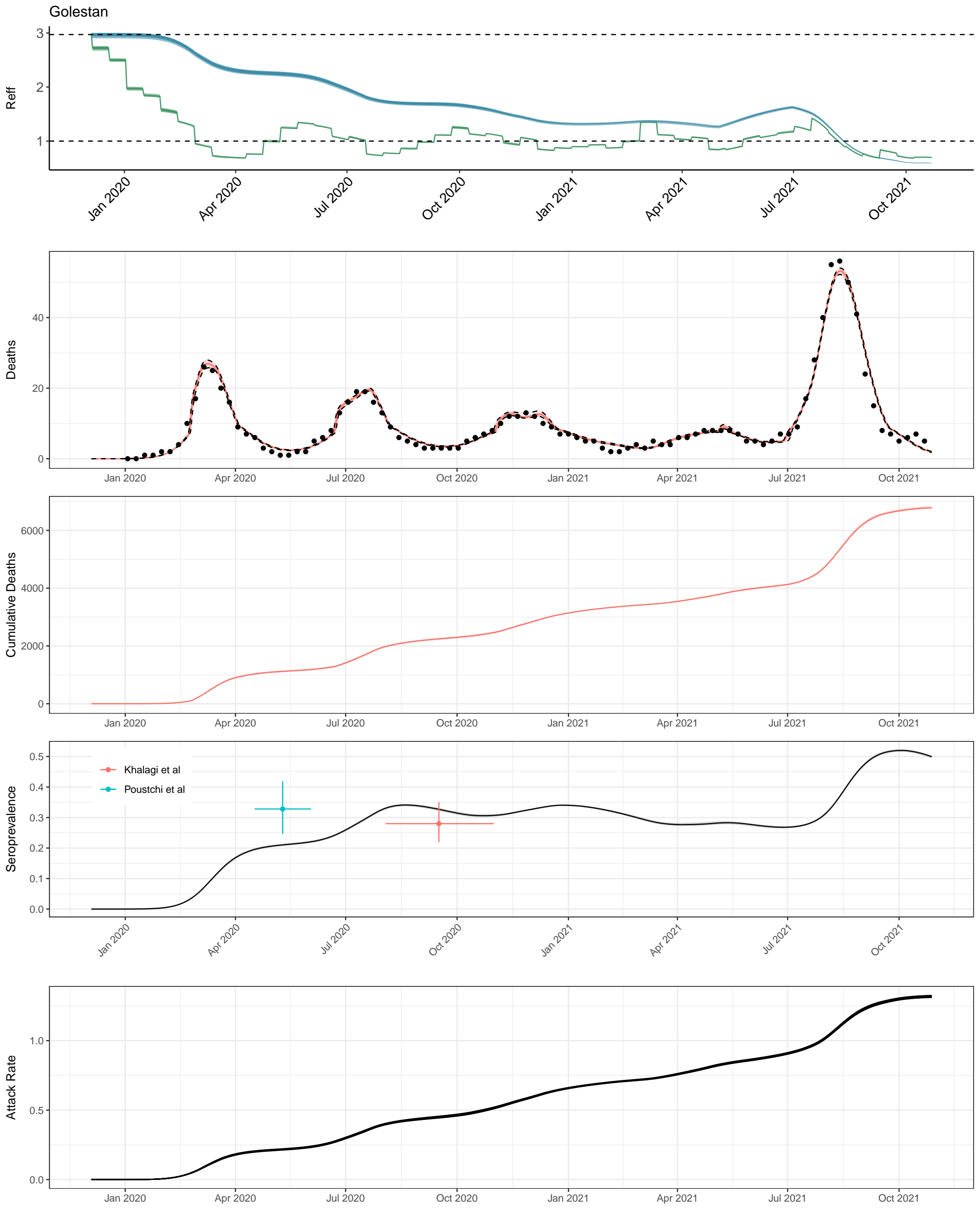

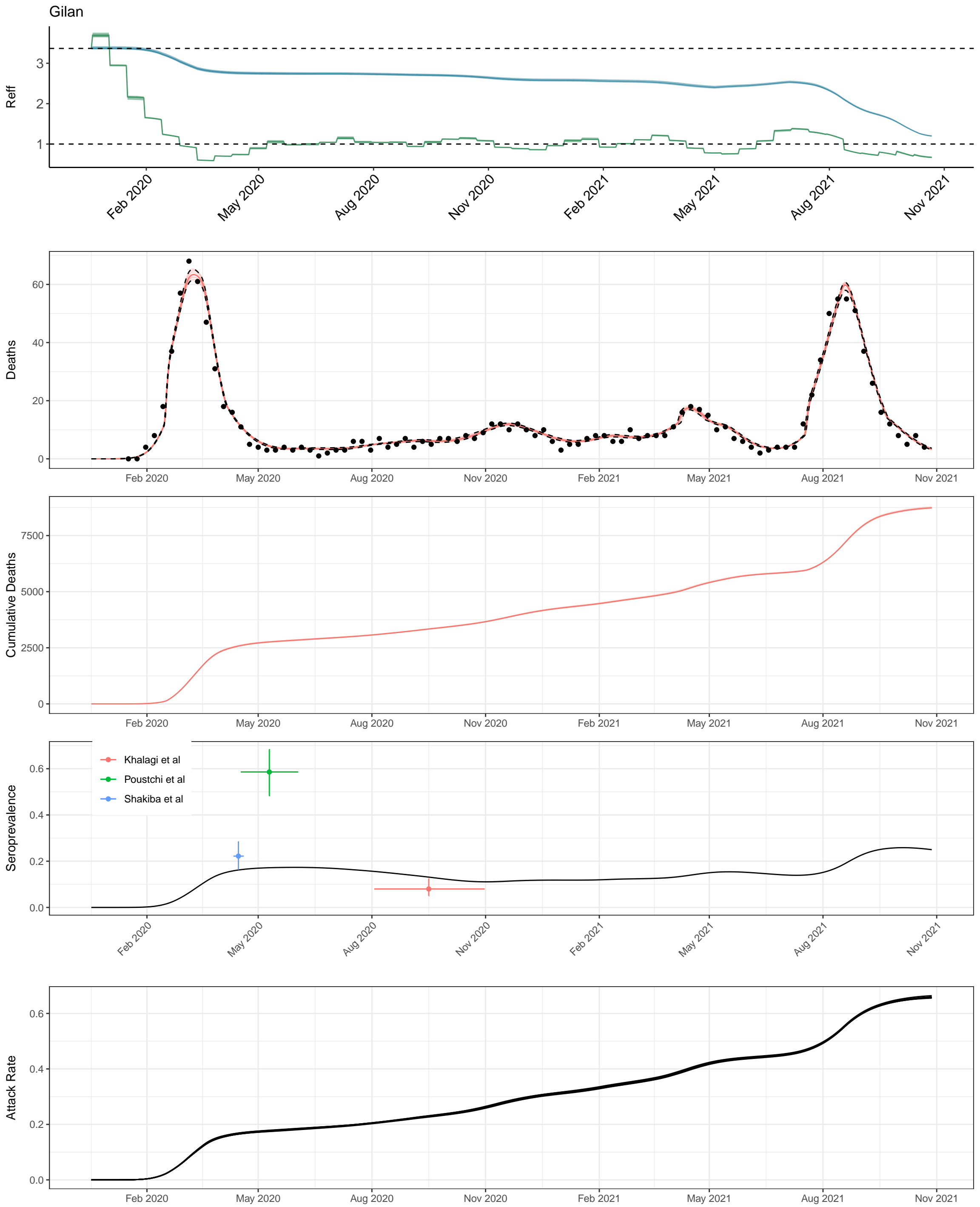

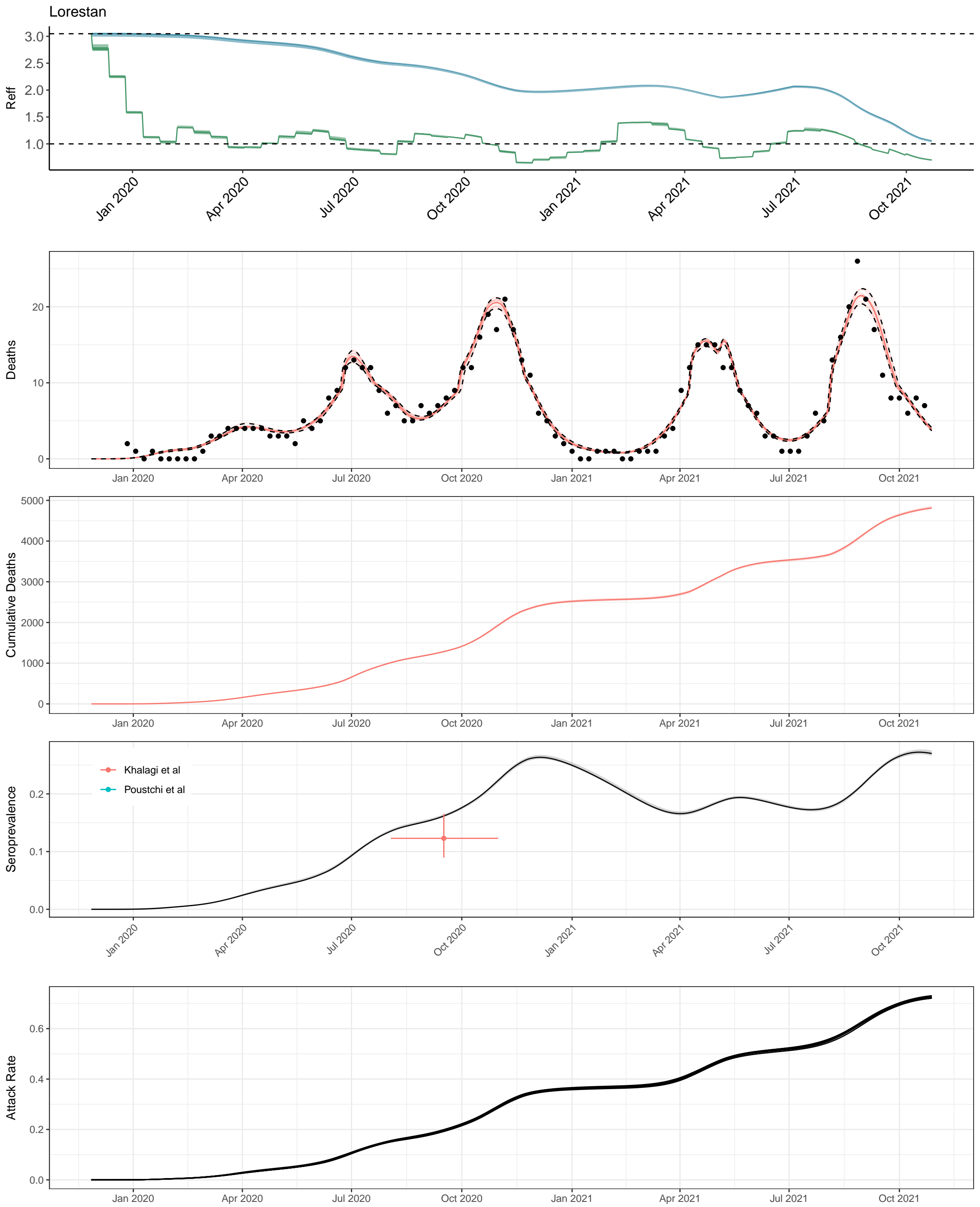

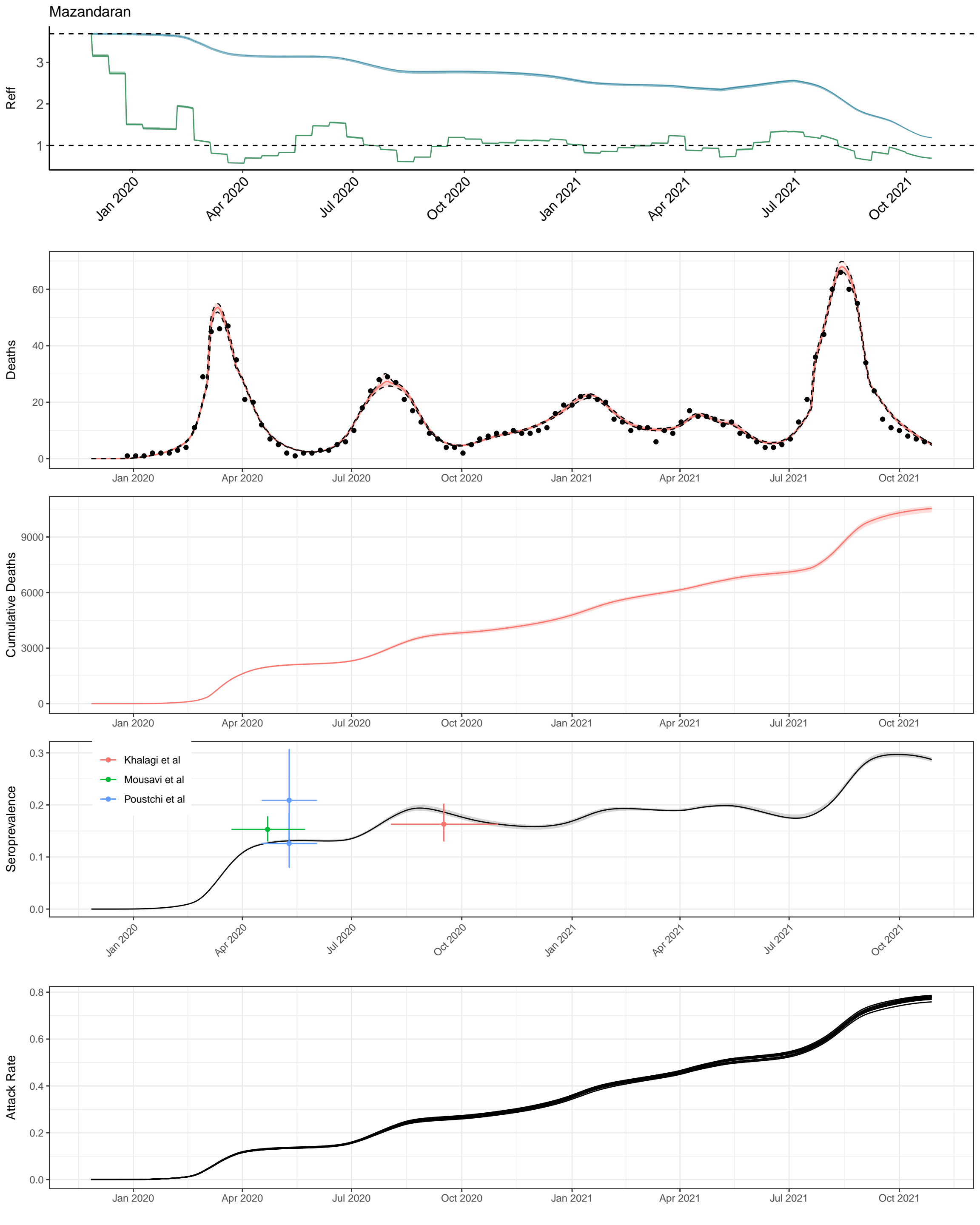

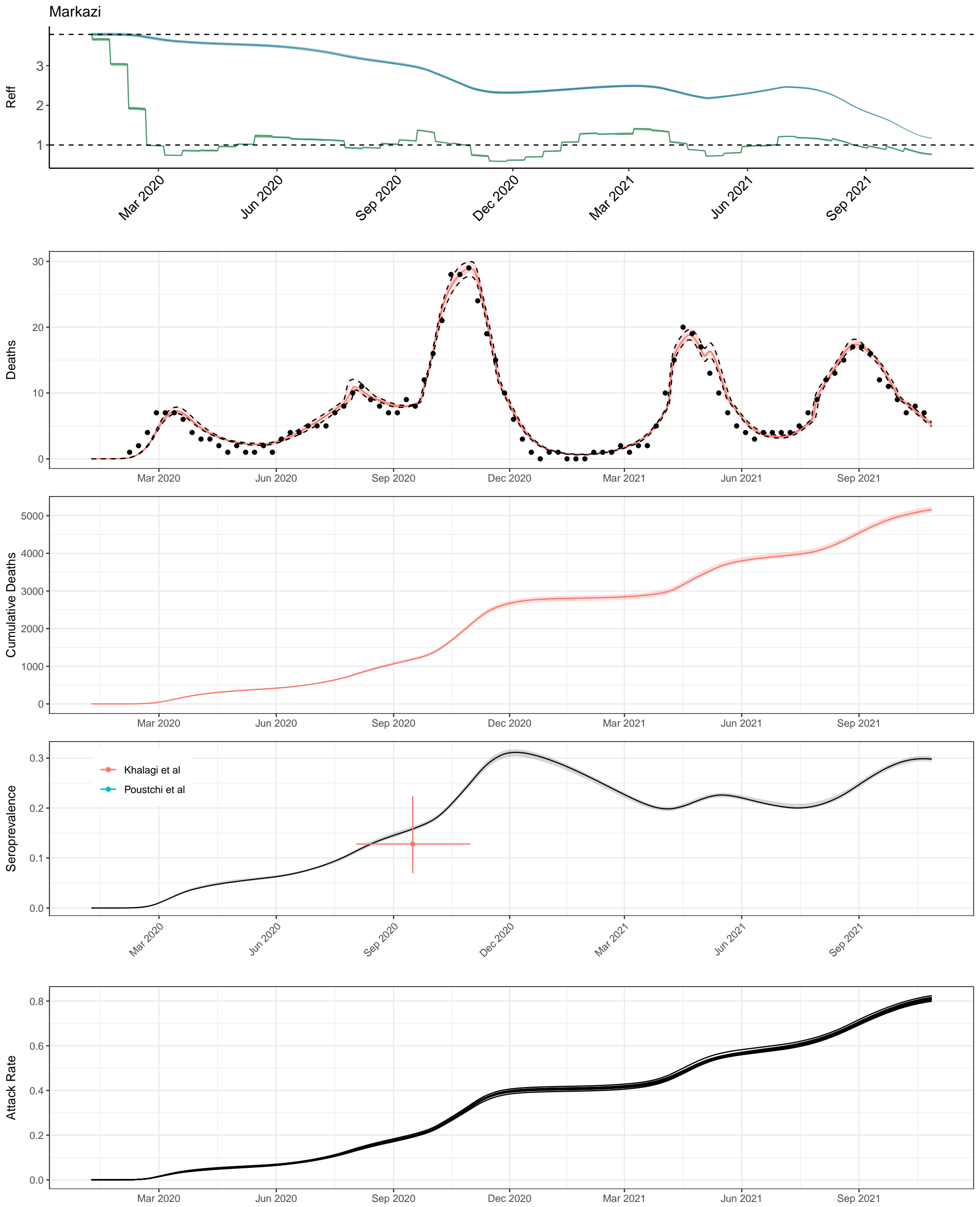

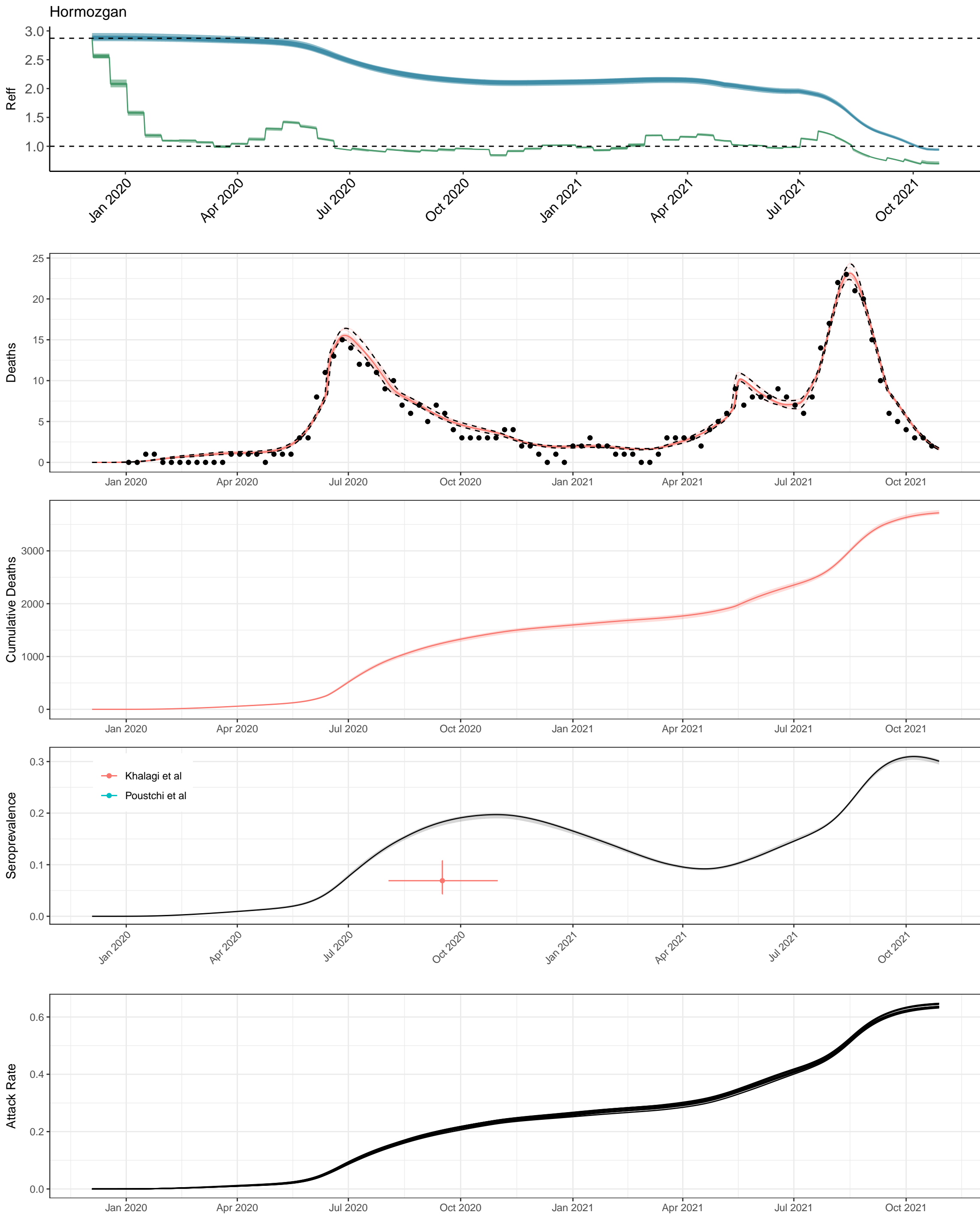

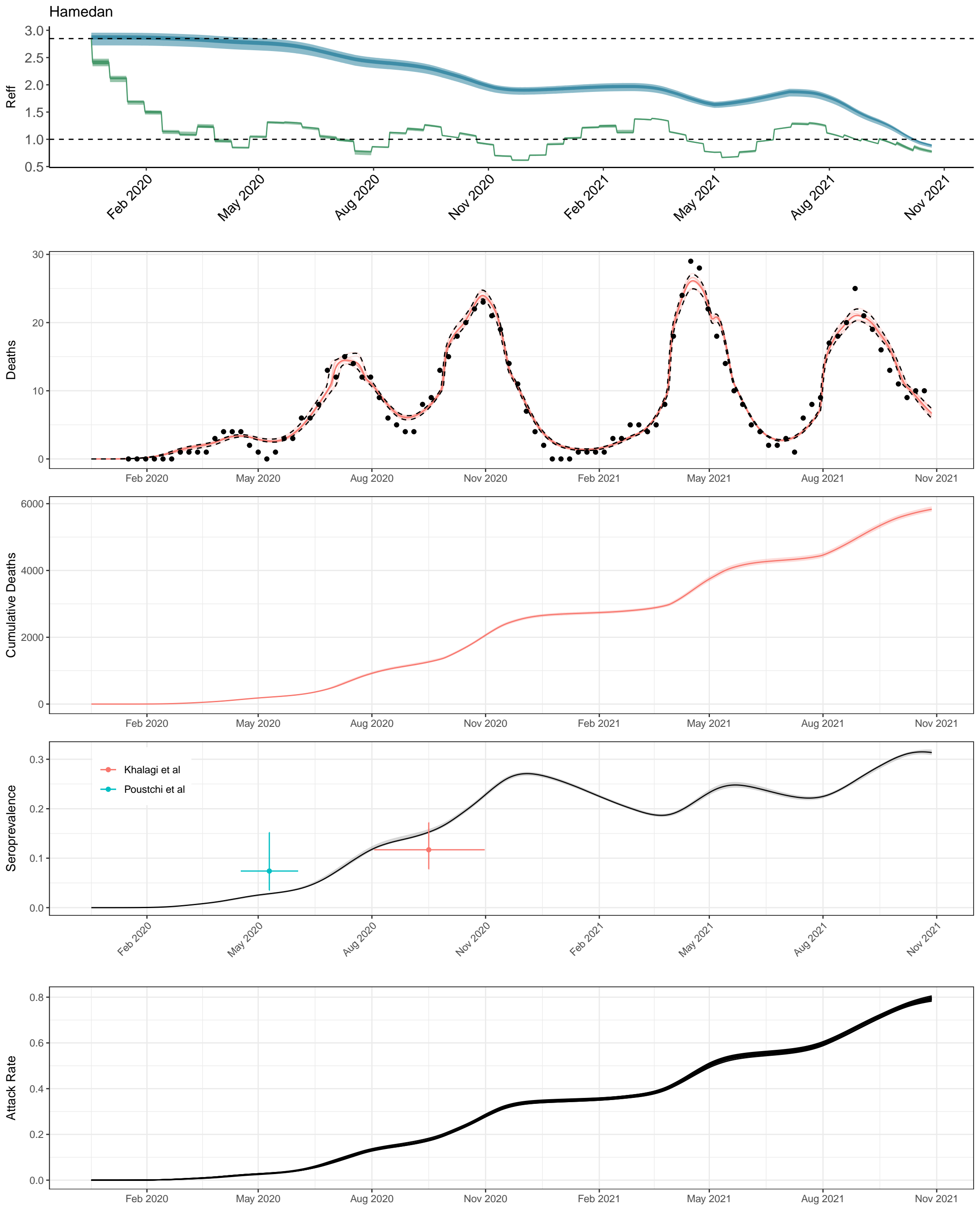

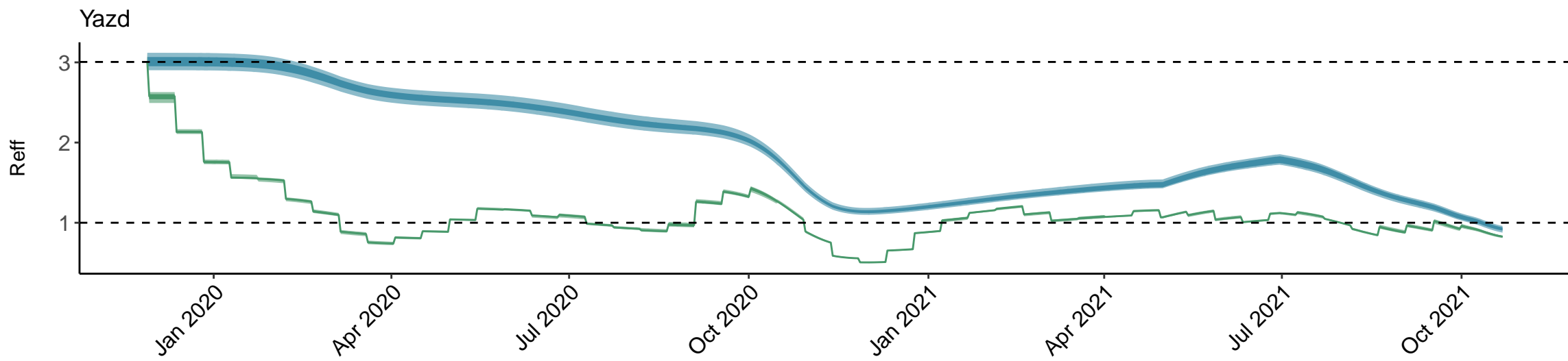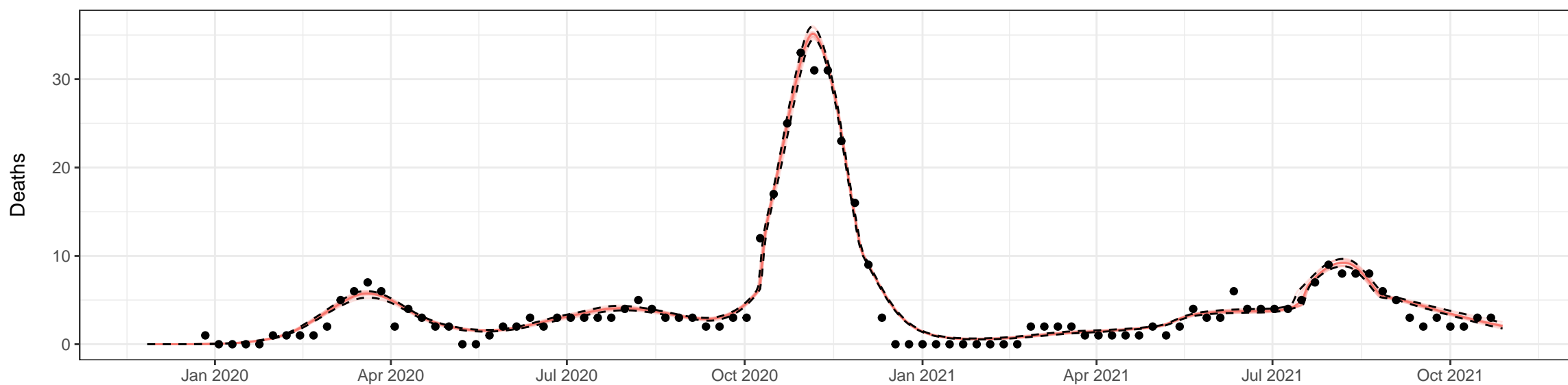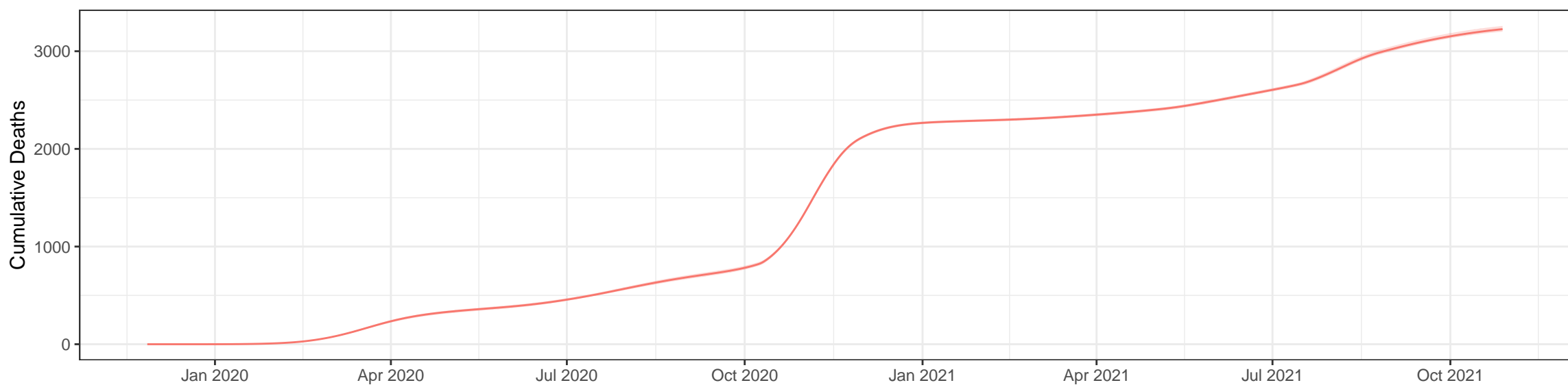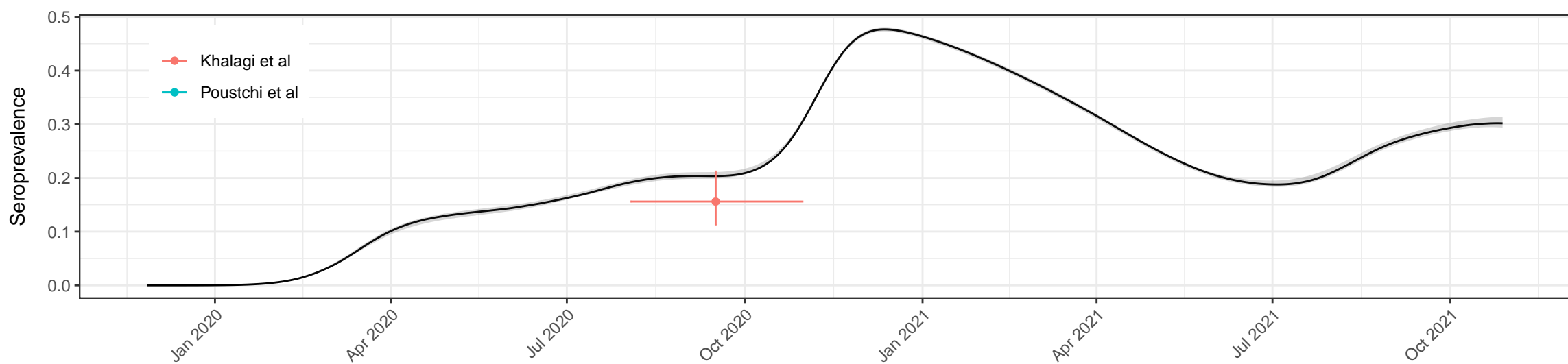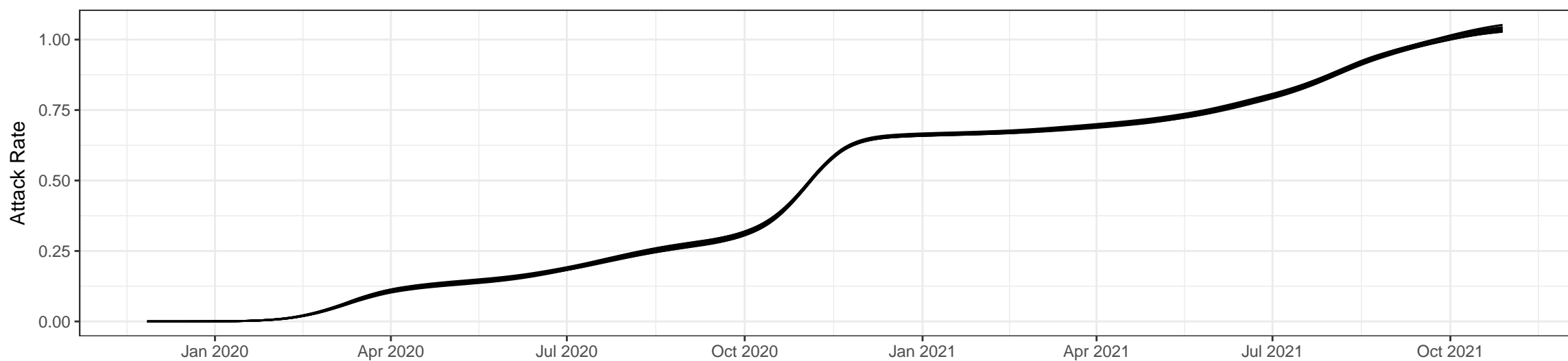

Supplement: Supplementary file 7 — Supplementary Data 4 [file 41467_2022_30711_MOESM7_ESM.pdf]
