# Supplementary material for: A framework for reconstructing SARS-CoV-2 transmission dynamics using excess mortality data
Source: Nat Commun. 2022 May 31;13:3015. doi: 10.1038/s41467-022-30711-y (PMC9156676; doi:10.1038/s41467-022-30711-y)
Supplement: Supplementary file 10 — Reporting Summary [file 41467_2022_30711_MOESM10_ESM.pdf]

nature portfolio

Corresponding author(s): Mahan Ghafari; Aris Katzourakis

Last updated by author(s): Apr 18, 2022

Reporting Summary

Nature Portfolio wishes to improve the reproducibility of the work that we publish. This form provides structure for consistency and transparency in reporting. For further information on Nature Portfolio policies, see our Editorial Policies and the Editorial Policy Checklist.

Statistics

For all statistical analyses, confirm that the following items are present in the figure legend, table legend, main text, or Methods section.

n/a	Confirmed	
☐	☒	The exact sample size (*n*) for each experimental group/condition, given as a discrete number and unit of measurement
☒	☐	A statement on whether measurements were taken from distinct samples or whether the same sample was measured repeatedly
☐	☒	The statistical test(s) used AND whether they are one- or two-sided<br>*Only common tests should be described solely by name; describe more complex techniques in the Methods section.*
☒	☐	A description of all covariates tested
☒	☐	A description of any assumptions or corrections, such as tests of normality and adjustment for multiple comparisons
☐	☒	A full description of the statistical parameters including central tendency (e.g. means) or other basic estimates (e.g. regression coefficient) AND variation (e.g. standard deviation) or associated estimates of uncertainty (e.g. confidence intervals)
☐	☒	For null hypothesis testing, the test statistic (e.g. *F*, *t*, *r*) with confidence intervals, effect sizes, degrees of freedom and *P* value noted<br>*Give P values as exact values whenever suitable.*
☐	☒	For Bayesian analysis, information on the choice of priors and Markov chain Monte Carlo settings
☒	☐	For hierarchical and complex designs, identification of the appropriate level for tests and full reporting of outcomes
☒	☐	Estimates of effect sizes (e.g. Cohen's *d*, Pearson's *r*), indicating how they were calculated

Our web collection on statistics for biologists contains articles on many of the points above.

Software and code

Policy information about availability of computer code

Data collection	Data collection and processing was performed using the statistical software R.<br>A "Data Availability" section is included in the manuscript, which describes all data used by and generated from this analysis available in an R research compendium at https://github.com/OJWatson/iran-ascertainment . This GitHub repository has been tagged and released as Watson, O. J. iran-ascertainment: v0.1.2(2022). doi: 10.5281/zenodo.6475585
Data analysis	A "Code Availability" section is included in the manuscript which describes the codes and analyses carried out in this study which are available at this analysis available in an R research compendium at https://github.com/OJWatson/iran-ascertainment . This GitHub repository has been tagged and released as Watson, O. J. iran-ascertainment: v0.1.2(2022). doi: 10.5281/zenodo.6475585. The repository is a reproducible research compendium and includes all R software packages used in the analysis of calculating excess mortality and constructing the transmission model.<br>Full list of all R (version 4.1.1) packages are available from the compendium.

For manuscripts utilizing custom algorithms or software that are central to the research but not yet described in published literature, software must be made available to editors and reviewers. We strongly encourage code deposition in a community repository (e.g. GitHub). See the Nature Portfolio guidelines for submitting code & software for further information.

Data

Policy information about availability of data

All manuscripts must include a data availability statement. This statement should provide the following information, where applicable:

- Accession codes, unique identifiers, or web links for publicly available datasets
- A description of any restrictions on data availability
- For clinical datasets or third party data, please ensure that the statement adheres to our policy

- Raw weekly mortality data files per province in Iran is available here:
https://www.sabteahval.ir/avej/Page.aspx?mId=49826&ID=3273&Page=Magazines/SquareshowMagazine
- Number of active beds in hospital wards by province in year 1396 solar hijiri (2018) is available here:
https://iranopendata.org/en/dataset/number-of-active-beds-in-hospital-wards-by-province-in-year-1396/resource/73e29d0b-7cc9-47c0-8b4e-b3e61cc0b800
- Number of administered vaccine doses per province (behdasht.gov) is available here:
https://t.me/it_behdasht/134
- Province-specific demography, with the population size in 5-year age bands, is available from the Statistical Center of Iran:
https://www.amar.org.ir/english
- daily hospital admissions per province (mask application, in collaboration with MoHME):
https://t.me/mask_application/438

Field-specific reporting

Please select the one below that is the best fit for your research. If you are not sure, read the appropriate sections before making your selection.

☒ Life sciences ☐ Behavioural & social sciences ☐ Ecological, evolutionary & environmental sciences

For a reference copy of the document with all sections, see nature.com/documents/nr-reporting-summary-flat.pdf

Life sciences study design

All studies must disclose on these points even when the disclosure is negative.

Sample size	Our study involves age-stratified weekly all-cause mortality data for all 31 provinces of Iran during the last 7 years in Solar Hijri calendar (1394, 1395, 1396, 1397, 1398, 1399, and 1400).
Data exclusions	The all-cause mortality data from 5 years prior to the start of the COVID-19 pandemic was used for the analysis (i.e., 1394, 1395, 1396, 1397, 1398 Solar Hijri). Year 1394 SH is excluded from the calculation of background mortality in Chahaar Mahal Bakhtiari province. This is because, as previous studies have shown (https://doi.org/10.1016/j.ijid.2021.04.015), there was a three-fold increase in registered deaths during this year compared to all the following years which, if included in the calculation of background deaths, would significantly bias the estimation of excess mortality for this province.
Replication	The software included in publication can be used to reproduce the findings.
Randomization	This is a population study fitting epidemic trajecotries to the population as one group and thus ramdomization is not applicable to the study and we did not conduct any analyses requiring randomization.
Blinding	The data was made public by the National Organization for Civil Registration of Iran and not collected by us. Our study is observational and not experimental. We did not use data from individual participants, rather the cumulative data on mortality and population of Iran was used. Therefore, blinding was not applicable to our analysis.

Reporting for specific materials, systems and methods

We require information from authors about some types of materials, experimental systems and methods used in many studies. Here, indicate whether each material, system or method listed is relevant to your study. If you are not sure if a list item applies to your research, read the appropriate section before selecting a response.

Materials & experimental systems

n/a	Involved in the study	
☒	☐	Antibodies
☒	☐	Eukaryotic cell lines
☒	☐	Palaeontology and archaeology
☒	☐	Animals and other organisms
☒	☐	Human research participants
☒	☐	Clinical data
☒	☐	Dual use research of concern

Methods

n/a	Involved in the study	
☒	☐	ChIP-seq
☒	☐	Flow cytometry
☒	☐	MRI-based neuroimaging
